# Near Infared Circularly Polarized Luminescence From Water Stable Organic Nanoparticles Containing a Chiral Yb(III) Complex

**DOI:** 10.1002/chem.202200574

**Published:** 2022-05-23

**Authors:** Enrico Cavalli, Chiara Nardon, Oliver G. Willis, Francesco Zinna, Lorenzo Di Bari, Silvia Mizzoni, Silvia Ruggieri, Salvatore C. Gaglio, Massimiliano Perduca, Claudio Zaccone, Alessandro Romeo, Fabio Piccinelli

**Affiliations:** ^1^ Department of Chemical Sciences Life and Environmental Sustainability Parma University Parco Area delle Scienze, 11/a 43124 Parma Italy; ^2^ Luminescent Materials Laboratory DB Verona University Strada Le Grazie 15 37134 Verona Italy; ^3^ Department of Chemistry and Industrial Chemistry Pisa University via Moruzzi 13 56124 Pisa Italy; ^4^ Biocrystallography Lab Department of Biotechnology Verona University Strada Le Grazie 15 37134 Verona Italy; ^5^ Department of Biotechnology Verona University Strada Le Grazie 15 37134 Verona Italy; ^6^ Department of Computer Science Verona University Strada Le Grazie 15 37134 Verona Italy

**Keywords:** circularly polarized luminescence, lanthanide complexes, luminescence, nanoparticles, optical imaging

## Abstract

We report the first example of very efficient NIR Circularly Polarized Luminescence (CPL) (around 970 nm) in water, obtained thanks to the combined use of a chiral Yb complex and of poly lactic‐co‐glycolic acid (PLGA) nanoparticles. [Yb**L**(tta)_2_]CH_3_COO (**L**=N, N’‐bis(2‐pyridylmethylidene)‐1,2‐(*R,R*+*S,S*) cyclohexanediamine and tta=2‐thenoyltrifluoroacetonate) shows good CPL in organic solvents, because the tta ligands efficiently sensitize Yb NIR luminescence and the readily prepared chiral ligand **L** endows the complex with the necessary dissymmetry. PLGA nanoparticles incorporate the complex and protect the metal ion from the intrusion of solvent molecules, while ensuring biocompatibility, water solubility and stability to the complex. Hydrophilic NIR‐CPL optical probes can find applications in the field of NIR‐CPL bio‐assays.

## Graphical abstract

**Unprecedented NIR CPL** activity in water from a hydrophobic chiral Yb(III) complex embedded in PLGA nanoparticles was observed

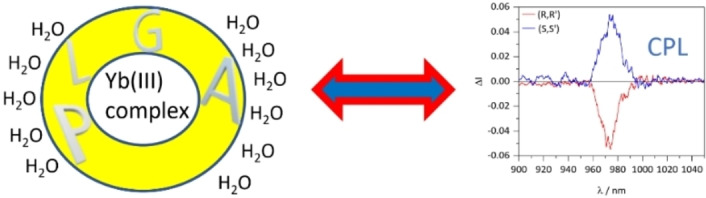

## Introduction

Current applications of circularly polarized luminescence (CPL) in bioimaging would greatly benefit from its extension and consolidation in the near‐infrared (NIR) region, where tissues, skin, blood and water are quite transparent and scattering is also relatively low. The 1000–1400 nm range is sometimes called the second biological window and it is emerging as a very attractive region for biological imaging. CPL in this region benefits from the specificity, selectivity and sensitivity, which are typical of the chiroptical counterpart of emission spectroscopy, in the context of biological applications, like, for example microscopy and bioassays.^[1]^

Complexes of NIR emitting lanthanide ions like Yb(III), having small size and metal‐centred emission properties, may provide good candidates, thanks to the 1 μm emission band of Yb(III), associated with its low toxicity and to the well‐known chemistry developed for using other lanthanides in biomedicine.

Moreover, in addition to biomedical applications, Yb‐centred NIR‐CPL may lend itself to several other technological fields, where vis‐CPL allied to Eu or Tb have already demonstrated a positive impact.^[2]^

NIR‐CPL activity is indeed expected in the case of chiral Yb(III)‐based complexes in which the spin allowed ^2^F_5/2_→^2^F_7/2_ emission transition, around 980 nm, is characterized by a high value of the rotational strength^[3]^ and can be conveniently sensitized by means of a ligand to metal energy transfer (*antenna effect*).^[4]^

**Figure 1:**
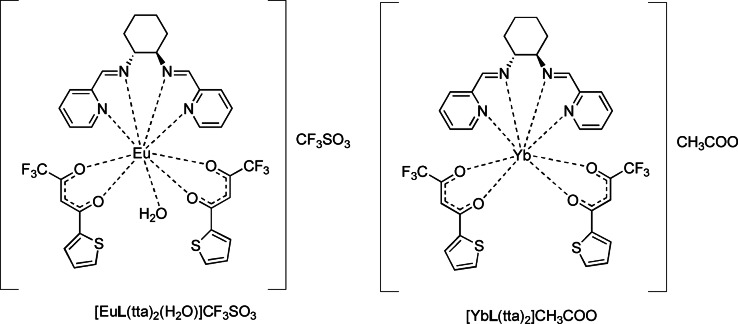
Molecular structure of the complexes discussed in this contribution. The *R,R* isomer of L ligand is reported.

Only few examples of such complexes have been reported so far within the literature.^[5]^ In order to switch to practical applications of NIR‐CPL in the field of bio‐assays, it would be important to devise systems that can be dissolved/dispersed in aqueous media while still retaining their (chiro)optical properties. With Yb(III)‐based complexes this is challenging, because the emission efficiency of Yb(III) complexes in solution can be significantly affected by multiphonon relaxation (MPR) processes involving the stretching vibrations of the X−H bonds (X=C, N, O) present in the coordinated ligands and solvent molecules.^[6]^ In aqueous solution, water molecules can reduce the ^2^F_5/2_ excited state lifetime to only few μs^[7]^ or shorter, even if only being present in the outer coordination sphere of an Yb(III) ion. When water is absent from both the inner and outer coordination spheres, lifetime values can attain 12–23 μs, partnered with higher quantum yields. This exclusion of water has been achieved in cases such as a bi‐capped Yb(III) ion, utilizing a porphyrin based double‐decker^[8]^ or of hydrophobic complexes.^[9]^ The detrimental effect of MPR can be significantly limited by embedding the luminescent complexes in polymeric nanoparticles. Previous studies have demonstrated that poly lactic‐co‐glycolic acid (PLGA), a polymer with good biodegradability and biocompatibility properties, is an attractive candidate for encapsulating and thus protecting organic molecules and metal complexes. In fact, once [Eu**L**(tta)_2_(H_2_O)]CF_3_SO_3_ complex, where **L** stand for N, N’‐bis(2‐pyridylmethylidene)‐1,2‐(*R,R* or *S,S*) cyclohexanediamine, respectively (Figure 1), is embedded in PLGA, it shows, upon excitation of tta, the same metal centered luminescence efficiency encountered in the free complex dissolved in organic solvent.^[10]^

Unfortunately, its sizeable CPL activity in dichloromethane solution (g_lum_=0.23 around 590 nm)^[11]^ was completely lost in the polymeric nanoparticles dispersed in water. Surprisingly, the PLGA matrix shows a double beneficial effect on the Yb(III) counterpart ([Yb**L**(tta)_2_]CH_3_COO complex. With respect to the free [Yb**L**(tta)_2_]CH_3_COO molecule dissolved in dichloromethane (DCM), the water stable nanoparticles loaded with the complex possess a similar Yb(III) luminescence efficiency, while the CPL activity appears almost doubled. In other words, to the best of our knowledge, [Yb**L**(tta)_2_]CH_3_COO represents the first case of a chiral Yb(III) complex embedded in PLGA nanoparticles (NPs) stable in water and exhibiting efficient NIR emission with a good degree of polarization. In detail, this paper deals with the characterization and chiroptical investigation in solid state, organic solution (DCM) and in water dispersible PLGA nanoparticles of the ([Yb**L**(tta)_2_]CH_3_COO complex.

## Results and Discussion

**Figure 2:**
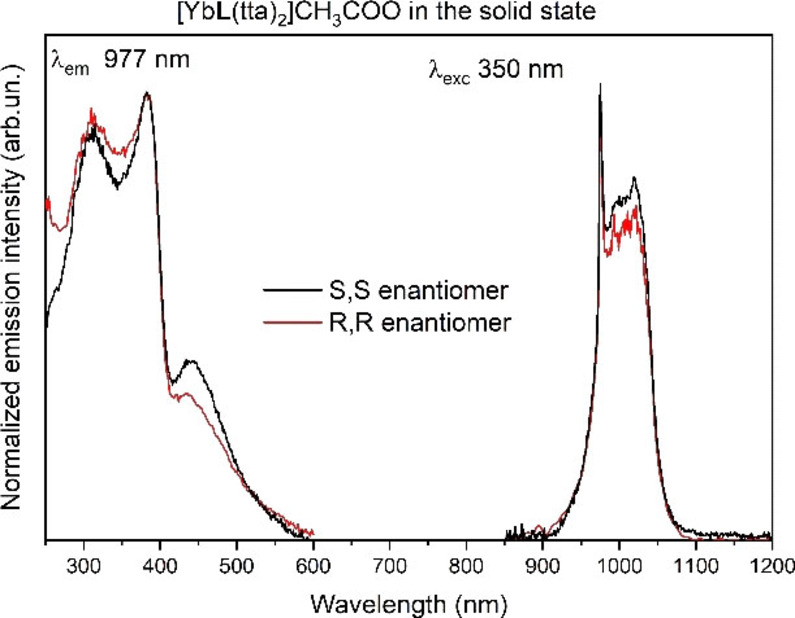
Excitation spectra (left) and emission spectra (right) of both enantiomers of [YbL(tta)_2_] ⋅ CH_3_COO complex, in the solid state. R,R (red line); S,S (black line).

The expected chemical formula ([Yb**L**(tta)_2_]CH_3_COO) of the synthesized complex is in agreement with the elemental analysis results (see experimental section), which together with the TGA‐DSC analysis (Figure S1, Supporting Information) and ESI mass spectrometry (see experimental section) rule out the presence of water molecules either in the inner or outer coordination spheres of the metal ion, in the powdered sample. In fact, up to 150 °C, no significant weight loss (<0.7 %) as well as no endothermic peak are observed (Figure S1). On the contrary, a significant weight loss (around 55 %), related to the thermal decomposition of the organic matter, is detected in the 200–600 °C range. Lastly, an endothermic event is recorded at temperature >600 °C due to the shift from N_2_ to air resulting in the complete combustion of the material. At 700 °C, almost 20 % of the original mass is recovered in agreement with the residual presence of the only Yb_2_O_3_. For both the enantiomers, Yb(III) ion efficiently emits light in the NIR spectral region, upon excitation around 365–380 nm, in correspondence with the diketonate‐centred singlet‐singlet π‐π* enolic transition of tta. Typical Yb(III) emission (Figure 2) can be observed, consisting of a sharp zero‐line peak at 977 nm and a manifold in the 900–1100 nm region, associated to the crystal field splitting of the ^2^F_7/2_ ground state.

The excitation spectra present other two maxima peaking at 310 and 450 nm. While the former band is associated to the *antenna effect* involving the chiral ligand, the latter unusual excitation band extended into the visible region has been already observed in the case of tta‐based complexes of Eu(III) ion^[12]^ and interpreted on the basis of an increased contribution of the ligand (tta) triplet state to the energy transfer process, occurring in the solid state. Nevertheless, the precise nature of this band, observed only in the solid state, is still an open question. The sensitization of the Yb(III) emission cannot be accounted for in the frame of the classical Förster‐Dexter Theory, in consequence of the large mismatch between the involved electronic levels. Two mechanisms have been invoked in this connection, the first one based on electron transfer involving the Yb(III)/Yb(II) redox couple and the excited state of the ligand,^[13]^ the second on the internal conversion inside the energy levels of the Yb(III) ligands system considered as a single chromophore.^[14]^ In the present case, the available information does not allow us to discriminate between the two proposed mechanisms.

**Figure 3:**
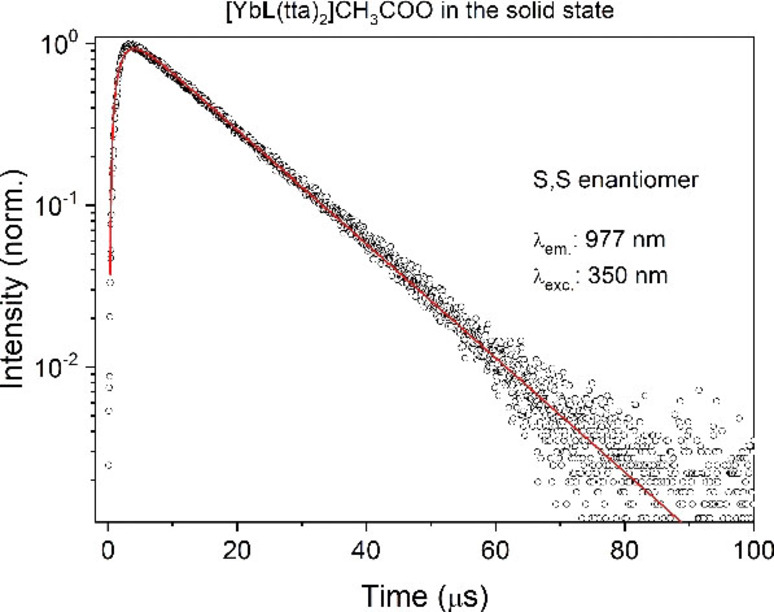
Decay curve of the ^2^F_5/2_ excited state of Yb(III) in the case of the S,S enantiomer of [YbL(tta)_2_] ⋅ CH_3_COO complex. The curve of the R,R enantiomer (not shown) is fully superimposable.

The decay profile of the emission (Figure 3) presents a build‐up and can be reliably reproduced by a difference of two exponential functions. The evaluated rise‐time (around 1.5 μs) is connected with the kinetics of the energy transfer between the ligand and Yb(III), whilst the decay constant of the tail, 12.3 μs, is the lifetime of the ^2^F_5/2_ excited state.

As underlined in the introduction and in agreement with the results obtained from the other experimental techniques, a lifetime value around 10 μs is compatible with the absence of water molecules (and other high energy oscillators) in the metal ion environment (inner and outer coordination sphere).

**Figure 4:**
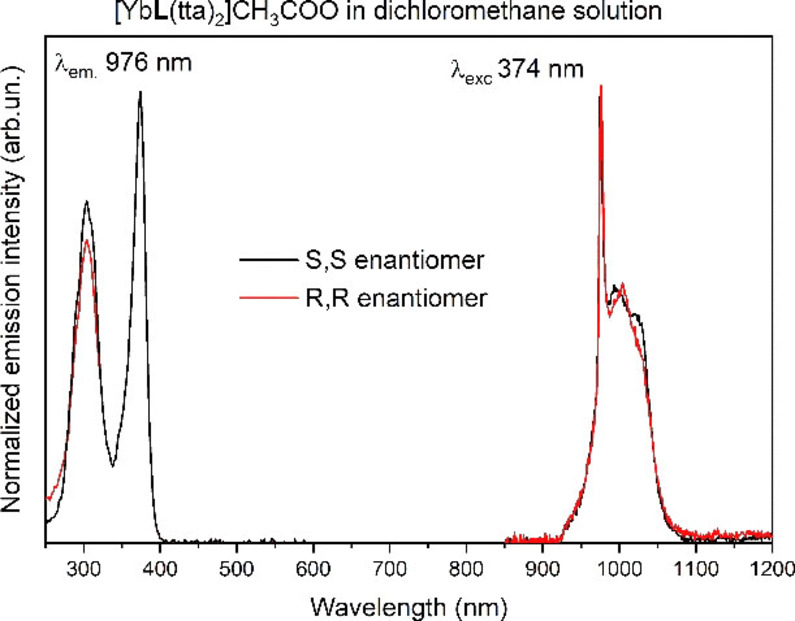
Excitation spectra (left) and emission spectra (right) of both enantiomers of [YbL(tta)_2_] ⋅ CH_3_COO complex, in 0.44 mM dichloromethane solution. R,R (red line); S,S (black line).

The emission spectrum recorded in a 0.44 mM DCM solution (Figure 4) is quite similar to those recorded in the solid state, whereas the excitation spectra show some differences.

First of all, the two higher energy components are narrower and better resolved than in Figure 2, and their relative intensity is slightly changed. Moreover, the weaker component in the 450 nm region has expectedly disappeared, as above commented. Several factors can account for these differences such as measurement conditions, solvent role, structural differences on passing from solid to solution (in terms of bond distances and angles). The decay curves of ^2^F_5/2_ level in dichloromethane solution (Figure S2) are similar to that recorded in the solid state. The rise time constant is 1.4 μs and the lifetime of the excited state is 15.1 μs and 12.3 μs, for the S,S and R,R enantiomers, respectively. The values of the lifetimes are comparable with that recorded in the solid state (12.3 μs). This indicates that the nature of the emitting complex is the same as that in the solid state and sensitization mechanism still involves the triplet state of the tta ligand.

Since, it has been shown that Y(III) complexes may serve as suitable computational models for the Ln(III) analogues, a detailed structural study performed by some of us on the Y(III)‐based counterpart ([Y**L**(tta)_2_]A; A=nitrate or triflate anion) suggests the presence in DCM solution of a couple of interconverting isomers possessing *C1* symmetry and differing by the relative orientation of the two tta ligands; in both cases the counterion is bound to the metal ion.^[11]^ On the other hand, NMR indicates effective *C2* symmetry (equivalence of the two tta molecules and of the halves of the chiral ligand). This demonstrates that the NMR spectra are in all cases averages due to fast equilibria between the isomers, a situation that prevents any quantitative analysis of the paramagnetic shifts. Furthermore, the results reported in that paper^[11]^ underline that the CPL activity of europium(III) and samarium(III) complexes is the result of the whole Ln(III) environment, not only characterized by the ligands, but also by the counterion and solvent molecules (i. e. methanol and acetonitrile in addition to DCM were investigated). The presence of equilibria interconverting solvated, anion coordinated complexes and isomers differing by the relative orientation of tta ligands affects some bond lengths involving the lanthanide ion and, in turn, the CPL activity of the complexes. It is therefore not surprising that [Eu**L**(tta)_2_(H_2_O)]CF_3_SO_3_ complex can completely loose the CPL activity once its surroundings move from the DCM solvent to the PLGA polymer. Similar effect of solvation on the CPL activity of coordinatively saturated chiral Eu(III) complexes has been recently reported by D. Parker et al.^[15]^

**Figure 5:**
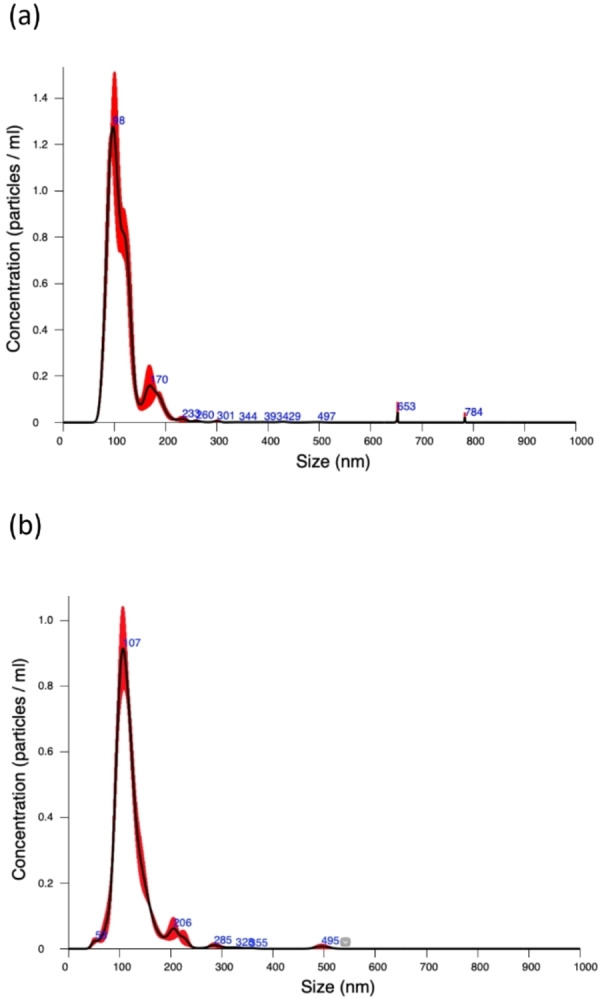
Nanotracking analysis (NTA) of **(a)** S,S and **(b)** R,R isomers of [YbL(tta)_2_] ⋅ CH_3_COO loaded nanoparticles. Diagrams are obtained as average of three independent measurements.

As for the strategy to make the [Yb**L**(tta)_2_]⋅CH_3_COO molecule stable in aqueous solution, we prepared a nanoformulation in which this complex is embedded in PLGA by means of a modified nanoprecipitation method at 20 °C.^[16]^ The obtained NPs show a monodispersed distribution of the size. By means of dynamic light scattering (DLS), the average size was around 118±3 and 102±3 nm for the R,R and S,S enantiomers, respectively. Nanotracking analysis (NTA) (Figure 5) and atomic force microscopy (AFM) experiments (Figure S3, Table S1 and S2) agree with the DLS results. The NPs show good colloidal stability in water with a negatively charged surface, the ζ‐Potential value being close to −13 mV.

**Figure 6:**
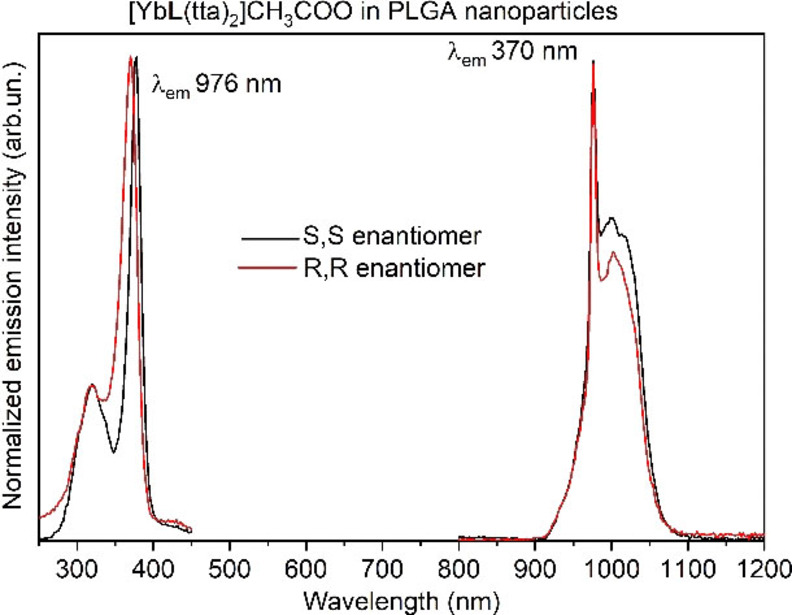
Excitation spectra (left) and emission spectra (right) of both enantiomers of [YbL(tta)_2_] ⋅ CH_3_COO complex embedded in PLGA nanoparticles, in aqueous solution. R,R (red line); S,S (black line).

The luminescence emission spectra are fully consistent to those recorded in DCM, suggesting that the complexes preserve their identities when incorporated in the PLGA matrix (Figure 6).

**Figure 7:**
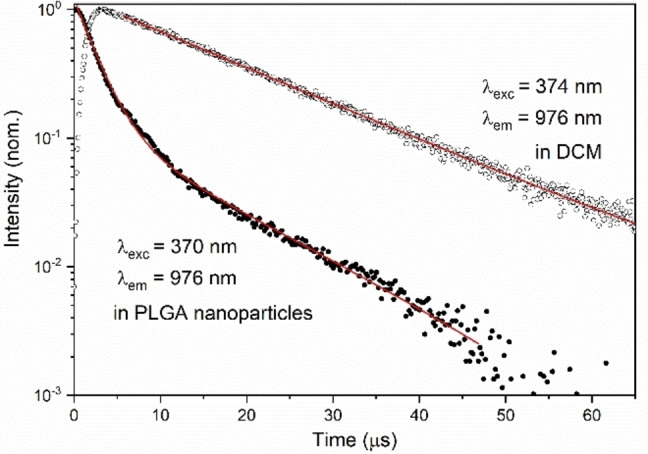
Comparison between the emission decay profiles of S,S‐[YbL(tta)_2_] ⋅ CH_3_COO in DCM solution and encapsulated in PLGA NPs in water solution.

As for the excitation spectrum, the higher energy component has lower intensity than in the spectra previously reported. (Figure 2 and 4). Furthermore, the luminescence decay profile of the complexes embedded in PLGA‐NPs is a double‐exponential (Figure 7 and S4) with the dominant long‐lived component having a time constant of 13 μs and 11.7 μs, for the S,S and R,R enantiomers, respectively; i. e. rather similar to that observed in DCM (Figure 7). The origin of the faster component of the decay has yet to be fully understood, even if it is reasonable to assume that it can be a consequence of interactions of the molecules of the complex with each other or with the donor groups (i. e. OH and COOH) of the PLGA polymer. Taken together, the results suggest that no water molecules are present either in the inner or outer coordination spheres of the metal ion also in the PLGA environment. Remarkably, after one week the NPs containing the complexes are still fairly emissive and the shape of the decay curves remains almost identical, even though a decrease of the luminescence intensity is detected.

**Figure 8:**
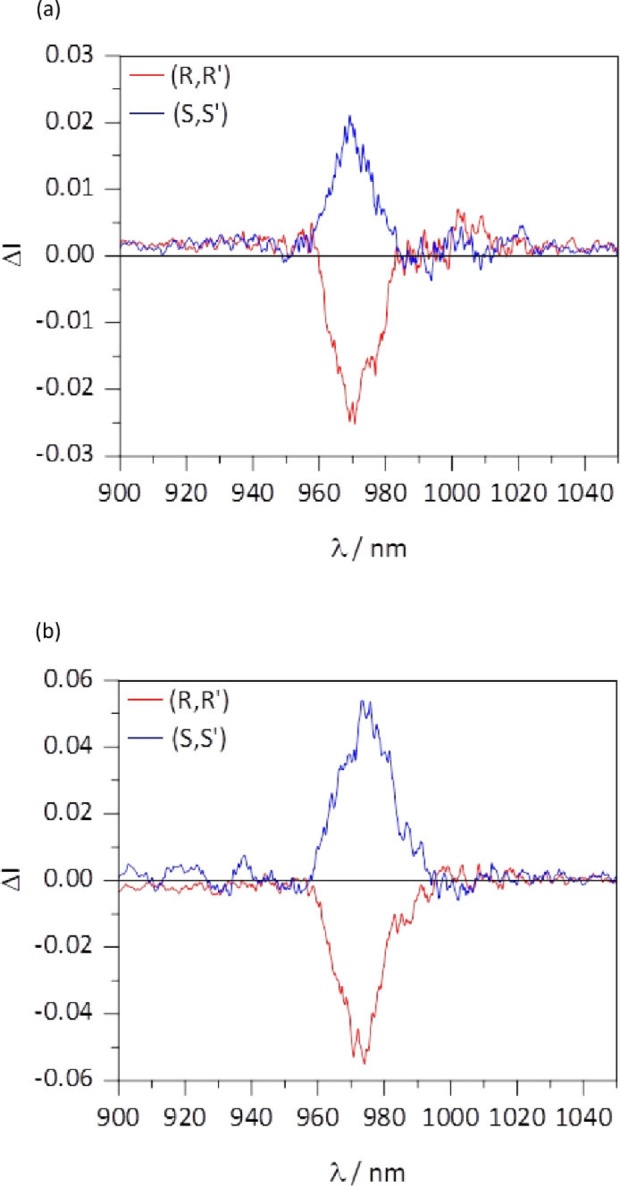
NIR CPL spectra of S,S (blue line) and R,R (red line) enantiomers of the [Yb**L**(tta)_2_] ⋅ CH_3_COO complex in DCM solution **(a)** and embedded in water stable PLGA polymer **(b)**.

The NIR CPL spectra of the two enantiomers measured in DCM show monosignated mirror signals (Figure 8a) in correspondence of the zero line of the ^2^F_5/2_→^2^F_7/2_ transition at 969 nm. The calculated *g*_lum_ factor (2(I_L_‐I_R_)/(I_L_+I_R_)) is −0.031 and +0.024 for *R,R* and *S,S* enantiomers, respectively. These values are similar or even slightly higher than those reported for Yb(tta)/PyBox based complexes.^[5d]^

Interestingly, the two enantiomers of [YbL(tta)_2_] ⋅ CH_3_COO embedded in the NPs show again a NIR CPL mirror image (Figure 8b). In fact, these spectra are almost superimposable to those recorded in DCM solutions (Figure 8a), but, surprisingly, their g_lum_ dissymmetry factor is almost double (−0.054 and +0.048 for *R,R* and *S,S* enantiomers, respectively). In the case of the Yb(III)‐based complex, and contrary to the Eu(III) counterpart, the effect of the PLGA moiety on the environment of the metal ion is such that it improves the CPL efficiency of the complex.

Although the reasons behind this increase are not clear, we can hypothesize that the difference in medium polarity in the two cases affects the ligand field parameters and mainly the angle θ between the electric (**μ**) and magnetic (**m**) transition dipole moments ($g_{lum}\approx 4\frac{\left|m\right|}{\left|\mu \right|}\cos\theta$ ), resulting in a significant change in the g‐factor.^[15]^ To further complicate the picture, we should note that the single emergent CPL band is actually a convolution of at least 4 transitions (in a C_1_ symmetry).

**Figure 9:**
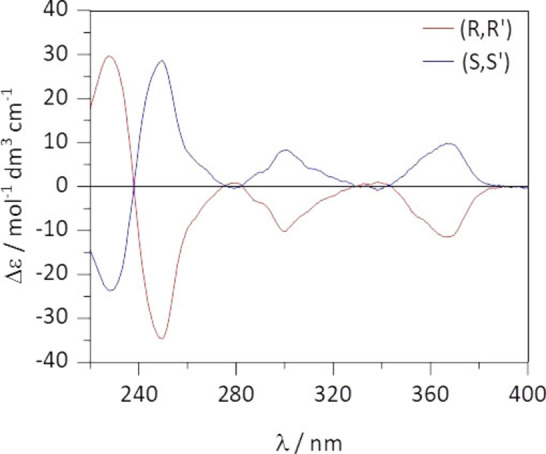
ECD of the two complex enantiomers, recorded in 1 mM solutions of DCM at room temperature.

To complete the investigation, ECD (electronic circular dichroism) studies in the UV and NIR regions were undertaken on the complex in DCM solutions (see Figure S5 for the UV absorption spectrum). ECD shows a significant band associated with the tta π‐π* transition around 350 nm, confirming that the DACH‐based ligand is effective in determining an ordered stereochemical arrangement of the diketonates around the Yb centre (Figure 9).

**Figure 10:**
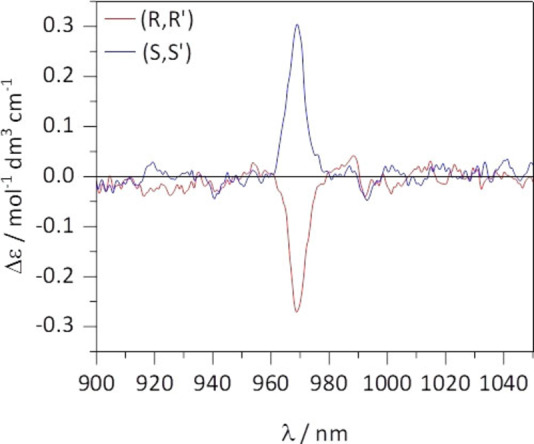
NIR‐CD spectra of the complex enantiomers, acquired in 10 mM solutions of DCM at room temperature.

Moreover, the complexes show a main monosignated NIR‐CD band around 969 nm, associated to Yb(III) ^2^F_7/2_→^2^F_5/2_ transition, similar to that observed in CPL (Figure 10). The net predominance of a positive (negative) band for the S,S’ (R,R’) complex in the NIR‐CD spectrum indicates that observed chiroptical CPL is enhanced by the coupling between the f‐f and ligand transitions (dynamic coupling).^[17]^

## Conclusion

In conclusion, we have demonstrated that the good emission properties of Yb(III)‐based hydrophobic complexes can be preserved also in water upon encapsulation of the emitting complex into PLGA nanoparticles. Such a supramolecular architecture is capable of: i) ensuring water solubility and stability to the Yb(III) complex, which normally is neither soluble nor stable in water; ii) ensuring biocompatibility to the system and iii) protecting the metal ion from the intrusion of water molecules, which are detrimental to the Ln(III) luminescence efficiency. It should also be noted that the CPL activity of the complexes in the NIR domain region is not only retained but significantly increased. This result confirms what we recently discovered on the unexpected role of achiral entities such as counterion, solvent molecule and, in the present case, the PLGA polymer surrounding the [Yb**L**(tta)_2_]⋅CH_3_COO molecule, on the CPL activity of Ln(III)‐based complexes. Despite several studies focused on CPL from nanoparticles and nanoassemblies of a different nature (pure organic and inorganic or hybrid organic–inorganic nanocomposites)^[18]^ this is, to the best of our knowledge, the first example of a water colloidal suspension containing a Yb(III) complex capable of efficiently emitting a sizeable degree of circularly polarized luminescence in the NIR region. Thanks to its features, this new material paves the way to new systems that can be considered for future applications, such as NIR‐CPL (bio)‐assays or trackers for CPL microscopy.

## Experimental Section

Yb(CH_3_COO)_3_ (Aldrich, 98 %) was stored under vacuum for several days at 80 °C and then transferred in a glove box. 2‐Thenoyltrifluoroacetyl‐acetone (htta, Alfa aesar, 98 %). PLGA (poly[DL‐lactide‐co‐glycolide], 75 : 25 lactide‐glycolide ratio, molecular weight 4,000‐15,000 CAS 26780‐50‐7), ethanol (≥99 % purity, CAS 64‐17‐5), Glycine (CAS 56‐40‐6) and Dichloromethane (DCM) (CAS 75‐09‐2) were purchased from Sigma Aldrich.

**Synthesis of the chiral complexes (*R,R*)‐ and (*S,S*) [YbL(tta)_2_]CH_3_COO (L= N, N’‐bis(2‐pyridylmethylidene)‐1,2 (*R,R*+*S,S*)‐cyclohexanediamine)**: At room temperature, 289 mg (684.9 mmol) of Htta (2‐Thenoyltrifluoroacetyl‐acetone) have been dissolved in a methanol (3 mL) solution containing 84.7 mg (1506.8 mmol) of KOH. The clear solution was slowly added to a methanol solution (7 mL) of the enantiopure ligand **L** [200.4 mg (684.9 mmol)] and Yb(CH_3_COO)_3_ [289.2 mg (684.9 mmol)]. The final mixture was stirred for 30 minutes at room temperature and then the solvent was removed under reduced pressure. The desired product was obtained in a good yield as yellowish powder upon extraction in dichloromethane (5 mL) followed by solvent removal *in vacuo*.

[Yb**L**(tta)_2_]⋅CH_3_COO: Yield ca. 80 % for both enantiomers.

Elemental Anal. Calc. for C_36_H_31_F_6_N_4_O_6_S_2_Yb (MW 966.8): C, 44.72; H, 3.23; N, 5.79; S, 6.63. Found (isomer *R,R*): C, 44.15; H, 3.70; N, 5.69; S, 6.20; (isomer *S,S*): C, 45.25 ; H, 3.79; N, 5.89; S, 7.01. In dichloromethane: ϵ (270 nm)=19300 M^−1^ cm^−1^(pyridine ring absorption); ϵ (340 nm) 28159 M^−1^ cm^−1^ (tta absorption). ESI‐MS(Scan ES+; *m/z*): 907.08 (100 %), 906.08 (85 %), 905.08 (58 %) ([[Yb**L**(tta)_2_]^+^); 804.13(100 %), 805.13 (90 %), 803.13 (55 %) ([Yb**L**(tta)(CH_3_COO)_2_+H]^+^); 315.19 (100 %) ([**L**+Na]^+^ (Scan ES‐; *m/z*): 220.97 (100 %) [tta]^−^

**Preparation of [YbL(tta)_2_]⋅CH_3_COO loaded PLGA nanoparticles**: 2 mg of PLGA and 1 mg of the complex were mixed in acetonitrile. Then, the solution was diluted 60 times with an aqueous solution of glycine (pH 9) and the precipitation of a colloidal substance (the nanoparticles) was observed. The nanoparticles were washed twice with a small amount of water, centrifuged for 30–40 minutes (11000 rpm) and then resuspended in 1 mL of water. This colloidal solution is used for the spectroscopic measurements.

## Conflict of interest

The authors declare no conflict of interest.

1

## Supporting information

As a service to our authors and readers, this journal provides supporting information supplied by the authors. Such materials are peer reviewed and may be re‐organized for online delivery, but are not copy‐edited or typeset. Technical support issues arising from supporting information (other than missing files) should be addressed to the authors.

Supporting Information

## Data Availability

Research data are not shared.
