# Supplementary material for: Near Infared Circularly Polarized Luminescence From Water Stable Organic Nanoparticles Containing a Chiral Yb(III) Complex
Source: Chemistry. 2022 May 23;28(37):e202200574. doi: 10.1002/chem.202200574 (PMC9322261; doi:10.1002/chem.202200574)

Chemistry–A European Journal

Supporting Information

Near Infared Circularly Polarized Luminescence From Water Stable Organic Nanoparticles Containing a Chiral Yb(III) Complex

Enrico Cavalli, Chiara Nardon, Oliver G. Willis, Francesco Zinna, Lorenzo Di Bari,*
Silvia Mizzoni, Silvia Ruggieri, Salvatore C. Gaglio, Massimiliano Perduca, Claudio Zaccone,
Alessandro Romeo, and Fabio Piccinelli*

Content

1. Synthetic methods
1.1. Synthesis of compound **2**
1.2. Synthesis of compounds **FA1-FA6**
1.3. Synthesis of compound **10**
1.4. Synthesis of compound **11**
1.5. Synthesis of compound **12**
1.6. Synthesis of compound **13**
1.7. Synthesis of compound **14**
1.8. Synthesis of compound **15**
1.9. Synthesis of compound ***ββ*-DLAM 909**
1.10. Synthesis of compound **19**
1.11. Synthesis of compound **20**
1.12. Synthesis of compound **21**
1.13. Synthesis of compound **22**
1.14. Synthesis of compound **23**
1.15. Synthesis of compound ***ββ*-DLAM 919**
1.16. Synthesis of compound **25**
1.17. Synthesis of compound **26**
1.18. Synthesis of compound **27**
1.19. Synthesis of compound ***ββ*-DLAM 933**
2. NMR Spectra

Synthetic methods

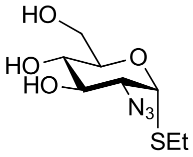

Ethyl 2-Azido-2-deoxy-1-thio-α-D-glucopyranoside (2)

To a stirred solution of **1**[1] (7.50 g, 20.0 mmol) in dry methanol (150 mL) potassium carbonate (248 mg, 1.8 mmol) was added under atmosphere of Ar. The mixture was stirred for 1 h and then made neutral by addition of Dowex 50 (H^+) ion exchange resin. The resin was filtered off, the solvent was removed to afford **2** as a white solid (4.98 g, 99%).

R_f = 0.26 (ethyl acetate); $[\alpha]_D^{20}$ = +233 (c 1.0, MeOH); ^1H NMR (600 MHz, MeOD): δ 5.41 (d, 1H, $^3J_{1,2}$ = 5.5 Hz, H-1), 3.94 (ddd, 1H, $^3J_{4,5}$ = 9.9 Hz, $^3J_{5,6a}$ = 5.4 Hz, $^3J_{5,6b}$ = 2.3 Hz, H-5), 3.80 (dd, 1H, $^2J_{6a,6b}$ = 11.7 Hz, $^3J_{5,6b}$ = 2.5 Hz, H-6b), 3.71 (dd, 1H, $^2J_{6a,6b}$ = 12.0 Hz, $^3J_{5,6a}$ = 5.4 Hz, H-6a), 3.66 (dd, 1H, $^3J_{2,3}$ = 10.3 Hz, $^3J_{1,2}$ = 5.3 Hz, H-2), 3.61 (dd, 1H, $^3J_{2,3}$ = 10.3 Hz, $^3J_{3,4}$ = 8.5 Hz, H-3), 3.35 (dd, 1H, $^3J_{4,5}$ = 9.9 Hz, $^3J_{3,4}$ = 8.6 Hz, H-4), 2.69-2.56 (m, 2H, SCH_2CH_3), 1.29 (dd, 3H, ^3J = ^3J = 7.6 Hz, SCH_2CH_3);

^{13}C NMR (151 MHz, MeOD): δ 84.49 (CH, C-1), 74.54 (CH, C-3), 74.23 (CH, C-5), 72.12 (CH, C-4), 65.25 (CH, C-2), 62.43 (CH_2, C-6), 25.18 (CH_2, SCH_2CH_3), 15.15 (CH_3, SCH_2CH_3);

HRMS (ESI) *m/z* calcd. for $[M-H]^-$ $C_8H_{14}N_3O_4S$ 248.0711, found 248.0712.

Synthesis of compounds FA1-FA6

(*R*)-3-(benzyloxy)alkanoic acids **FA1 – FA3** and (*R*)-3-(alkanoyloxy)alkanoic acids **FA4 – FA6** were prepared according to reported procedures [2]

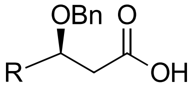

FA 1 R = C_7H_{15}
FA 2 R = C_9H_{19}
FA 3 R = $C_{11}H_{23}$

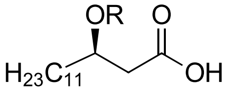

FA 4 R = $C(O)C_9H_{19}$
FA 5 R = $C(O)C_{11}H_{23}$
FA 6 R = $C(O)C_{13}H_{27}$

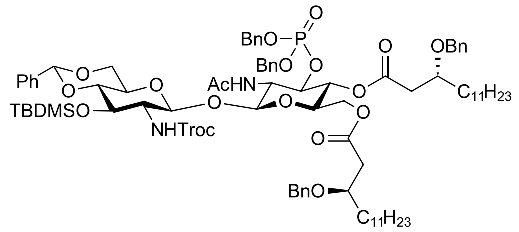

4,6-*O*-Benzylidene-3-*O*-*tert*-butyldimethylsilyl-2-deoxy-2-(2,2,2-trichloroethoxycarbonylamino)-β-D-glucopyranosyl-(1↔1)-2-acetamido-4,6-*di*-*O*-[(*R*)-3-(benzyloxy)tetradecanoyl]-3-*O*-[bis(benzyloxy)phosphoryl]-2-deoxy-β-D-glucopyranoside (10)

To a stirred solution of **9** (188 mg, 0.184 mmol) in dry DCM (3 mL) (*R*)-3-(benzyloxy)tetradecanoic acid **FA3** (3 eq, 49 mg, 0.175 mmol), DIC (1 eq., 55 mg, 68 µl 0.44 mmol) and DMAP (0.2 eq., 0.04 mmol) were successively added under atmosphere of Ar. The stirring was continued for 4 h and the second portion of reagents [**FA3** (3 eq, 49 mg, 0.175 mmol), DIC (1 eq., 55 mg, 68 µl 0,44 mmol) and DMAP (0.2 eq., 0.04 mmol)] was added under atmosphere of Ar. The reaction mixture was stirred for 2 h at r.t., diluted with DCM (100 mL), and washed with water (20 mL), 0,25 M citric acid (20 mL), sat. aq. $NaHCO_3$ (2 x 20 mL) and brine (20 mL). The organic layer was dried over Na_2SO_4, filtered and concentrated. The residue was purified by column chromatography on silica gel (toluene – EtOAc, 3:1 → 2:1, v/v) and by size-exclusion chromatography on Bio-Beads S-X1 support (toluene – DCM, 3:1) to give **10** (260 mg, 85%).

R_f = 0.7 (toluene - EtOAc, 1:1); ^{1}H NMR (600 MHz, $CDCl_3$) δ 7.46-7.20 (m, 25H, Ph), 5.80 (d, 1H, $^3J_{NH,2}$ = 8.4 Hz, NH), 5.48 (s, 1H, OC*H*Ph), 5.31 (d, 1H, $^3J_{NH',2'}$ = 8.2 Hz, NH′), 5.09 (dd, 1H, $^3J_{3,4}$ = $^3J_{4,5}$ = 9.5 Hz, H-4), 4.95 (d, 1H, $^3J_{1',2'}$ = 8.2 Hz, H-1′), 4.93-4.86 (m, 4H, OP(O)(OC*H₂*Ph)₂), 4.81 (d, 1H, $^3J_{1,2}$ = 8.4 Hz, H-1), 4.80-4.75 (m, 1H, H-3), 4.72 (d, 1H, ^2J = 12.0 Hz, CH_{2a}, Troc), 4.65 (d, 1H, ^2J = 12.0 Hz, CH_{2b}, Troc), 4.55 (d, 1H, ^2J = 11.4 Hz, OCH_2Ph), 4.52 (d, 1H, ^2J = 11.3 Hz, OCH_2Ph), 4.41 (d, 1H, ^2J = 11.5 Hz, OCH_2Ph), 4.35 (d, 1H, ^2J = 11.5 Hz, OCH_2Ph), 4.29 (dd, 1H, $^2J_{6a',6b'}$ = 10.4 Hz, $^3J_{5',6a'}$ = 4.8 Hz, H-6a′), 4.16-4.09 (m, 3H, 3′, 6ab), 3.90 (m, 1H, $β^{Myr}$-CH), 3.80-3.75 (m, 1H, NH), 3.73-3.67 (m, 2H, H-6b′, $β^{Myr}$-CH), 3.58-3.55 (m, 1H, H-5′), 3.43 (dd, 1H, $^3J_{3',4'}$ = $^3J_{4',5'}$ = 9.0 Hz, H-4′), 3.39-3.35 (m, 1H, H-5′), 3.22-3.20 (m, 1H, H-2′), 2.64 (dd, 1H, ^2J = 15.5 Hz, ^3J = 7.7 Hz, $α^{Myr}$-CH_2), 2.52 (dd, 1H, ^2J = 15.8 Hz, ^3J = 5.0 Hz, $α^{Myr}$-CH_2), 2.40 (dd, 1H, ^2J = 15.8 Hz, ^3J = 6.5 Hz, $α^{Myr}$-CH_2), 2.34 (dd, 1H, ^2J = 15.8 Hz, ^3J = 5.5 Hz, $α^{Myr}$-CH_2), 1.85 (s, 3H, NH*Ac*), 1.60-1.22 (m, 40H, CH_2), 0.89-0.86 (m, 6H, 2 $ω^{Myr}$-CH_3), 0.80 (s, 9H, $Si(CH_3)_2C(CH_3)_3$), 0.04 (s, 3H, $Si(CH_3)_2C(CH_3)_3$), -0.04 (s, 3H, $Si(CH_3)_2C(CH_3)_3$);

^{13}C NMR (151 MHz, $CDCl_3$): δ 171.39 (C=O, Myr), 170.89 (C=O, NH*Ac*), 170.25 (C=O, Myr), 153.99 (C=O, Troc), 138.68 (C_q, Ph), 138.52 (C_q, Ph), 137.09 (C_q, Ph), 135.43 (C_q, d, $^3J_{C,P}$ = 7.8 Hz, Ph), 135.39 (C_q, d, $^3J_{C,P}$ = 7.9 Hz, Ph), 129.03, 128.64, 128.57, 128.38, 128.20, 128.11, 128.04, 127.80, 127.55, 127.39, 126.24 (CH, Ph), 101.79 (CH, benzylidene acetal), 97.28, 97.24 (CH, C-1, C-1′), 95.41 (C_q, Troc), 82.09 (CH, C-4′), 76.86 (CH, d, $^2J_{C,P}$ = 6.6 Hz, C-3), 76.09 ($β^{Myr}$-*C*H), 75.33 ($β^{Myr}$-*C*H), 74.63 (CH_2, Troc), 72.04 (CH, C-5), 71.57 (CH_2, OCH_2Ph), 71.09 (CH, C-3′), 70.95 (CH_2, OCH_2Ph), 69.93 (CH_2, d, $^2J_{C,P}$ = 6.4 Hz, OP(O)(OCH_2Ph)₂), 69.34 (CH_2, d, $^2J_{C,P}$ = 6.9 Hz, OP(O)(OCH_2Ph)₂), 69.02 (CH, $^3J_{C,P}$ = 4.6 Hz, C-4), 68.58 (CH_2, C-6′), 66.05 (CH, C-5′), 62.17 (CH_2, C-6), 59.47 (CH, C-2′), 55.54 (CH, C-2), 39.58, 38.94 ($α^{Myr}$-CH_2), 34.41, 33.99, 31.91, 29.72, 29.67, 29.64, 29.34, (CH_2,

Myr), 26.51 (CH_3, $Si(CH_3)_2C(CH_3)_3$), 25.89 (CH_3, $Si(CH_3)_2C(CH_3)_3$), 25.23, 25.05 (CH_2, Myr), 23.41 (CH_3, NH*Ac*), 22.67 (CH_2, Myr), 18.10 (C_q, $Si(CH_3)_2C(CH_3)_3$), 14.24 (ω^{Myr}-CH_3), -4.24, -5.01 (CH_3, $Si(CH_3)_2C(CH_3)_3$);

^{31}P NMR (243 MHz, $CDCl_3$): δ -1.03; HRMS (ESI) *m/z* calcd. for $[M+H]^+$ $C_{86}H_{122}Cl_3N_2O_{19}PSi$ 1651.7287, found 1651.7309.

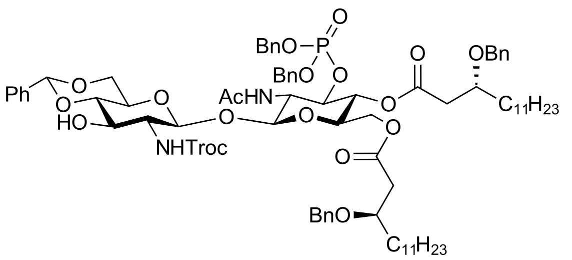

4,6-*O*-Benzylidene-2-deoxy-2-(2,2,2-trichloroethoxycarbonylamino)-β-D-glucopyranosyl-(1↔1)-2-acetamido-4,6-*di-O*-[(*R*)-3-(benzyloxy)tetradecanoyl]-3-*O*-[bis(benzyloxy)phosphoryl]-2-deoxy-β-D-glucopyranoside (11)

To a stirred solution of **10** (137 mg, 0.083 mmol) in dry THF (1 mL) in a PTFE flask a solution of $Et_3N·3HF$ (TREAT-HF, 1.5 mL) was added over a period of 30 min at 0° C. The reaction mixture was stirred for 16 h at rt, diluted with DCM (100 mL), and washed with satd. aq. $NaHCO_3$ (2 x 20 mL) and brine (20 mL). The organic layer was dried over Na_2SO_4, filtered and concentrated. The residue was purified by column chromatography on silica gel (toluene – EtOAc, 10:1 → 1:10, v/v) and by size exclusion chromatography on Bio-Beads S-X1 support (toluene – MeOH, 50:1 → 20:1, v/v) to give **11** (105 mg, 82%).

R_f = 0.3 (toluene – EtOAc, 2:3); ^{1}H NMR (600 MHz, $CDCl_3$) δ 7.48-7.20 (m, 25H, Ph), 5.95 (d, 1H, $^3J_{NH,2}$ = 8.4 Hz, NH), 5.79 (br.s , 1H, NH´), 5.52 (s, 1H, OC*H*Ph), 5.08 (dd, 1H, $^3J_{3,4}$ = $^3J_{4,5}$ = 9.6 Hz, H-4), 4.95-4.85 (m, 4H, OP(O)(OCH_2Ph)$_2$), 4.83 (d, 1H, $^3J_{1,2}$ = 8.3 Hz, H-1), 4.79-4.69 (m, 4H, H-1´, H-3, CH_2 Troc), 4.53 (s, 1H, OCH_2Ph), 4.42 (d, 1H, ^2J = 11.6 Hz, OCH_2Ph), 4.36 (d, 1H, ^2J = 11.6 Hz, OCH_2Ph), 4.30 (dd, 1H, $^2J_{6a´,6b´}$ = 10.4 Hz, $^3J_{5´,6a´}$ 5.0 Hz, H-6a´), 4.16-4.14 (m, 2H, H-6), 4.04-4.01 (m, 1H, H-3´), 3.88-3.87 (m, 1H, β^{Myr}-CH), 3.76-3.70 (m, 3H, H-2, H-6b´, β^{Myr}-CH), 3.58-5.51 (m, 1H, H-5), 3.53 (dd, 1H, $^3J_{3´,4´}$ = $^3J_{4´,5´}$ = 9.2 Hz, H-4´), 3.40-5.36 (m, 2H,H-2´, H-5´), 2.61 (dd, 1H, ^2J = 15.5 Hz, ^3J = 7.7 Hz, α^{Myr}-CH_2), 2.52 (dd, 1H, ^2J = 15.5 Hz, ^3J = 4.7 Hz, α^{Myr}-CH_2), 2.40 (dd, 1H, ^2J = 15.8 Hz, ^3J = 6.5 Hz, α^{Myr}-CH_2), 2.35 (dd, 1H, ^2J = 15.8 Hz, ^3J = 5.5 Hz, α^{Myr}-CH_2), 1.85 (s, 3H, NH*Ac*), 1.64-1.22 (m, 40H, CH_2), 0.89-0.86 (m, 6H, 2 ω^{Myr}-CH_3);

^{13}C NMR (151 MHz, $CDCl_3$): δ 171.30 (C=O, Myr), 171.04 (C=O, NH*Ac*), 170.25 (C=O, Myr), 155.17 (C=O, Troc), 138.63 (C_q, Ph), 138.52 (C_q, Ph), 136.94 (C_q, Ph), 135.43 (C_q, d, $^3J_{C,P}$ = 7.8 Hz, Ph), 135.39 (C_q, d, $^3J_{C,P}$ = 7.9 Hz, Ph), 129.21, 128.66, 128.59, 128.43, 128.27, 128.20, 128.11, 128.03, 127.90, 127.81, 127.64, 127.54, 127.39, 126.28 (CH, Ph), 101.85 (CH, benzylidene acetal), 97.67 (CH, C-1´), 97.26 (CH, C-1), 95.51 (C_q, Troc), 81.10 (CH, C-4′), 76.68 (CH, d, $^2J_{C,P}$ = 6.6 Hz, C-3), 76.27 (β^{Myr}-*C*H), 75.34 (β^{Myr}-*C*H), 74.72 (CH_2, Troc), 72.05 (CH, C-5), 71.57 (CH_2, O*C*H_2Ph), 71.59 (CH, C-3´), 71.15 (CH_2, O*C*H_2Ph), 69.80 (CH_2, d, $^2J_{C,P}$ = 6.4 Hz, OP(O)(O*C*H_2Ph)$_2$), 69.67 (CH_2, d, $^2J_{C,P}$ = 6.9 Hz, OP(O)(O*C*H_2Ph)$_2$), 68.45 (CH, $^3J_{C,P}$ = 4.6 Hz, C-4), 68.45 (CH_2, C-6′), 66.36 (CH, C-5´), 62.12 (CH_2, C-6), 58.55 (CH, C-2´), 55.57 (CH, C-2), 39.47, 38.95 (α^{Myr}-*C*H_2), 34.27, 33.95, 31.91, 31.91, 29.71,

29.67, 29.63, 29.34, 29.33, 25.23, 25.05 (CH_2, Myr), 23.39 (CH_3, NH*Ac*), 22.66 (CH_2, Myr), 14.08 (ω^{Myr}-CH_3);

^{31}P NMR (243 MHz, $CDCl_3$): δ -1.01; HRMS (ESI) *m/z* calcd. for $[M+H]^+$ $C_{80}H_{108}Cl_3N_2O_{19}P$ 1537.6422, found 1537.6432.

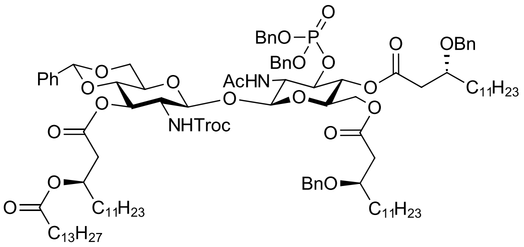

4,6-*O*-Benzylidene-2-deoxy-3-*O*-[(*R*)-3-(tetradecanoyloxy)tetradecanoyl]-2-(2,2,2-trichloroethoxycarbonylamino)-β-D-glucopyranosyl-(1↔1)-2-acetamido-4,6-*di*-*O*-[(*R*)-3-(benzyloxy)tetradecanoyl]-3-*O*-[bis(benzyloxy)phosphoryl]-2-deoxy-β-D-glucopyranoside (12)

To a stirred solution of **11** (100 mg, 0.065 mmol) in dry DCM (2 mL) (*R*)-3-(tetradecanoyloxy)tetradecanoic acid **FA6** (1,4 eq., 41 mg, 0,09 mmol), DCC (0,5 eq., 2 mg, 0.01 mmol) and DMAP (0.1 eq., 0.9 mg) were successively added under atmosphere of Ar. The mixture was stirred for 2 h at rt, diluted with DCM (50 mL) and washed with sat. aq. $NaHCO_3$ (2 x 20 mL) and brine (20 mL). The organic layer was dried over Na_2SO_4, filtered and concentrated. The residue was purified by column chromatography on silica gel (toluene – EtOAc, 5:1 → 3:1) to give **12** (100 mg, 78%).

R_f = 0.5 (toluene - EtOAc, 2:1); ^{1}H-NMR (600 MHz, $CDCl_3$) δ 7.43-7.19 (m, 25H, Ph), 5.73 (d, 1H, $^3J_{NH,2}$ = 8.1 Hz, NH), 5.49 (d, 1H, $^3J_{NH',2'}$ = 8.8 Hz, NH′), 5.47 (s, 1H, OC*H*Ph), 5.43 (dd, 1H, $^3J_{2',3'}$ = $^3J_{3',4'}$ = 10.1 Hz, H-3′), 5.19-5.14 (m, 1H, β^{Myr}-CH), 5.07 (dd, 1H, $^3J_{2,3}$ = $^3J_{3,4}$ = 9.6 Hz, H-4), 4.95-4.82 (m, 7H, H-1, H-1′, H-3, OP(O)(OC*H*$_2$Ph)$_2$), 4.72 (s, 2H, CH_2 Troc), 4.54 (s, 2H, OC*H*$_2$Ph), 4.42 (d, 1H, ^2J = 11.7 Hz, OC*H*$_2$Ph), 4.36 (d, 1H, ^2J = 11.5 Hz, OC*H*$_2$Ph), 4.31 (dd, 1H, $^2J_{6a',6b'}$ = 10.5 Hz, $^3J_{5',6a'}$ 4.9 Hz, H-6a′), 4.16-4.11 (m, 2H, H-6), 3.91-3.87 (m, 1H, β^{Myr}-CH), 3.74-3.70 (m, 2H, H-6b′, β^{Myr}-CH), 3.67-3.65 (m, 1H, H-2), 3.63 (dd, 1H, $^3J_{3',4'}$ = $^3J_{4',5'}$ = 9.5 Hz, H-4′), 3.60-3.56 (m, 1H, H-5), 3.55-3.50 (m, 1H, H-2′), 3.43 (ddd, 1H, $^3J_{5',6b'}$ = $^3J_{4',5'}$ = 9.7 Hz, $^3J_{5',6a'}$ = 5.0 Hz, H-5′), 2.66-2.58 (m, 2H, α^{Myr}-C*H*$_2$), 2.54-2.48 (m, 2H, α^{Myr}-C*H*$_2$), 2.42 (dd, 1H, ^2J = 15.8 Hz, ^3J = 6.5 Hz, α^{Myr}-C*H*$_2$), 2.35 (dd, 1H, ^2J = 15.8 Hz, ^3J = 5.4 Hz, α^{Myr}-C*H*$_2$), 2.16 (t, 2H, ^3J = 7.5 Hz, α^{Myr}-C*H*$_2$), 1.85 (s, 3H). 1.85 (s, 3H, NH*Ac*), 1.64-1.14 (m, 84H, C*H*$_2$), 0.89-0.87 (m, 12H, 4 ω^{Myr}-C*H*$_3$);

^{13}C NMR (151 MHz, $CDCl_3$): δ 173.28 (C=O, Myr), 171.36 (C=O, Myr), 171.04 (C=O, NH*Ac*), 170.25 (C=O, Myr), 169.48 (C=O, Myr), 154.27 (C=O, Troc), 138.75 (C_q, Ph), 138.55 (C_q, Ph), 136.80 (C_q, Ph), 135.48 (C_q, d, $^3J_{C,P}$ = 7.8 Hz, Ph), 135.45 (C_q, d, $^3J_{C,P}$ = 7.9 Hz, Ph), 129.11, 128.64, 128.58, 128.39, 128.20, 128.12, 128.04, 127.82, 127.56, 127.39, 126.15 (CH, Ph), 101.49 (CH, benzylidene acetal), 97.90 (CH, C-1′), 97.09 (CH, C-1), 95.60 (C_q, Troc), 78.69 (CH, C-4′), 76.62 (CH, d, $^2J_{C,P}$ = 6.6 Hz, C-3), 76.23 (β^{Myr}-*C*H), 75.34 (β^{Myr}-*C*H), 74.47 (CH_2, Troc), 72.05 (CH, C-5), 71.59 (CH_2, O*C*H$_2$Ph), 70.97 (CH_2, O*C*H$_2$Ph), 70.77 (CH, C-3′), 69.98 (β^{Myr}-*C*H), 69.76 (CH_2, d, $^2J_{C,P}$ = 6.4 Hz, OP(O)(O*C*H$_2$Ph)$_2$), 69.64 (CH_2, d, $^2J_{C,P}$ = 6.9 Hz, OP(O)(O*C*H$_2$Ph)$_2$), 69.06 (CH, $^3J_{C,P}$ = 4.6 Hz, C-4), 68.47 (CH_2, C-6′), 66.36 (CH,

C-5´), 62.18 (CH_2, C-6), 56.68 (CH, C-2´), 55.91 (CH, C-2), 39.55, 39.21, 38.98, 34.35 (α^{Myr}-CH_2), 34.03, 33.80, 31.92, 29.72, 29.68, 29.65, 29.63, 29.53, 29.51, 29.35, 29.32, 29.20, 29.13, 25.26, 25.06, 25.04, 24.98 (CH_2, Myr), 23.43 (CH_3, NH*Ac*), 22.67 (CH_2, Myr), 14.08 (ω^{Myr}-CH_3);

^{31}P NMR (243 MHz, $CDCl_3$): δ -1.04; HRMS (ESI) *m/z* calcd. for $[M+H]^+$ $C_{108}H_{160}Cl_3N_2O_{22}P$ 1974.0339, found 1974.0393.

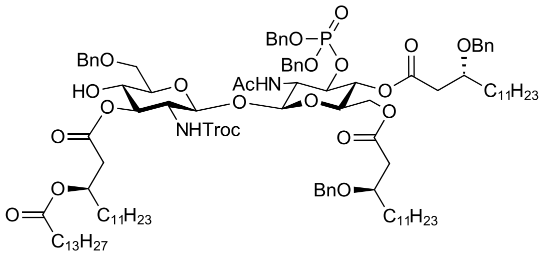

6-*O*-Benzyl-2-deoxy-3-*O*-[(*R*)-3-(tetradecanoyloxy)tetradecanoyl]-2-(2,2,2-trichloroethoxycarbonylamino)-β-D-glucopyranosyl-(1↔1)-2-acetamido-4,6-*di-O*-[(*R*)-3-(benzyloxy)tetradecanoyl]-3-*O*-[bis(benzyloxy)phosphoryl]-2-deoxy-β-D-glucopyranoside (13)

A solution of **12** (45 mg, 0.02 mmol) in dry DCM (3 mL) was stirred with powdered activated molecular sieves 4Å under atmosphere of Ar for 2h at r.t. The mixture was cooled to -78 °C and Et_3SiH (6 eq, 1.2 mmol, 22 µl) and TfOH (8 eq, 0,16 mmol, 15 µl) were added successively under atmosphere of Ar. The reaction was stirred at -78 °C for 1 h, then Et_3N (8 eq) and MeOH (0.5 mL) were added, and the mixture was stirred for 10 min. The reaction mixture was brought to r.t., diluted with DCM (50 mL), filtered over a pad of Celite and washed successively with satd. aq. $NaHCO_3$ (20 mL), water (20 mL) and brine (20 mL). The organic layer was dried over Na_2SO_4, filtered and concentrated. The residue was purified by column chromatography on silica gel (toluene – EtOAc, 2:1 → 1:2) to give **13** as white solid (38 mg, 85%).

R_f = 0.4 (toluene - EtOAc, 1:1); ^{1}H-NMR (600 MHz, CDCl3) δ 7.36-7.20 (m, 25H, Ph), 5.90 (d, 1H, $^3J_{NH,2}$ = 8.5 Hz, NH), 5.40 (d, 1H, $^3J_{NH',2'}$ = 8.5 Hz), 5.14-5.10 (m, 1H, β^{Myr}-CH), 5.09-5.06 (m, 2H, H-3´, H-4), 4.94-4.86 (m, 4H, OP(O)(OC*H*$_2$Ph)$_2$), 4.82 (d, 1H, $^3J_{1,2}$ = 8.1 Hz, H-1), 4.80-4.78 (m, 1H, H-3), 4.75 (d, 1H, $^3J_{1',2'}$ = 8.3 Hz, H-1´), 4.74-4.73 (m, 1H, CH_2a Troc), 4.65 (d, 1H, ^2J = 12.0 Hz, CH_2b Troc), 4.60 (d, 1H, ^2J = 12.0 Hz, OC*H*$_2$Ph), 4.55 (d, 1H, ^2J = 12.0 Hz, OC*H*$_2$Ph), 4.41 (d, 1H, ^2J = 11.5 Hz, OC*H*$_2$Ph), 4.35 (d, 1H, ^2J = 11.5 Hz, OC*H*$_2$Ph), 4.80-4.78 (m, 2H, OC*H*$_2$Ph), 3.90-3.86 (m, 1H, β^{Myr}-CH), 3.77 (dd, 1H, $^2J_{6a,6b}$ = 15.8 Hz, $^2J_{5,6a}$ = 7.8 Hz, H-6a), 4.80-4.78 (m, 3H, H-2, β^{Myr}-CH, H-6b), 3.63 (td, 1H, $^3J_{3,4}$ = $^3J_{4,5}$ = 9.3 Hz, $^3J_{4,OH}$ = 3.2 Hz), 3.54-3.50 (m, 2H, H-2´, H-5´), 3.49-3.46 (m, 2H, H-5), 3.36 (br.s, 1H, OH), 2.63 (dd, 1H, ^2J = 15.8 Hz, ^3J 7.8 Hz, α^{Myr}-C*H*$_2$), 2.55 (dd, 1H, ^2J = 15.1 Hz, ^3J 7.8 Hz, α^{Myr}-C*H*$_2$), 2.53-2.47 (m, 2H, α^{Myr}-C*H*$_2$), 2.41 (dd, 1H, ^2J = 15.6 Hz, ^3J 6.7 Hz, α^{Myr}-C*H*$_2$), 2.35 (dd, 1H, ^2J = 15.9, ^3J 5.4 Hz, α^{Myr}-C*H*$_2$), 2.28 (t, 2H, ^3J = 7.6 Hz, α^{Myr}-C*H*$_2$), 1.80 (s, 3H, NHAc), 1.64-1.14 (m, 84H, C*H*$_2$), 0.88 (t, 13H, ^3J = 6.9 Hz, 4 ω^{Myr}-C*H*$_3$);

^{13}C NMR (151 MHz, $CDCl_3$): δ 174.26 (C=O, Myr), 171.36 (C=O, Myr), 171.24 (C=O, Myr), 171.04 (C=O, NH*Ac*), 170.27 (C=O, Myr), 154.31 (C=O, Troc), 138.75 (C_q, Ph), 138. 61 (C_q, Ph), 137.79 (C_q, Ph), 135.55 (C_q, d, $^3J_{C,P}$ = 7.8 Hz, Ph), 135.50 (C_q, d, $^3J_{C,P}$ = 7.9 Hz, Ph),

128.58, 128.57, 128.54, 128.50, 128.37, 128.20, 128.07, 128.01, 127.88, 127.84, 127.71, 127.57, 127.54, 127.37 (CH, Ph), 97.64 (CH, C-1´), 97.06 (CH, C-1), 95.68 (C_q, Troc), 76.95 (CH, d, $^2J_{C,P}$ = 6.6 Hz, C-3), 76.21 ($β^{Myr}$-*C*H), 75.58 (CH, $^3J_{C,P}$ = 4.6 Hz, C-4), 75.35 ($β^{Myr}$-*C*H), 74.84 (CH, C-5), 74.43 (CH_2, Troc), 73.72 (CH_2, O*C*H_2Ph), 71.99 (CH, C-5´), 71.57 (CH_2, O*C*H_2Ph), 70.96 ($β^{Myr}$-*C*H), 70.96 (CH_2, O*C*H_2Ph), 69.68 (CH, C-4´), 69.65, 69.62, 69.57 (OP(O)(O*C*H_2Ph$)_2$, C-6), 69.07 (CH, C-3´), 61.81 (CH_2, C-6´), 55.79 (CH, C-2, C-2´), 40.05, 39.57, 38.96 ($α^{Myr}$-*C*H_2), 34.03 (CH_2, Myr), 34.35 ($α^{Myr}$-*C*H_2), 34.36, 34.05, 31.91, 31.89, 29.72, 29.68, 29.63, 29.61, 29.52, 29.49, 29.47, 29.35, 29.33, 29.32, 29.27, 29.25, 29.12, 25.22, 25.10, 25.07, 24.94 (CH_2, Myr), 23.31 (CH_3, NH*Ac*), 22.67 (CH_2, Myr), 14.08 ($ω^{Myr}$-*C*H_3);

^{31}P NMR (243 MHz, $CDCl_3$): δ 0.97; HRMS (ESI) *m/z* calcd. for $[M+K]^+$ $C_{108}H_{162}Cl_3N_2O_{22}P$ 2014.0054, found 2014.0014.

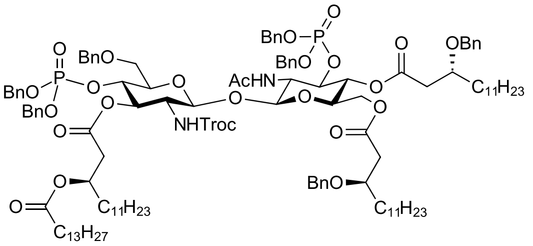

6-*O*-Benzyl-4-*O*-[bis(benzyloxy)phosphoryl]-2-deoxy-3-*O*-[(*R*)-3-(tetradecanoyloxy)-tetradecanoyl]-2-(2,2,2-trichloroethoxycarbonylamino)-β-D-glucopyranosyl-(1↔1)-2-acetamido-4,6-*di*-*O*-[(*R*)-3-(benzyloxy)tetradecanoyl]-3-*O*-[bis(benzyloxy)phosphoryl]-2-deoxy-β-D-glucopyranoside (14)

To a stirred solution of **13** (42 mg, 0.02 mmol) in dry DCM (4 mL) bis(benzyloxy)(diisopropylamino)phosphine (0.15 mmol, 50 µl) and a solution of 1*H*-tetrazol in acetonitrile (0.45 M; 100 µl) were added successively under atmosphere of Ar. The reaction mixture was stirred for 1 h, then cooled to -78 °C and a solution of mCPBA (25 mg, 0.15 mmol) in DCM (0.2 mL) was added. The reaction mixture was stirred for 1 h at -78 °C, then quenched by addition of Et_3N (100 µL) in MeOH (1 mL) and warmed to r.t. The mixture was diluted with DCM (50 mL) and washed with sat. aq. $NaHCO_3$ (20 mL), water (20 mL) and brine (20 mL). The organic layer was dried over Na_2SO_4, filtered and concentrated. The residue was purified by column chromatography on silica gel (hexane – EtOAc, 2:1, v/v) to give **14** (43 mg, 91%).

R_f = 0.4 (toluene - EtOAc, 1:1); ^{1}H NMR (600 MHz, CDCl3) δ 7.32-7.20 (m, 25H, Ph), 5.84 (1H, d, $^3J_{NH,2}$ = 8.4 Hz, NH), 5.56-5.51 (m, 2H, NH´, H-3´), 5.19-5.14 (m, 1H, $β^{Myr}$-CH), 5.09-5.04 (m, 2H, H-1´, H-4), 4.94-4.86 (m, 8H, OP(O)(OC*H*$_2$Ph$)_2$), 4.80 (1H, d, $^3J_{1,2}$ = 8.4 Hz, H-1), 4.78-4.73 (m, 1H, H-3), 4.71-4.66 (m, 2H, CH_2 Troc), 4.55 (2H, d, ^2J = 11.4 Hz, OCH_2Ph), 4.51 (2H, d, ^2J = 11.4 Hz, OCH_2Ph), 4.49-4.33 (m, 5H, H-4´, OC*H*$_2$Ph, OCH_2Ph), 4.13-4.05 (m, 2H, OCH_2Ph), 3.88-3.87 (1H, m, $β^{Myr}$-CH), 3.80-3.75 (2H, m, H-2, H-6a), 3.73-3.69 (1H, m, $β^{Myr}$-CH), 3.62-3.59 (2H, m, H-5´, H-6b´), 3.52 (1H, br.s, H-5), 3.35-3.31 (1H, m, H-2´), 2.65 (1H, dd, ^2J = 15.7 Hz, ^3J = 7.6 Hz, $α^{Myr}$-C*H*$_2$), 2.51 (1H, dd, ^2J = 15.7 Hz, ^3J = 4.9 Hz, $α^{Myr}$-C*H*$_2$), 2.45-2.32 (4H, m, $α^{Myr}$-C*H*$_2$), 2.22-2.19 (2H, m, $α^{Myr}$-C*H*$_2$), 1.77 (s, 3H, NHAc), 1.60-1.24 (m, 84H, C*H*$_2$), 0.89-0.86 (m, 12H, ^3J = 6.9 Hz, 4 $ω^{Myr}$-C*H*$_3$);

^{13}C NMR (151 MHz, $CDCl_3$): δ 173.45 (C=O, Myr), 171.34 (C=O, Myr), 170.95 (C=O, NH*Ac*), 170.26 (C=O, Myr), 169.88 (C=O, Myr), 154.07 (C=O, Troc), 138.76 (C_q, Ph), 138. 58 (C_q, Ph), 137.90 (C_q, Ph), 135.56 (C_q, d, $^3J_{C,P}$ = 7.8 Hz, Ph), 135.52 (C_q, d, $^3J_{C,P}$ = 7.9 Hz, Ph), 128.60, 128.57, 128.55, 128.43, 128.34, 128.20, 128.08, 128.04, 128.02, 127.96, 127.80, 127.74, 127.71, 127.59, 127.49, 127.37 (CH, Ph), 97.40 (CH, C-1′), 96.87 (CH, C-1), 95.54 (C_q, Troc), 77.07 (CH, d, $^2J_{C,P}$ = 6.6 Hz, C-3), 76.08 ($β^{Myr}$-*C*H), 75.34 ($β^{Myr}$-*C*H), 74.43 (CH_2, Troc), 73.99 (CH, $^3J_{C,P}$ = 4.6 Hz, C-5′), 73.86 (CH, d, $^2J_{C,P}$ = 6.6 Hz, C-4′), 73.48 (CH_2, O*C*H_2Ph), 71.98 (CH, C-3′), 71.86 (CH, C-5), 71.55 (CH_2, O*C*H_2Ph), 71.00 (CH_2, O*C*H_2Ph), 70.00 ($β^{Myr}$-*C*H), 69.68, 69.65, 69.61, 69.58 (OP(O)(O*C*H_2Ph)$_2$), 68.97 (CH, $^3J_{C,P}$ = 4.6 Hz, C-4), 68.47 (CH_2, C-6), 62.04 (CH_2, C-6′), 56.41 (CH, C-2), 55.40 (CH, C-2′), 39.55, 39.42, 38.96, 34.47 ($α^{Myr}$-*C*H_2), 34.45, 34.23, 34.09, 31.92, 29.73, 29.69, 29.66, 29.64, 29.58, 29.55, 29.53, 29.35, 29.31, 29.18, 25.24, 25.08, 25.01 (CH_2, Myr), 22.68 (CH_3, NH*Ac*), 22.68 (CH_2, Myr), 14.09 ($ω^{Myr}$-*C*H_3);

^{31}P NMR (243 MHz, $CDCl_3$): δ -1.18, -1.99; HRMS (ESI) *m/z* calcd. for $[M+H]^+$ $C_{122}H_{175}Cl_3N_2O_{25}P_2$ 2236.1098, found 2236.1105.

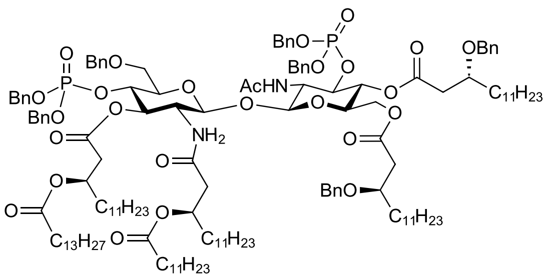

6-*O*-Benzyl-4-*O*-[bis(benzyloxy)phosphoryl]-2-deoxy-2-[(*R*)-3-(dodecanoyloxy)-tetradecanoylamino]-3-*O*-[(*R*)-3-(tetradecanoyloxy)tetradecanoyl]-β-D-glucopyranosyl-(1↔1)-2-acetamido-4,6-*di*-*O*-[(*R*)-3-(benzyloxy)tetradecanoyl]-3-*O*-[bis(benzyloxy)-phosphoryl]-2-deoxy-β-D-glucopyranoside (15)

To a stirred solution of **14** (11 mg, 5 µmol) in acetic acid (ultra-pure, 1 mL) Zn powder (10 µm, 30 mg) was added and the mixture was stirred for 3 h. The reaction mixture was diluted with DCM (10 mL) and the solids were removed by filtration over a pad of Celite. The filtrate was diluted with DCM (30 mL) and washed with sat. aq. $NaHCO_3$ (2 x 10 mL), and brine (10 mL). The organic layer was dried over Na_2SO_4, filtered over cotton and concentrated. After co-evaporation with dry toluene (3 x 5 mL) the crude residue was taken up in DMF (0.5 mL) and a solution of (*R*)-3-(dodecanoyloxy)tetradecanoic acid **FA5** (13 mg, 30 µmol) in dry $CHCl_3$ (0,5 mL) was added under stirring. Next, HATU (15 mg, 38 µmol) and DIPEA (5 µl, 38 µmol) were added successively and the reaction mixture was stirred for 6 h under atmosphere of Ar. The mixture was diluted with $CHCl_3$ (20 mL) and washed with sat. aq. $NaHCO_3$ (2 x 5 mL) and brine (10 mL), filtered over cotton and concentrated. The residue was purified by column chromatography on silica gel (toluene – EtOAc, 2:1 → 1:1, v/v) and by size-exclusion chromatography on Bio-Beads S-X1 support (toluene – DCM, 3:1) to give **15** (5 mg, 42%).

R_f = 0.3 (hexane - EtOAc, 1:1); ^{1}H NMR (600 MHz, CDCl3) δ 7.25-7.12 (m, 35H, Ph), 6.05 (1H, d, $^3J_{NH',2'}$ = 7.8 Hz, NH′), 5.87 (1H, d, $^3J_{NH,2}$ = 8.8 Hz, NH), 5.41 (1H, dd, $^3J_{2',3'}$ = $^3J_{3',4'}$ =

9.8 Hz, H-3´), 5.10-5.05 (m, 2H, $\beta^{Myr,Lau}$-CH), 5.02-5.01 (m, 1H, H-4), 5.00 (1H, d, $^3J_{1',2'}$ = 8.8 Hz, H-1´), 4.88-4.74 (m, 10H, H-1, H-3, OP(O)(OC*H*2Ph)2), 4.46 (2H, d, ^2J = 11.4 Hz, OCH_2Ph), 4.49-4.25 (m, 5H, H-4´, OC*H*2Ph, OCH_2Ph), 4.43 (2H, d, ^2J = 11.4 Hz, OCH_2Ph), 4.09 (1H, dd, $^2J_{6a,6b}$ = 12.0 Hz, $^3J_{5,6a}$ = 5.9 Hz, H-6a), 3.80-3.75 (1H, m, H-6b), 3,83-3.78 (m, 1H, β^{Myr}-CH), 3.69-3.67 (m, 1H, β^{Myr}-CH), 3.65-3.51 (m, 4H, H-2, H-5´, H-6ab), 3.47-3.42 (m, 2H, H-2´, H-5), 2.55 (1H, dd, ^2J = 15.9, ^3J Hz = 7.8 Hz, α^{Myr}-C*H*2), 2.45 (1H, dd, ^2J = 15.7, ^3J Hz = 4.9 Hz, α^{Myr}-C*H*2), 2.38-2.16 (8H, m, $\alpha^{Myr,Lau}$-C*H*2), 2.12 (2H, t, ^3J = 7.5 Hz, α^{Myr}-C*H*2), 1.68 (3H, s, NHAc), 1.39-1.17 (m, 120H, C*H*2), 0.82-0.79 (m, 18H, 5 ω^{Myr}-C*H*3, ω^{Lau}-C*H*3);

^{13}C NMR (151 MHz, $CDCl_3$): δ 173.46, 173.24, 171.33, 171.01, 170.28, 169.89 (C=O, Myr, , Lau), 138.77 (C_q, Ph), 138.59 (C_q, Ph), 137.99 (C_q, Ph), 135.6 (C_q, (OP(O)(OCH2Ph)2), 128.54, 128.40, 128.32, 128.20, 128.05, 127.99, 127.95, 127.75, 127.67, 127.60, 127.47, 127.36 (CH, Ph), 97.54 (CH, C-1´), 97.33 (CH, C-1), 77.09 (CH, C-3), 76.07, 75.34 ($\beta^{Myr,Lau}$-*C*H), 73.96 (CH, C-4´, C-5´), 73.43 (CH_2, O*C*H2Ph), 72.34 (CH, C-3´), 72.03 (CH, C-5), 72.34 (CH_2, O*C*H2Ph), 72.03 (CH_2, O*C*H2Ph), 70.50 (β^{Myr}-*C*H), 70.03 (β^{Myr}-*C*H), 69.57 (OP(O)(O*C*H2Ph)2), 69.03 (CH, $^3J_{C,P}$ = 4.6 Hz, C-4), 68.51 (CH_2, C-6´), 62.06 (CH_2, C-6), 55.79 (CH, C-2), 55.31 (CH, C-2´), 41.31, 39.60, 39.39, 38.95 (α^{Myr}-*C*H2), 34.46 (α^{Lau}-*C*H2, CH_2 Myr, CH_2 Lau), 34.22, 34.12, 31.93, 29.71, 29.67, 29.61, 29.54, 29.46, 29.37, 29.31, 29.22, 25.25, 25.19, 25.10, 25.03 (CH_2, Myr, Lau), 23.27 (CH_3, NH*Ac*), 22.27 (CH_2, Myr, Lau), 14.10 ($\omega^{Myr,\ Lau}$-*C*H3);

^{31}P NMR (243 MHz, $CDCl_3$): δ -1.27, -2.01; HRMS (ESI) *m/z* calcd. for $[M+H]^+$ $C_{145}H_{222}N_2O_{26}P_2$ 2470.5659, found 2470.5709.

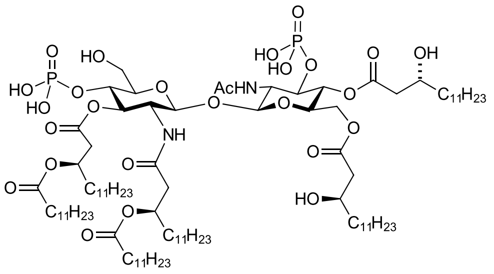

2-Deoxy-2-[(*R*)-3-(dodecanoyloxy)tetradecanoylamino]-4-*O*-phosphoryl-3-*O*-[(*R*)-3-(tetradecanoyloxy)tetradecanoyl]-β-D-glucopyranosyl-(1↔1)-2-acetamido-4,6-*di*-*O*-[(*R*)-3-(hydroxy)tetradecanoyl]-3-*O*-phosphoryl-2-deoxy-β-D-glucopyranoside (*ββ*-DLAM 909)

To a stirred solution of **15** (5 mg, 2 μmol) in dry toluene - methanol (4:1, 5 mL) in a pressure reactor Pd black (10 mg) was added under stirring. The vessel was closed, purged with argon and then filled with hydrogen (8 bar). The reaction mixture was stirred for 24 h, diluted with MeOH (5 mL), filtered through a membrane filter (regenerated cellulose; 0.45 μm), and concentrated. The residue was purified by size exclusion chromatography on Bio-Beads S-X1 support (toluene - DCM - MeOH, 5:3:1) which afforded ***ββ*-DLAM 909** (3 mg, 80%).

R_f = 0.65 (chloroform - MeOH - water, 100:75:15), ^{1}H NMR (600 MHz, $CDCl_3$ / MeOD 5:3): δ 5.28 (dd, 1H, $^3J_{2',3'}$ = $^3J_{3',4'}$ = 8.9 Hz, H-3´), 5.18-5.12 (m, 2H, $\beta^{Myr,Lau}$-CH), 5.01-4.96 (m, 2H, H-1´, H-4), 4.87 (d, 1H, $^3J_{1,2}$ = 8.7 Hz, H-1), 4.59-4.58 (m, 1H, H-3), 4.26-4.18 (3H, m, H-4´ H-6), 4.05-4.01 (m, 2H, β^{Myr}-CH), 3.82 (br.s, 1H, H-6´), 3.74 (br.s, 1H, H-5), 3.66-3.62 (m, 2H, H-2, H-2´), 3.47 (m, 1H, H-5´), 2.64 (dd, 1H, ^2J = 17.5 Hz, ^3J = 6.8 Hz, α^{Myr}-C*H*2), 2.57-2.29

(11H, m, α$^{Myr, Lau}$-CH_2), 1.97 (3H, s, NHAc), 1.58-1.26 (m, 120H, CH_2), 0.80-0.87 (m, 18H, 5 ω^{Myr}-CH_3, ω^{Lau}-CH_3);

^{13}C NMR (151 MHz, $CDCl_3$ / MeOD 5:3): δ 97.43 (CH, C-1), 96.87 (CH, C-1´), 76.04 (CH, C-3), 75.59 (CH, C-5´), 72.75 (CH, C-3´), 71.84 (CH, C-5), 71.57 (CH, C-4´), 76.56 (β^{Myr,Lau}-*C*H), 70.01 (CH, C-4), 68.64 (β^{Myr}-*C*H), 68.37 (β^{Myr}-*C*H), 62.61 (CH_2, C-6), 60.41 (CH_2, C-6´), 55.05 (CH, C-2´), 54.57 (CH, C-2), 42.41, 41.04, 38.60, 38.16, 34.41 (α^{Myr,Lau}-*C*H_2), 34.22, 34.12, 31.93, 29.71, 29.67, 29.61, 29.54, 29.46, 29.37, 29.31, 29.22, 25.25, 25.19, 25.10, 25.03 (CH_2, Myr, Lau), 23.27 (CH_3, NH*Ac*), 22.27 (CH_2, Myr, Lau), 14.10 (ω$^{Myr, Lau}$-*C*H_3);

^{31}P NMR (243 MHz, $CDCl_3$): δ 4.42, 3.12; MALDI-TOF MS: *m/z* calcd. for $[M-H]^-$ $C_{88}H_{163}N_2O_{26}P_2$ 1726.10, found 1725.86.

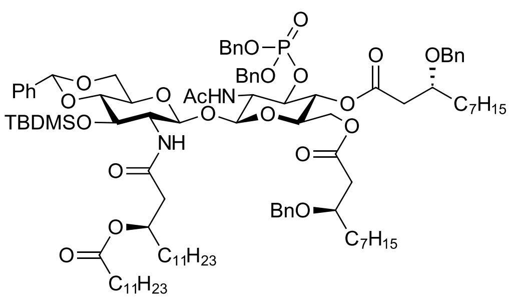

4,6-*O*-Benzylidene-3-*O*-*tert*-butyldimethylsilyl-2-deoxy-2-[(*R*)-3-(dodecanoyloxy)tetradecanoylamino]-β-D-glucopyranosyl-(1↔1)-2-acetamido-4,6-*di*-*O*-[(*R*)-3-(benzyloxy)decanoyl]-3-*O*-[bis(benzyloxy)phosphoryl]-2-deoxy-β-D-glucopyranoside (19)

To a stirred solution of **18** (73 mg, 0.058 mmol) in dry DCM (3 mL) (*R*)-3-(benzyloxy)decanoic acid **FA1** (3 eq, 49 mg, 0.175 mmol), DIC (1 eq., 7.3 mg, 0.058 mmol) and DMAP (0.1 eq., 0.006 mmol) were successively added under atmosphere of Ar. The stirring was continued for 20 min and DIC (5 eq., 36 mg, 0.3 mmol) was added in 5 equal portions over a period of 1 h. The reaction mixture was stirred for 2 h at r.t., diluted with DCM (50 mL), and washed with sat. aq. $NaHCO_3$ (20 mL) and brine (20 mL). The organic layer was dried over Na_2SO_4, filtered and concentrated. The residue was purified by HPLC (toluene – EtOAc, 5:1 → 1:1, v/v) to give **19** (86 mg, 83%).

R_f = 0.37 (toluene - EtOAc, 1:1); $[\alpha]_D^{20}$ = -13 (c 1.0, $CHCl_3$); ^{1}H NMR (600 MHz, $CDCl_3$): δ 7.48-7.42 (m, 2H, Ph), 7.37-7.18 (m, 23H, Ph), 5.88 (d, 1H, $^3J_{NH',2'}$ = 7.9 Hz, N*H*′), 5.78 (d, 1H, $^3J_{NH,2}$ = 8.6 Hz, N*H*), 5.46 (s, 1H, OC*H*Ph), 5.18 (m, 1H, β^{Myr}-C*H*), 5.13-5.05 (m, 2H, H-4, H-1′), 4.97-4.85 (m, 4H, OP(O)(OCH_2Ph)$_2$), 4.84-4.75 (m, 2H, H-1, H-3), 4.56-4.50 (m, 2H, OCH_2Ph), 4.41 (d, 1H, $^2J_{A,B}$ = 11.3 Hz, OCH_2Ph), 4.37-4.26 (m, 3H, H-3′, H-6b′, OCH_2Ph), 4.17 (dd, 1H, $^2J_{6a,6b}$ = 12.3 Hz, $^3J_{5,6b}$ = 5.6 Hz, H-6b), 4.09 (dd, 1H, $^2J_{6a,6b}$ = 12.1 Hz, $^3J_{5,6a}$ = 2.6 Hz, H-6a), 3.88 (m, 1H, β^{Cap}-C*H*), 3.77-3.69 (m, 2H, H-2, β^{Cap}-C*H*), 3.66 (dd, 1H, $^2J_{6a',6b'}$ = $^3J_{5',6a'}$ = 10.1 Hz, H-6a′), 3.55 (ddd, 1H, $^3J_{4,5}$ = 10.0 Hz, $^3J_{5,6b}$ = 5.6 Hz, $^3J_{5,6a}$ = 2.0 Hz, H-5), 3.44 (ddd, 1H, $^3J_{4',5'}$ = $^3J_{5',6a'}$ = 9.7 Hz, $^3J_{5',6b'}$ = 5.0 Hz, H-5′), 3.39 (dd, 1H, $^3J_{3',4'}$ = $^3J_{4',5'}$ = 9.0 Hz, H-4′), 3.22 (m, 1H, H-2′), 2.63 (dd, 1H, ^2J = 15.7 Hz, ^3J = 7.8 Hz, α^{Cap}-CH_2), 2.52 (dd, 1H, ^2J = 15.7 Hz, ^3J = 4.7 Hz, α^{Cap}-CH_2), 2.45-2.31 (m, 4H, α^{Cap}-CH_2, α^{Myr}-CH_2), 2.31-2.25 (m, 2H, α^{Lau}-CH_2), 1.84 (s, 3H, CH_3, NHAc), 1.68-1.13 (m, 62H, CH_2), 0.90-0.84 (m, 12H, ω^{Myr}-CH_3,

ω^{Lau}-CH_3, ω^{Cap}-CH_3), 0.80 (s, 9H, Si(CH$_3$)$_2$C(CH_3)$_3$), -0.03 (s, 3H, Si(CH_3)$_2$C(CH$_3$)$_3$) -0.07 (s, 3H, Si(CH_3)$_2$C(CH$_3$)$_3$);

^{13}C NMR (151 MHz, CDCl$_3$): δ 173.57 (C=O, Lau) 171.49, 170.98, 170.40, 170.27 (C=O, Myr, Cap, NHAc), 138.89, 138.70, 137.34 (C$_q$, Ph), 135.67, 135.62, 135.58 (C$_q$, Ph), 129.15, 128.77, 128.75, 128.72, 128.70, 128.50, 128.35, 128.25, 128.22, 128.16, 127.95, 127.86, 127.72, 127.64, 127.53, 126.44 (CH, Ph), 101.99 (CH, benzylidene acetal), 97.87 (CH, C-1), 97.55 (CH, C-1′), 82.52 (CH, C-4′), 77.00 (CH, C-3), 76.16, 75.48 (β^{Cap}-*C*H), 72.23 (CH, C-5), 71.59, 71.15 (CH$_2$, O*C*H$_2$Ph), 71.01 (CH, C-3′), 70.79 (β^{Myr}-*C*H), 69.84 (CH$_2$, d, $^2J_{C,P}$ = 5.8 Hz, OP(O)(O*C*H$_2$Ph)$_2$), 69.74 (CH$_2$, d, $^2J_{C,P}$ = 5.8 Hz, OP(O)(O*C*H$_2$Ph)$_2$), 69.14 (CH, d, $^3J_{C,P}$ = 3.1 Hz, C-4), 68.82 (CH$_2$, C-6′), 65.98 (CH, C-5′), 62.22 (CH$_2$, C-6), 59.30 (CH, C-2′), 55.80 (CH, C-2), 41.46, 39.71, 39.08 (α^{Myr}-*C*H$_2$, α^{Cap}-*C*H$_2$), 34.73, 34.50, 34.19, 32.07, 30.06, 32.00, 31.96, 29.86, 29.82, 29.81, 29.78, 29.73, 29.70, 29.52, 29.51, 29.49, 29.45, 29.41, 29.38 (CH$_2$, Myr, Lau), 25.88 (CH$_3$, Si(CH$_3$)$_2$C(*C*H$_3$)$_3$), 25.35, 25.21, 25.17 (CH$_2$, Myr, Lau), 23.56 (CH$_3$, NH*Ac*), 22.82, 22.79 (CH$_2$, Myr, Lau), 18.23 (C$_q$, Si(CH$_3$)$_2$*C*(CH$_3$)$_3$), 14.24 (ω^{Myr}-*C*H$_3$, ω^{Lau}-*C*H$_3$, ω^{Cap}-*C*H$_3$), -4.07, -4.69 (CH$_3$, Si(*C*H$_3$)$_2$C(CH$_3$)$_3$);

^{31}P NMR (243 MHz, CDCl$_3$) δ -0.97; HRMS (ESI) *m/z* calcd. for [M+COOH]$^-$ $C_{102}H_{154}N_2O_{22}PSi$ 1818.0506, found 1818.0526.

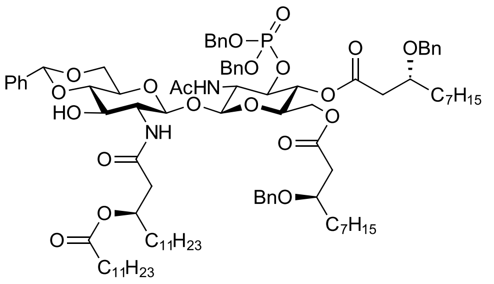

4,6-*O*-Benzylidene-2-deoxy-2-[(*R*)-3-(dodecanoyloxy)tetradecanoylamino]-β-D-glucopyranosyl-(1↔1)-2-acetamido-4,6-di-*O*-[(*R*)-3-(benzyloxy)decanoyl]-3-*O*-[bis(benzyloxy)phosphoryl]-2-deoxy-β-D-glucopyranoside (20)

To a stirred solution of **19** (58 mg, 0,03 mmol) in dry THF (2 mL) in a PTFE flask a solution of Et$_3$N·3HF (TREAT-HF, 1.4 mL) was added at 0° C in the atmosphere of Ar. The reaction mixture was stirred for 12 h at r.t., diluted with DCM (50 mL), and washed with sat. aq. NaHCO$_3$ (2 x 20 mL) and brine (20 mL). The organic layer was dried over Na$_2$SO$_4$, filtered and concentrated. The residue was purified by column chromatography on silica gel (toluene – EtOAc, 10:1 → 1:10, v/v) and by size exclusion chromatography on Bio-Beads S-X1 support (toluene – DCM, 3:1, v/v) to give **20** (52 mg, 96%).

R$_f$ = 0.29 (toluene - DCM, 3:1); $[\alpha]_D^{20}$ = -22.5 (c 1.0, CHCl$_3$); ^{1}H NMR (600 MHz, CDCl$_3$): δ 7.52-7.46 (m, 2H, Ph), 7.38-7.18 (m, 23H, Ph), 6.13 (d, 1H, $^3J_{NH',2'}$ = 5.8 Hz, N*H*′), 5.76 (d, 1H, $^3J_{NH,2}$ = 8.3 Hz, N*H*), 5.53 (s, 1H, OC*H*Ph), 5.18 (m, 1H, β^{Myr}-C*H*), 5.08 (dd, 1H, $^3J_{3,4}$ = $^3J_{4,5}$ = 9.6 Hz, H-4), 4.97-4.87 (m, 4H, OP(O)(OC*H*$_2$Ph)$_2$), 4.81 (m, 1H, H-3), 4.76 (d, 1H, $^3J_{1',2'}$ = 8.3 Hz, H-1′), 4.72 (d, 1H, $^3J_{1,2}$ = 8.3 Hz, H-1), 4.56 (d, 1H, $^3J_{OH',3'}$ = 2.6 Hz, O*H*′), 4.55-4.49 (m, 2H, OC*H*$_2$Ph), 4.43 (d, 1H, $^2J_{A,B}$ = 11.4 Hz, OC*H*$_2$Ph), 4.38 (d, 1H, $^2J_{A,B}$ = 11.5 Hz, OC*H*$_2$Ph), 4.27 (dd, 1H, $^2J_{6a',6b'}$ = 10.4 Hz, $^3J_{5',6b'}$ = 4.8 Hz, H-6b′), 4.23 (dd, 1H, $^2J_{6a,6b}$ = 12.3 Hz, $^3J_{5,6b}$ = 6.4 Hz, H-6b), 4.10 (dd, 1H, $^2J_{6a,6b}$ = 12.3 Hz, $^3J_{5,6a}$ = 2.0 Hz, H-6a), 4.05 (ddd, 1H, $^3J_{2',3'}$ = $^3J_{3',4'}$ = 9.3 Hz, $^3J_{OH',3'}$ = 2.2 Hz, H-3′), 3.88 (m, 1H, β^{Cap}-C*H*), 3.76-3.67 (m, 3H, H-2, H-6a′, β^{Cap}-C*H*), 3.56 (m, 1H, H-5), 3.53 (dd, 1H, $^3J_{3',4'}$ = $^3J_{4',5'}$ = 9.3 Hz, H-4′), 3.44 (ddd, 1H,

$^{3}J_{1',2'}$ = $^{3}J_{2',3'}$ = 9.0 Hz, $^{3}J_{NH',2'}$ = 6.2 Hz, H-2′), 3.38 (ddd, 1H, $^{3}J_{4',5'}$ = $^{3}J_{5',6a'}$ = 9.8 Hz, $^{3}J_{5',6b'}$ = 5.0 Hz, H-5′), 2.60-2.50 (m, 2H, α^{Cap}-C*H*$_2$), 2.46-2.34 (m, 4H, α^{Cap}-C*H*$_2$, α^{Myr}-C*H*$_2$), 2.31-2.25 (m, 2H, α^{Lau}-C*H*$_2$), 1.85 (s, 3H, C*H*$_3$, NHAc), 1.68-1.15 (m, 62H, C*H*$_2$), 0.91-0.82 (m, 12H, ω^{Myr}-C*H*$_3$, ω^{Lau}-C*H*$_3$, ω^{Cap}-C*H*$_3$);

^{13}C NMR (151 MHz, CDCl$_3$): δ 174.12 (C=O, Lau) 172.23 (C=O, Myr), 171.49 (C=O, Cap), 171.08 (C=O, NHAc), 170.43 (C=O, Cap), 138.73, 138.62, 137.22 (C$_q$, Ph), 135.60, 135.54, 135.49 (C$_q$, Ph), 129.23, 128.84, 128.75, 128.62, 128.38, 128.32, 128.24, 128.17, 128.02, 127.83, 127.68, 127.57, 126.53 (CH, Ph), 102.07 (CH, benzylidene acetal), 98.28 (CH, C-1′), 97.90 (CH, C-1), 81.27 (CH, C-4′), 76.71 (CH, d, $^{2}J_{C,P}$ = 5.6 Hz, C-3), 76.44, 75.51 (β^{Cap}-*C*H), 72.41 (CH, C-5), 71.87 (CH, C-3′), 71.85 (CH$_2$, O*C*H$_2$Ph), 71.44 (β^{Myr}-*C*H), 71.04 (CH$_2$, O*C*H$_2$Ph), 69.93 (CH$_2$, d, $^{2}J_{C,P}$ = 6.5 Hz, OP(O)(O*C*H$_2$Ph)$_2$), 69.81 (CH$_2$, d, $^{2}J_{C,P}$ = 5.4 Hz, OP(O)(O*C*H$_2$Ph)$_2$), 69.11 (CH, d, $^{3}J_{C,P}$ = 3.7 Hz, C-4), 68.61 (CH$_2$, C-6′), 66.70 (CH, C-5′), 62.20 (CH$_2$, C-6), 58.75 (CH, C-2′), 55.74 (CH, C-2), 42.27 (α^{Myr}-*C*H$_2$), 39.73, 39.03 (α^{Cap}-*C*H$_2$), 34.71, 34.63, 34.39, 34.06, 32.05, 31.97, 31.96, 29.81, 29.78, 29.76, 29.73, 29.72, 29.68, 29.56, 29.49, 29.45, 29.43, 29.40, 29.34, 25.43, 25.40, 25.21, 25.14 (CH$_2$, Myr, Lau), 23.54 (CH$_3$, NH*Ac*), 22.82, 22.79, 22.77 (CH$_2$, Myr, Lau), 14.24, 14.23 (ω^{Myr}-*C*H$_3$, ω^{Lau}-*C*H$_3$, ω^{Cap}-*C*H$_3$);

^{31}P NMR (243 MHz, CDCl$_3$): δ -0.96; HRMS (ESI) *m/z* calcd. for [M+H]$^+$ $C_{95}H_{140}N_2O_{20}P$ 1659.9732, found 1659.9741.

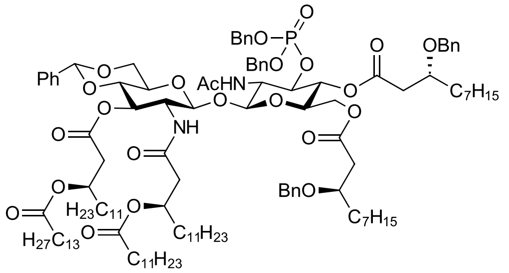

4,6-*O*-Benzylidene-2-deoxy-2-[(*R*)-3-(dodecanoyloxy)tetradecanoylamino]-3-*O*-[(*R*)-3-(tetradecanoyloxy)tetradecanoyl]-β-D-glucopyranosyl-(1↔1)-2-acetamido-4,6-di-*O*-[(*R*)-3-(benzyloxy)decanoyl]-3-*O*-[bis(benzyloxy)phosphoryl]-2-deoxy-β-D-glucopyranoside (21)

To a stirred solution of **20** (33 mg, 0.02 mmol) in dry DCM (1 mL) (*R*)-3-(tetradecanoyloxy)tetradecanoic acid **FA6** (1,5 eq., 13,5 mg, 0.03 mmol), DCC (0,5 eq., 2 mg, 0.01 mmol) and DMAP (0.05 eq., 0.12 mg, 0.001 mmol) were successively added. The stirring was continued for 15 min and DCC (2 eq., 8 mg, 0,04 mmol) and **FA6** (1,5 eq., 13,5 mg, 0,030 mmol) were added in small portions over a period of 3 h. The reaction mixture was diluted with DCM (50 mL) and washed with sat. aq. NaHCO$_3$ (2 x 20 mL) and brine (20 mL). The organic layer was dried over Na$_2$SO$_4$, filtered and concentrated. The residue was purified by column chromatography on silica gel (1. toluene – EtOAc, 2:1, v/v; 2. hexane – EtOAc, 2:3, v/v) to give **21** (31 mg, 74%).

R$_f$ = 0.45 (toluene - EtOAc, 1:1); $[\alpha]_D^{20}$ = -13.6 (c 1.2, CHCl$_3$); ^{1}H NMR (600 MHz, CDCl$_3$): δ 7.44-7.39 (m, 2H, Ph), 7.36-7.18 (m, 23H, Ph), 5.99 (d, 1H, $^{3}J_{NH',2'}$ = 8.8 Hz, N*H*′), 5.75 (d, 1H, $^{3}J_{NH,2}$ = 8.1 Hz, N*H*), 5.46 (s, 1H, OC*H*Ph), 5.34 (dd, 1H, $^{3}J_{2',3'}$ = $^{3}J_{3',4'}$ = 9.8 Hz, H-3′), 5.19-5.08 (m, 2H, β^{Myr}-C*H*), 5.05 (dd, 1H, $^{3}J_{3,4}$ = $^{3}J_{4,5}$ = 9.6 Hz, H-4), 5.02-4.86 (m, 7H, H-1, H-3, H-1′, OP(O)(OC*H*$_2$Ph)$_2$), 4.56-4.50 (m, 2H, OC*H*$_2$Ph), 4.42 (d, 1H, $^{2}J_{A,B}$ = 11.5 Hz,

OCH_2Ph), 4.35 (d, 1H, $^2J_{A,B}$ = 11.6 Hz, OCH_2Ph), 4.29 (dd, 1H, $^2J_{6a',6b'}$ = 10.4 Hz, $^3J_{5',6b'}$ = 5.0 Hz, H-6b′), 4.21 (dd, 1H, $^2J_{6a,6b}$ = 12.2 Hz, $^3J_{5,6b}$ = 6.0 Hz, H-6b), 4.06 (dd, 1H, $^2J_{6a,6b}$ = 11.9 Hz, $^3J_{5,6a}$ = 1.4 Hz, H-6a), 3.89 (m, 1H, β^{Cap}-C*H*), 3.80 (ddd, 1H, $^3J_{1',2'}$ = $^3J_{2',3'}$ = $^3J_{NH',2'}$ = 9.2 Hz, H-2′), 3.75-3.67 (m, 2H, H-6a′, β^{Cap}-C*H*), 3.62 (dd, 1H, $^3J_{3',4'}$ = $^3J_{4',5'}$ = 9.5 Hz, H-4′), 3.58 (ddd, 1H, $^3J_{4,5}$ = 9.6 Hz, $^3J_{5,6b}$ = 6.3 Hz, $^3J_{5,6a}$ = 2.1 Hz, H-5), 3.51-3.41 (m, 2H, H-2, H-5′), 2.63-2.56 (m, 2H, α^{Cap}-CH_2, α^{Myr}-CH_2), 2.55-2.47 (m, 2H, α^{Cap}-CH_2, α^{Myr}-CH_2), 2.47-2.40 (m, 2H, α^{Cap}-CH_2, α^{Myr}-CH_2), 2.37 (dd, 1H, ^2J = 15.9 Hz, ^3J = 5.4 Hz, α^{Cap}-CH_2), 2.34-2.24 (m, 3H, α^{Lau}-CH_2, α^{Myr}-CH_2), 2.16-2.10 (m, 2H, α^{Myr}-CH_2), 1.82 (s, 3H, CH_3, NHAc), 1.67-1.08 (m, 104H, CH_2), 0.92-0.83 (m, 18H, ω^{Myr}-CH_3, ω^{Lau}-CH_3, ω^{Cap}-CH_3);

^{13}C NMR (151 MHz, $CDCl_3$): δ 173.66 (C=O, Lau) 173.28 (C=O, Myr), 171.52 (C=O, Cap), 171.11 (C=O, NHAc), 170.46 (C=O, Cap), 170.26 (C=O, Myr), 169.79 (C=O, Myr), 138.83, 138.69, 137.03 (C_q, Ph), 135.71, 135.67 (C_q, Ph), 129.20, 128.77, 128.74, 128.72, 128.70, 128.53, 128.35, 128.31, 128.23, 128.16, 128.07, 127.93, 127.71, 127.52, 126.30 (CH, Ph), 101.63 (CH, benzylidene acetal), 98.39 (CH, C-1′), 97.00 (CH, C-1), 78.86 (CH, C-4′), 76.44 (CH, d, $^2J_{C,P}$ = 5.6 Hz, C-3), 76.27, 75.50 (β^{Cap}-*C*H), 72.23 (CH, C-5), 71.37 (CH, C-3′), 71.14 (CH_2, O*C*H_2Ph), 71.07 (CH_2, O*C*H_2Ph), 70.91, 70.10 (β^{Myr}-*C*H), 69.84 (CH_2, d, $^2J_{C,P}$ = 5.8 Hz, OP(O)(O*C*H_2Ph)$_2$), 69.74 (CH_2, d, $^2J_{C,P}$ = 5.6 Hz, OP(O)(O*C*H_2Ph)$_2$), 69.29 (CH, d, $^3J_{C,P}$ = 3.2 Hz, C-4), 68.66 (CH_2, C-6′), 66.54 (CH, C-5′), 62.33 (CH_2, C-6), 56.60 (CH, C-2), 54.90 (CH, C-2′), 41.63 (α^{Myr}-*C*H_2), 39.76 (α^{Cap}-*C*H_2), 39.28 (α^{Myr}-*C*H_2), 39.08 (α^{Cap}-*C*H_2), 34.65, 34.47, 34.31, 34.18, 34.01, 32.07, 31.99, 31.96, 29.88, 29.84, 29.80, 29.72, 29.68, 29.57, 29.53, 29.50, 29.48, 29.43, 29.41, 29.29, 25.41, 25.37, 25.22, 25.18, 25.12 (CH_2, Myr, Lau), 23.57 (CH_3, NH*Ac*), 22.82, 22.79 (CH_2, Myr, Lau), 14.24 (ω^{Myr}-*C*H_3, ω^{Lau}-*C*H_3, ω^{Cap}-*C*H_3);

^{31}P NMR (243 MHz, $CDCl_3$): δ -1.20; HRMS (ESI) *m/z* calcd. for $[M+COOH]^-$ $C_{124}H_{192}N_2O_{25}P$ 2140.3557, found 2140.3524.

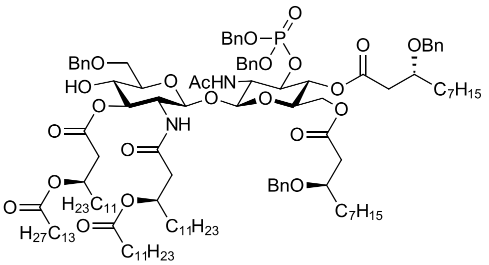

6-*O*-Benzyl-2-deoxy-2-[(*R*)-3-(dodecanoyloxy)tetradecanoylamino]-3-*O*-[(*R*)-3-(tetradecanoyloxy)tetradecanoyl]-β-D-glucopyranosyl-(1↔1)-2-acetamido-4,6-di-*O*-[(*R*)-3-(benzyloxy)decanoyl]-3-*O*-[bis(benzyloxy)phosphoryl]-2-deoxy-β-D-glucopyranoside (22)

A solution of **21** (30 mg, 0.014 mmol) in dry DCM (4 mL) was stirred with powdered activated molecular sieves (4Å, 0.3 g) under atmosphere of Ar for 2 h at r.t. The mixture was cooled to -78 °C and Et_3SiH (6 eq, 0.86 mmol, 14 µl) and TfOH (10 eq, 0.14 mmol, 12 µl) were added successively under atmosphere of Ar. The reaction was stirred at -78 °C for 1h, then Et_3N (4 eq) and MeOH (0,5 mL) were added, and the mixture was stirred for 10 min. The reaction mixture was brought to r.t., diluted with DCM (50 mL), filtered over a pad of Celite and washed successively with sat. aq. $NaHCO_3$ (20 mL), water (20 mL) and brine (20 mL). The organic layer was dried over Na_2SO_4, filtered and concentrated. The residue was purified by

column chromatography on silica gel (toluene – EtOAc, 1:1 → 2:1) to give **22** as white solid (25 mg, 88%).

R_f = 0.18 (toluene - EtOAc, 1:1); $[\alpha]_D^{20}$ = -3.7 (c 0.8, $CHCl_3$); ^1H NMR (600 MHz, $CDCl_3$): δ 7.38-7.18 (m, 25H, Ph), 5.85 (d, 1H, $^3J_{NH',2'}$ = 8.6 Hz, N*H*′), 5.75 (d, 1H, $^3J_{NH,2}$ = 8.1 Hz, N*H*), 5.16-4.85 (m, 10H, H-1, H-3, H-4, H-3′, β^{Myr}-C*H*, OP(O)(OCH_2Ph)$_2$), 4.71 (d, 1H, $^3J_{1',2'}$ = 8.1 Hz, H-1′), 4.61-4.50 (m, 4H, OCH_2Ph), 4.42 (d, 1H, $^2J_{A,B}$ = 11.8 Hz, OCH_2Ph), 4.35 (d, 1H, $^2J_{A,B}$ = 11.5 Hz, OCH_2Ph), 4.19 (dd, 1H, $^2J_{6a,6b}$ = 12.2 Hz, $^3J_{5,6b}$ = 5.6 Hz, H-6b), 4.05 (m, 1H, H-6a), 3.88 (m, 1H, β^{Cap}-C*H*), 3.83-3.68 (m, 4H, H-2′, H-6a′, H-6b′, β^{Cap}-C*H*), 3.64 (dd, 1H, $^3J_{3',4'}$ = $^3J_{4',5'}$ = 9.3 Hz, H-4′), 3.58-3.43 (m, 3H, H-2, H-5, H-5′), 3.33 (br s, 1H, O*H*′), 2.63-2.47 (m, 4H, α^{Cap}-CH_2, α^{Myr}-CH_2), 2.47-2.40 (m, 2H, α^{Cap}-CH_2, α^{Myr}-CH_2), 2.37 (dd, 1H, ^2J = 15.7 Hz, ^3J = 5.6 Hz, α^{Cap}-CH_2), 2.33-2.23 (m, 5H, α^{Lau}-CH_2, α^{Myr}-CH_2), 1.77 (s, 3H, CH_3, NHAc), 1.65-1.12 (m, 104H, CH_2), 0.94-0.80 (m, 18H, ω^{Myr}-CH_3, ω^{Lau}-CH_3, ω^{Cap}-CH_3);

^{13}C NMR (151 MHz, $CDCl_3$): δ 174.43, 173.58 (C=O, Myr, Lau), 171.53, 171.49 (C=O, Myr, Cap), 171.14 (C=O, NHAc), 170.48 (C=O, Cap), 170.12 (C=O, Myr), 138.88, 138.72, 138.03 (C_q, Ph), 135.78, 135.73 (C_q, Ph), 128.70, 128.64, 128.53, 128.37, 128.22, 128.15, 127.97, 127.83, 127.74, 127.71, 127.53 (CH, Ph), 98.12 (CH, C-1′), 96.97 (CH, C-1), 76.73 (CH, d, $^2J_{C,P}$ = 5.6 Hz, C-3), 76.30 (β^{Cap}-*C*H), 76.17 (CH, C-3′), 75.52 (β^{Cap}-*C*H), 75.02 (CH, C-5′), 73.86 (CH_2, O*C*H_2Ph), 72.17 (CH, C-5), 71.77 (CH_2, O*C*H_2Ph), 71.22 (β^{Myr}-*C*H), 71.07 (CH_2, O*C*H_2Ph), 71.00 (β^{Myr}-*C*H), 69.86 (CH, C-4′), 69.84, 69.79, 69.75, 69.72, 69.69 (CH_2, C-6′, OP(O)(O*C*H_2Ph)$_2$), 69.31 (CH, br, C-4), 62.33 (CH_2, C-6), 56.39 (CH, C-2), 53.72 (CH, C-2′), 41.67 (α^{Myr}-*C*H_2), 40.27 (α^{Myr}-*C*H_2), 39.81 (α^{Cap}-*C*H_2), 39.09 (α^{Cap}-*C*H_2), 34.89, 34.65, 34.52, 34.27, 34.22, 32.08, 32.01, 31.98, 29.89, 29.85, 29.81, 29.74, 29.70, 29.68, 29.57, 29.54, 29.51, 29.46, 29.44, 29.43, 29.41, 29.31, 25.44, 25.38, 25.29, 25.25, 25.19, 25.10 (CH_2, Myr, Lau), 23.48 (CH_3, NH*Ac*), 22.84, 22.81 (CH_2, Myr, Lau), 14.25 (ω^{Myr}-*C*H_3, ω^{Lau}-*C*H_3, ω^{Cap}-*C*H_3);

^{31}P NMR (243 MHz, $CDCl_3$): δ -1.32. HRMS (ESI) *m/z* calcd. for $[M+COOH]^-$ $C_{124}H_{194}N_2O_{25}P$ 2142.3732, found 2142.3697.

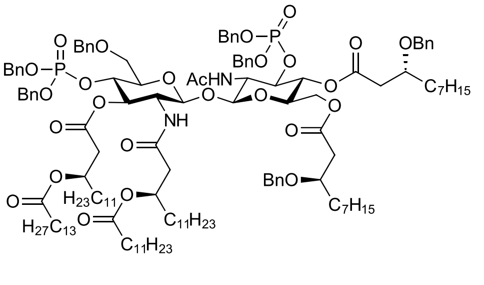

6-*O*-Benzyl-4-*O*-[bis(benzyloxy)phosphoryl]-2-deoxy-2-[(*R*)-3-(dodecanoyloxy)tetradecanoylamino]-3-*O*-[(*R*)-3-(tetradecanoyloxy)tetradecanoyl]-β-D-glucopyranosyl-(1↔1)-2-acetamido-4,6-di-*O*-[(*R*)-3-(benzyloxy)decanoyl]-3-*O*-[bis(benzyloxy)phosphoryl]-2-deoxy-β-D-glucopyranoside (23)

To a stirred solution of **22** (18 mg, 0.009 mmol) in dry DCM (4 mL) bis(benzyloxy)(diisopropylamino)phosphine (0.02 mmol, 8 μL) and a solution of 1*H*-tetrazol in acetonitrile (0.45 M; 25 μL) were added successively under atmosphere of Ar. The reaction mixture was stirred for 1 h, then cooled at -78 °C and a solution of mCPBA (0.03 mmol, 5,3 mg) was added. The reaction mixture was stirred for 1 h at -78 °C, then quenched by

addition of Et_3N (100 µL) in MeOH (1 mL) and brought to r.t. The mixture was diluted with DCM (50 mL) and washed with sat. aq. $NaHCO_3$ (20 mL), water (20 mL) and brine (20 mL). The organic layer was dried over Na_2SO_4, filtered and concentrated. The residue was purified by column chromatography on silica gel (hexane – EtOAc, 2:1, v/v) to give **23** (20 mg, 95%).

R_f = 0.21 (hexane - EtOAc, 1:1); ^{1}H NMR (600 MHz, $CDCl_3$): δ 7.37-7.17 (m, 35H, Ph), 6.07 (d, 1H, $^3J_{NH',2'}$ = 7.9 Hz, N*H*′), 5.82 (d, 1H, $^3J_{NH,2}$ = 8.1 Hz, N*H*), 5.48 (dd, 1H, $^3J_{3',4'}$ = $^3J_{2',3'}$ = 9.6 Hz, H-3′), 5.18-5.11 (m, 2H, β^{Myr}-C*H*), 5.10-5.04 (m, 2H, H-4, H-1′), 4.97-4.80 (m, 10H, H-1, H-3, OP(O)(OCH_2Ph)$_2$), 4.55-4.38 (m, 6H, H-4′, OCH_2Ph), 4.33 (d, 1H, $^2J_{A,B}$ = 11.3 Hz, OCH_2Ph), 4.17 (dd, 1H, $^2J_{6a,6b}$ = 12.2 Hz, $^3J_{5,6b}$ = 5.4 Hz, H-6b), 4.06 (m, 1H, H-6a), 3.88 (m, 1H, β^{Cap}-C*H*), 3.78-3.56 (m, 5H, H-2, H-5′, H-6a′, H-6b′, β^{Cap}-C*H*), 3.56-3.46 (m, 2H, H-5, H-2′), 2.62 (dd, 1H, ^2J = 15.8 Hz, ^3J = 7.7 Hz, α^{Cap}-CH_2), 2.52 (dd, 1H, ^2J = 15.6 Hz, ^3J = 4.6 Hz, α^{Cap}-CH_2), 2.46-2.22 (m, 8H, α^{Cap}-CH_2, α^{Myr}-CH_2, $\alpha^{Myr/Lau}$-CH_2), 2.21-2.16 (m, 2H, $\alpha^{Myr/Lau}$-CH_2) 1.75 (s, 3H, CH_3, NHAc), 1.64-1.13 (m, 104H, CH_2), 0.92-0.82 (m, 18H, ω^{Myr}-CH_3, ω^{Lau}-CH_3, ω^{Cap}-CH_3);

^{13}C NMR (151 MHz, $CDCl_3$): δ 173.63, 173.41 (C=O, Myr, Lau), 171.48 (C=O, Cap), 171.18 (C=O, NHAc), 170.44 (C=O, br, Myr, Cap), 170.04 (C=O, Myr), 138.91, 138.74, 138.14 (C_q, Ph), 135.77-135.69 (C_q, m, Ph), 128.70, 128.56, 128.48, 128.36, 128.21, 128.15, 128.11, 127.92, 127.83, 127.76, 127.64, 127.53 (CH, Ph), 97.70 (CH, C-1), 97.46 (CH, C-1′), 77.04 (CH, m, C-3), 76.21 (β^{Cap}-*C*H), 75.49 (β^{Cap}-*C*H), 74.11 (CH, m, C-4′, C-5′), 73.58 (CH_2, O*C*H_2Ph), 72.48 (CH, C-3′), 72.18 (CH, C-5), 71.70, 71.15 (CH_2, O*C*H_2Ph), 70.67, 70.18 (β^{Myr}-*C*H), 69.81-69.65 (CH_2, m, OP(O)(O*C*H_2Ph)$_2$), 69.18 (CH, d, $^3J_{C,P}$ = 4.0 Hz, C-4), 68.67 (CH_2, C-6′), 62.19 (CH_2, C-6), 55.88 (CH, C-2), 55.44 (CH, C-2′), 41.46 (α^{Myr}-*C*H_2), 39.74 (α^{Cap}-*C*H_2), 39.54 (α^{Myr}-*C*H_2), 39.09 (α^{Cap}-*C*H_2), 34.62, 34.59, 34.37, 34.26, 32.09, 32.01, 31.98, 29.91, 29.87, 29.83, 29.79, 29.77, 29.74, 29.62, 29.55, 29.53, 29.47, 29.46, 29.43, 29.38, 25.38, 25.36, 25.25, 25.24, 25.19 (CH_2, Myr, Lau), 23.42 (CH_3, NH*Ac*), 22.84, 22.81 (CH_2, Myr, Lau), 14.25 (ω^{Myr}-*C*H_3, ω^{Lau}-*C*H_3, ω^{Cap}-*C*H_3);

^{31}P NMR (243 MHz, $CDCl_3$): δ -1.26, -2.04; HRMS (ESI) *m/z* calcd. for $[M+COOH]^-$ $C_{138}H_{207}N_2O_{28}P_2$ 2402.4316, found 2402.4312.

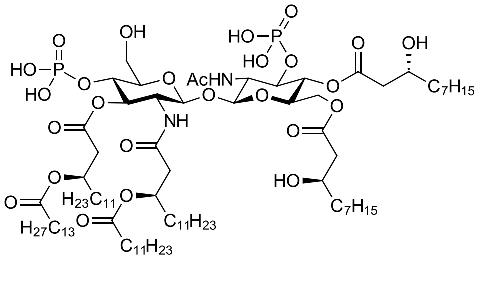

2-Deoxy-2-[(*R*)-3-(dodecanoyloxy)tetradecanoylamino]-4-*O*-phosphoryl-3-*O*-[(*R*)-3-(tetradecanoyloxy)tetradecanoyl]-β-D-glucopyranosyl-(1↔1)-2-acetamido-4,6-di-*O*-[(*R*)-3-(hydroxy)decanoyl]-3-*O*-phosphoryl-2-deoxy-β-D-glucopyranoside (*ββ*-DLAM 919)

A pressure reactor was charged with palladium black (60 mg) and a solution of **23** (4 mg, 1.7 µmol) in a dry toluene - MeOH mixture (2:1, 18 mL) was added. The vessel was closed, purged with argon and then filled with hydrogen (8.5 bar) under stirring. The mixture was stirred for 24 h, the solids were removed by filtration (membrane filter, regenerated cellulose;

0.45 µm pore size) and the solvents were removed under reduced pressure. The residue was subjected by size exclusion chromatography on Bio-Beads S-X1 support (toluene - DCM - MeOH, 5:3:1) which afforded ***ββ*-DLAM 919** (1.5 mg, 51%).

R_f = 0.42 (chloroform - MeOH - water, 100:75:15); ^{1}H NMR (600 MHz, $CDCl_3$ / MeOD 5:3): δ 5.22-5.07 (m, 3H, H-3′, β^{Myr}-C*H*), 4.90 (m, 1H, H-4), 4.72-4.64 (m, 2H, H-1, H-1′), 4.30-4.21 (m, 2H, H-3, H-6b), 4.16 (m, 1H, H-4′), 4.10-3.88 (m, 4H, H-6a, H6b′, β^{Cap}-C*H*), 3.80-3.63 (m, 4H, H-2, H-5, H-2′, H-6a′), 2.65 (dd, 1H, ^2J = 16.1 Hz, ^3J = 7.3 Hz, α^{Myr}-CH_2), 2.60-2.20 (m, 11H, α^{Cap}-CH_2, α^{Myr}-CH_2, α^{Lau}-CH_2), 1.94 (s, 3H, CH_3, NHAc), 1.73-1.04 (m, 104H, CH_2), 0.91-0.77 (m, 18H, ω^{Myr}-CH_3, ω^{Lau}-CH_3, ω^{Cap}-CH_3);

^{13}C NMR (151 MHz, $CDCl_3$ / MeOD 5:3): δ 174.3 (C=O, Myr, Lau), 173.6 (C=O, NHAc), 172.2, 172.0 (C=O, Myr, Cap), 99.4 (CH, m, C-1, C-1′), 76.2 (CH, C-5′), 74.7 (CH, C-3), 73.5 (CH, C-3′), 72.2 (CH, C-5), 70.9 (β^{Myr}-*C*H), 70.6 (CH, C-4′), 70.4 (β^{Myr}-*C*H), 70.0 (CH, C-4), 68.5 (β^{Cap}-*C*H), 62.8 (CH_2, C-6), 60.3 (CH_2, C-6′), 55.0 (CH, C-2), 53.5 (CH, C-2′), 42.3, 41.2 (α^{Cap}-CH_2, α^{Myr}-CH_2), 39.0 (α^{Myr}-CH_2), 37.4, 34.7, 34.3, 32.1-28.0, 25.5 25.2, 22.8 (CH_2, Myr, Lau), 22.8 (CH_3, NH*Ac*), 14.0 (ω^{Myr}-CH_3, ω^{Lau}-CH_3, ω^{Cap}-CH_3);

^{31}P NMR (243 MHz, $CDCl_3$ / MeOD 5:3): δ 1.42, 0.57; MALDI-TOF MS: *m/z* calcd. for $[M-H]^-$ $C_{88}H_{163}N_2O_{26}P_2$ 1726.10, found 1725.86.

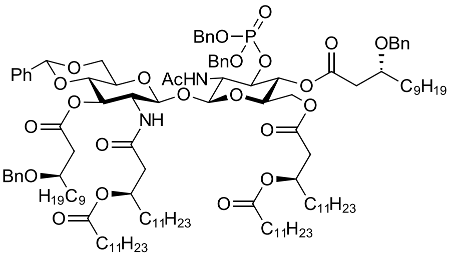

4,6-*O*-Benzylidene-3-*O*-[(*R*)-3-(benzyloxy)dodecanoyl]-2-deoxy-2-[(*R*)-3-(dodecanoyloxy)tetradecanoylamino]-β-D-glucopyranosyl-(1↔1)-2-acetamido-4-*O*-[(*R*)-3-(benzyloxy)dodecanoyl]-3-*O*-[bis(benzyloxy)phosphoryl]-2-deoxy-6-*O*-[(*R*)-3-(dodecanoyloxy)tetradecanoyl]-β-D-glucopyranoside (25)

To a stirred solution of **24** (29 mg, 0.018 mmol) in dry THF (1 mL) a solution of TREAT-HF (0.80 mL) was added over a period of 30min at 0°C and the reaction mixture was stirred for 20 h at r.t. The mixture was diluted with DCM (50 mL) and washed with sat. aq. $NaHCO_3$ (2 x 20 mL) and brine (20 mL). The organic layer was dried over Na_2SO_4, filtered and concentrated. The residue was purified by column chromatography on silica gel (toluene – EtOAc, 1:1 → 0:1, v/v) and by size exclusion chromatography on Sephadex LH-20 (EtOAc) to give the 3′-OH intermediate as white solid (26 mg, 95 %). To a stirred solution of 3′-OH intermediate (24 mg, 0.016 mmol) in dry DCM (2 mL) fatty acid **FA2** (6 eq., 0.94 mmol), DIC (6 eq., 0.94 mmol) and DMAP (0.5 eq., 0.009 mmol) were successively added. The reaction mixture was stirred for 12 h at r.t., diluted with DCM (30 mL) and washed with sat. aq. $NaHCO_3$ (10 mL) and brine (10 mL). The organic layer was dried over Na_2SO_4, filtered and concentrated. The residue was purified by column chromatography on silica gel (toluene – EtOAc, 1:1, v/v) and size exclusion chromatography on Bio-Beads S-X1 support (toluene-DCM, 3:1) to give **25** (28 mg, 85 %; 78% over two steps).

R_f = 0.39 (toluene - EtOAc, 1:1); $[\alpha]_D^{20}$ = -6.0 (c 1.1, $CHCl_3$); ^{1}H NMR (600 MHz, $CDCl_3$): δ 7.37-7.19 (m, 25H, Ph), 6.46 (d, 1H, $^3J_{NH',2'}$ = 8.8 Hz, N*H'*), 5.85 (d, 1H, $^3J_{NH,2}$ = 7.6 Hz, N*H*), 5.40 (s, 1H, OC*H*Ph), 5.38 (dd, 1H, $^3J_{2',3'}$ = $^3J_{3',4'}$ = 9.9 Hz, H-3′), 5.21-5.15 (m, 2H, H-1, β^{Myr}-C*H*), 5.15-5.09 (m, 2H, H-3, β^{Myr}-C*H*), 5.01-4.88 (m, 6H, H-4, H-1′, OP(O)(OCH_2Ph)$_2$), 4.48 (d, 1H, $^2J_{A,B}$ = 11.5 Hz, OCH_2Ph), 4.43 (d, 1H, $^2J_{A,B}$ = 11.5 Hz, OCH_2Ph), 4.37 (d, 1H, $^2J_{A,B}$ = 11.7 Hz, OCH_2Ph), 4.36 (d, 1H, $^2J_{A,B}$ = 11.8 Hz, OCH_2Ph), 4.32 (dd, 1H, $^2J_{6a',6b'}$ = 10.5 Hz, $^3J_{5',6b'}$ = 4.9 Hz, H-6b′), 4.09-4.01 (m, 2H, H-6a, H-6b) 3.97 (ddd, 1H, $^3J_{1',2'}$ = $^3J_{2',3'}$ = $^3J_{2',NH'}$ = 9.5 Hz, H-2′), 3.78 (m, 1H, β^{Lau}-C*H*), 3.75-3.69 (m, 2H, H-6a′, β^{Lau}-C*H*), 3.67-3.62 (m, 2H, H-5, H-4′), 3.47 (ddd, 1H, $^3J_{4',5'}$ = $^3J_{5',6a'}$ = 9.7 Hz, $^3J_{5',6b'}$ = 5.0 Hz, H-5′), 3.35 (m, 1H, H-2), 2.67 (dd, 1H, ^2J = 15.0 Hz, ^3J = 6.5 Hz, α^{Lau}-CH_2), 2.54 (dd, 1H, ^2J = 15.4 Hz, ^3J = 7.9 Hz, α^{Myr}-CH_2), 2.48-2.36 (m, 5H, α^{Myr}-CH_2, α^{Lau}-CH_2), 2.33-2.26 (m, 5H, α^{Lau}-CH_2, α^{Myr}-CH_2), 1.82 (s, 3H, CH_3, NHAc), 1.64-1.40 (m, 12H, β^{Lau}-CH_2, γ^{Myr}-CH_2, γ^{Lau}-CH_2), 1.38-1.07 (m, 96H, CH_2), 0.91-0.84 (m, 18H, ω^{Myr}-CH_3, ω^{Lau}-CH_3);

^{13}C NMR (151 MHz, $CDCl_3$): δ 173.97, 173.53 (C=O, Lau), 171.29, 171.15 (C=O, NHAc, Lau), 170.52, 170.25, 169.86 (C=O, Myr, Lau), 138.73, 137.00 (C_q, Ph), 135.75, 135.70 (C_q, Ph), 129.09, 128.79, 128.74, 128.71, 128.36, 128.24, 128.16, 127.83, 127.62, 127.52, 127.48, 126.21 (CH, Ph), 101.55 (CH, benzylidene acetal), 98.43 (CH, C-1′), 96.29 (CH, C-1), 79.06 (CH, C-4′), 76.22 (CH, d, $^2J_{C,P}$ = 5.1 Hz, C-3), 75.74, 75.56 (β^{Lau}-*C*H), 71.85 (CH, C-5), 71.36 (CH_2, O*C*H_2Ph), 71.31 (CH, C-3′), 71.03 (CH_2, O*C*H_2Ph), 70.98, 70.69 (β^{Myr}-*C*H), 69.83 (CH_2, d, $^2J_{C,P}$ = 5.8 Hz, OP(O)(O*C*H_2Ph)$_2$), 69.73 (CH_2, d, $^2J_{C,P}$ = 5.4 Hz, OP(O)(O*C*H_2Ph)$_2$), 69.62 (CH, C-4), 68.71 (CH_2, C-6′), 66.71 (CH, C-5′), 63.06 (CH_2, C-6), 54.42 (CH, C-2′), 51.76 (CH, C-2), 41.67 (α^{Myr}-*C*H_2), 39.88, 39.79, 39.11 (α^{Myr}-*C*H_2, α^{Lau}-*C*H_2), 34.87, 34.76, 34.69, 34.22, 34.10, 32.08, 29.90, 29.85, 29.81, 29.77, 29.74, 29.63, 29.58, 29.53, 29.49, 29.43, 25.45, 25.42, 25.38, 25.32, 25.21 (CH_2, Myr, Lau), 23.58 (CH_3, NH*Ac*), 22.83 (CH_2, Myr, Lau), 14.25 (ω^{Myr}-*C*H_3, ω^{Lau}-*C*H_3);

^{31}P NMR (243 MHz, $CDCl_3$): δ -1.34; HRMS (ESI) *m/z* calcd. for $[M+COOH]^-$ $C_{126}H_{196}N_2O_{25}P$ 2168.3870, found 2168.3900.

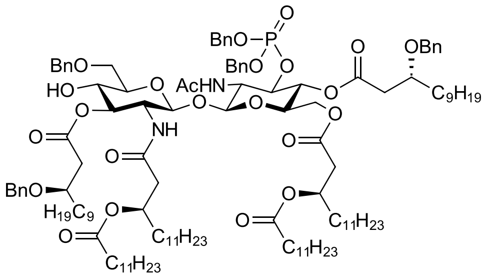

6-*O*-Benzyl-3-*O*-[(*R*)-3-(benzyloxy)dodecanoyl]-2-deoxy-2-[(*R*)-3-(dodecanoyloxy)tetradecanoylamino]-β-D-glucopyranosyl-(1↔1)-2-acetamido-4-*O*-[(*R*)-3-(benzyloxy)dodecanoyl]-3-*O*-[bis(benzyloxy)phosphoryl]-2-deoxy-6-*O*-[(*R*)-3-(dodecanoyloxy)tetradecanoyl]-β-D-glucopyranoside (26)

A solution of **25** (8 mg, 0.004 mmol) in dry DCM (3 mL) was stirred with powdered activated molecular sieves (4Å, 0.1 g) under atmosphere of Ar for 1 h at r.t. The mixture was cooled to -78 °C and Et_3SiH (6 eq, 0.022 mmol, 4 µl) followed by TfOH (10 eq, 0,036 mmol, 4 µl) were added under atmosphere of Ar. The reaction was stirred at -78 °C for 1 h, then Et_3N (5 µl) and MeOH (50 µl) were successively added, and the mixture was stirred for 10 min. The

reaction mixture was brought to r.t., diluted with DCM (20 mL), filtered over a pad of Celite and washed successively with sat. aq. $NaHCO_3$ (10 mL), water (10 mL) and brine (10 mL). The organic layer was dried over Na_2SO_4, filtered and concentrated. The residue was purified by HPLC (toluene – EtOAc, 1:1 → 2:1) to give **26 a**s white solid (7 mg, 92%).

R_f = 0.18 (toluene - EtOAc, 1:1); ^{1}H NMR (600 MHz, $CDCl_3$): δ 7.39-7.17 (m, 25H, Ph), 6.27 (d, 1H, $^3J_{NH',2'}$ = 8.9 Hz, N*H′*), 5.80 (d, 1H, $^3J_{NH,2}$ = 7.9 Hz, N*H*), 5.18 (m, 1H, $β^{Myr}$-C*H*), 5.14-4.86 (m, 9H, H-1, H-3, H-4, H-3′, $β^{Myr}$-C*H,* OP(O)(OCH_2Ph)$_2$), 4.83 (d, 1H, $^3J_{1',2'}$ = 8.3 Hz, H-1′), 4.58 (d, 1H, $^2J_{A,B}$ = 12.2 Hz, OCH_2Ph), 4.54 (d, 1H, $^2J_{A,B}$ = 12.9 Hz, OCH_2Ph), 4.52 (d, 1H, $^2J_{A,B}$ = 11.5 Hz, OCH_2Ph), 4.48 (d, 1H, $^2J_{A,B}$ = 11.3 Hz, OCH_2Ph), 4.43 (d, 1H, $^2J_{A,B}$ = 11.6 Hz, OCH_2Ph), 4.36 (d, 1H, $^2J_{A,B}$ = 11.6 Hz, OCH_2Ph), 4.06 (dd, 1H, $^2J_{6a,6b}$ = 12.1 Hz, $^3J_{5,6b}$ = 6.7 Hz, H-6b), 4.02 (dd, 1H, $^2J_{6a,6b}$ = 12.2 Hz, $^3J_{5,6a}$ = 2.3 Hz, H-6a), 3.89-3.82 (m, 2H, H-2′, $β^{Lau}$-C*H*), 3.76-3.67 (m, 3H, H-6a′, H-6b′, $β^{Lau}$-C*H*), 3.65-3.58 (m, 2H, H-5, H-4′), 3.51 (ddd, 1H, $^3J_{4',5'}$ = $^3J_{5',6a'}$ = 9.1 Hz, $^3J_{5',6b'}$ = 4.5 Hz, H-5′), 3.43 (m, 1H, H-2), 2.92 (d, 1H, $^3J_{OH',4'}$ = 3.4 Hz, O*H′*), 2.64 (dd, 1H, ^2J = 15.0 Hz, ^3J = 7.5 Hz, $α^{Lau}$-CH_2), 2.54 (dd, 1H, ^2J = 15.5 Hz, ^3J = 7.7 Hz, $α^{Myr}$-CH_2), 2.52-2.36 (m, 5H, $α^{Myr}$-CH_2, $α^{Lau}$-CH_2), 2.32-2.23 (m, 5H, $α^{Lau}$-CH_2, $α^{Myr}$-CH_2), 1.76 (s, 3H, CH_3, NHAc), 1.66-1.40 (m, 12H, $β^{Lau}$-CH_2, $γ^{Myr}$-CH_2, $γ^{Lau}$-CH_2), 1.38-1.12 (m, 96H, CH_2), 0.92-0.83 (m, 18H, $ω^{Myr}$-CH_3, $ω^{Lau}$-CH_3);

^{13}C NMR (151 MHz, $CDCl_3$): δ 173.79, 173.47 (C=O, Myr, Lau), 172.37 (C=O, Lau), 171.23 (C=O, NHAc), 170.51, 170.14, 169.91 (C=O, Myr, Lau), 138.75, 138.38, 138.00 (C_q, Ph), 135.79, 135.75 (C_q, Ph), 128.71, 128.69, 28.66, 128.47, 128.36, 128.21, 128.14, 128.00, 127.86, 127.77, 127.66, 127.48 (CH, Ph), 97.96 (CH, C-1′), 96.46 (CH, C-1), 76.58 (CH, d, $^2J_{C,P}$ = 6.8 Hz, C-3), 76.12 ($β^{Lau}$-*C*H), 75.77 (CH, C-3′), 75.56 ($β^{Lau}$-*C*H), 74.97 (CH, C-5′), 73.91 (CH_2, O*C*H$_2$Ph), 71.82 (CH, C-5), 71.35 (CH_2, O*C*H$_2$Ph), 71.06, 70.60 ($β^{Myr}$-*C*H), 71.04 (CH_2, O*C*H$_2$Ph), 70.31 (CH, C-4′), 70.01 (CH_2, C-6′), 69.77, 69.73, 69.67 (CH_2, OP(O)(O*C*H$_2$Ph)$_2$), 69.59 (CH, d, $^3J_{C,P}$ = 3.2 Hz, C-4), 62.94 (CH_2, C-6), 56.74 (CH, C-2), 53.54 (CH, C-2′), 41.72 ($α^{Myr}$-*C*H$_2$), 39.92, 39.58, 39.11 ($α^{Myr}$-*C*H$_2$, $α^{Lau}$-*C*H$_2$), 34.85, 34.68, 34.59, 34.36, 34.20, 34.14, 32.08, 29.90, 29.85, 29.82, 29.78, 29.74, 29.70, 29.68, 29.57, 29.54, 29.49, 29.43, 29.41, 25.44, 25.38, 25.36, 25.32, 25.21 (CH_2, Myr, Lau), 23.48 (CH_3, NH*Ac*), 22.84 (CH_2, Myr, Lau), 14.25 ($ω^{Myr}$-*C*H$_3$, $ω^{Lau}$-*C*H$_3$);

^{31}P NMR (243 MHz, $CDCl_3$): δ -1.42; HRMS (ESI) *m/z* calcd. for $[M+COOH]^-$ $C_{126}H_{198}N_2O_{25}P$ 2170.4027, found 2170.4041.

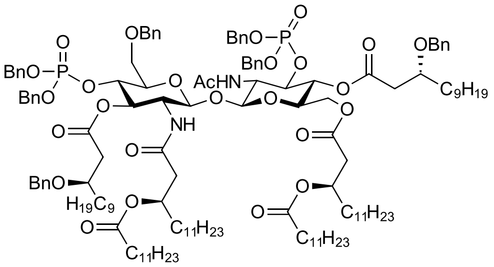

6-*O*-Benzyl-3-*O*-[(*R*)-3-(benzyloxy)dodecanoyl]-4-*O*-[bis(benzyloxy)phosphoryl]-2-deoxy-2-[(*R*)-3-(dodecanoyloxy)tetradecanoylamino]-β-D-glucopyranosyl-(1↔1)-2-acetamido-4-*O*-[(*R*)-3-(benzyloxy)dodecanoyl]-3-*O*-[bis(benzyloxy)phosphoryl]-2-deoxy-6-*O*-[(*R*)-3-(dodecanoyloxy)tetradecanoyl]-β-D-glucopyranoside (27)

To a stirred solution of **26** (8 mg, 0.004 mmol) in dry DCM (2 mL) bis(benzyloxy)-(diisopropylamino)phosphine (0.02 mmol, 8 µL) and a solution of 1*H*-tetrazol in acetonitrile (0.45 M; 6 eq., 50 µl) were successively added under atmosphere of Ar. The reaction mixture was stirred for 1 h, then cooled to -78 °C and a solution of mCPBA (0.03 mmol, 5,0 mg) was added. The reaction mixture was stirred for 1 h at -78 °C, then quenched by addition of Et_3N (10 µL) in MeOH (0.2 mL) and warmed up to r.t. The mixture was diluted with DCM (50 mL) and washed with sat. aq. $NaHCO_3$ (20 mL), water (20 mL) and brine (20 mL). The organic layer was dried over Na_2SO_4, filtered and concentrated. The residue was purified by column chromatography on silica gel (hexane – EtOAc, 1:1, v/v) and by size-exclusion chromatography on Bio Beads S-X1 support (toluene – DCM, 3:1) to give **27** (8 mg, 90%).

R_f = 0.35 (toluene - EtOAc, 1:1); ^{1}H NMR (600 MHz, $CDCl_3$): δ 7.34-7.17 (m, 35H, Ph), 6.12 (d, 1H, $^3J_{NH',2'}$ = 8.5 Hz, N*H*′), 5.84 (d, 1H, $^3J_{NH,2}$ = 8.1 Hz, N*H*), 5.50 (dd, 1H, $^3J_{2',3'}$ = $^3J_{3',4'}$ = 9.7 Hz, H-3′), 5.18 (m, 1H, β^{Myr}-C*H*), 5.08 (m, 1H, β^{Myr}-C*H*), 5.04-4.81 (m, 12H, H-1, H-3, H-4, H-1′, OP(O)(OCH_2Ph)$_2$), 4.52-4.40 (m, 5H, H-4′, OCH_2Ph), 4.38-4.32 (m, 2H, OCH_2Ph), 4.07 (dd, 1H, $^2J_{6a,6b}$ = 12.2 Hz, $^3J_{5,6b}$ = 6.1 Hz, H-6b), 4.02 (dd, 1H, $^2J_{6a,6b}$ = 12.1 Hz, $^3J_{5,6a}$ = 2.1 Hz, H-6a), 3.78 (m, 1H, H-6b′), 3.76-3.70 (m, 2H, β^{Lau}-C*H*), 3.68-3.51 (m, 5H, H-2, H-5, H-2′, H-5′, H-6a′), 2.56 (dd, 1H, ^2J = 15.9 Hz, ^3J = 7.7 Hz, α^{Myr}-CH_2), 2.53-2.47 (m, 2H, α^{Myr}-CH_2, α^{Lau}-CH_2), 2.45-2.35 (m, 3H, α^{Lau}-CH_2), 2.34-2.23 (m, 5H, α^{Myr}-CH_2, α^{Lau}-CH_2), 2.20 (dd, 1H, ^2J = 14.8 Hz, ^3J = 6.1 Hz, α^{Myr}-CH_2), 1.74 (s, 3H, CH_3, NHAc), 1.66-1.39 (m, 12H, β^{Lau}-CH_2, γ^{Myr}-CH_2, γ^{Lau}-CH_2), 1.36-1.12 (m, 96H, CH_2), 0.91-0.84 (m, 18H, ω^{Myr}-CH_3, ω^{Lau}-CH_3);

^{13}C NMR (151 MHz, $CDCl_3$): δ 173.62, 173.55 (C=O, Myr, Lau), 171.20, 171.17 (C=O, NHAc, Lau), 170.48, 170.24, 169.96 (C=O, Myr, Lau), 138.88, 138.77, 138.20 (C_q, Ph), 135.78, 135.73 (C_q, Ph), 128.71, 128.68, 128.66, 128.57, 128.42, 128.36, 128.21, 128.16, 128.14, 128.08, 127.82, 127.70, 127.60, 127.49 (CH, Ph), 97.59 (CH, C-1′), 97.15 (CH, C-1), 76.80 (CH, br, C-3), 75.60, 75.54 (β^{Lau}-*C*H), 74.34 (CH, d, $^3J_{C,P}$ = 5.3 Hz, C-5′), 74.11 (CH, d, $^2J_{C,P}$ = 6.0 Hz, C-4′), 73.64 (CH_2, O*C*H_2Ph), 72.31 (CH, C-3′), 71.89 (CH, C-5), 71.12 (CH_2, O*C*H_2Ph), 70.81, 70.48 (β^{Myr}-*C*H), 69.76, 69.72, 69.68, 69.64, 69.61 (CH_2, OP(O)(O*C*H_2Ph)$_2$), 69.37 (CH, d, $^3J_{C,P}$ = 2.6 Hz, C-4), 68.74 (CH_2, C-6′), 62.66 (CH_2, C-6), 56.31 (CH, C-2), 54.94 (CH, C-2′), 41.58 (α^{Myr}-*C*H_2), 39.38, 39.18, 39.12 (α^{Myr}-*C*H_2, α^{Lau}-*C*H_2), 34.81, 34.65, 34.51, 34.45, 34.26, 34.19, 32.09, 29.92, 29.89, 29.87, 29.83, 29.81, 29.77, 29.74, 29.69, 29.62, 29.55, 29.54, 29.50, 29.47, 29.42, 25.36, 25.23, 25.20 (CH_2, Myr, Lau), 23.44 (CH_3, NH*Ac*), 22.84 (CH_2, Myr, Lau), 14.26 (ω^{Myr}-*C*H_3, ω^{Lau}-*C*H_3);

^{31}P NMR (243 MHz, $CDCl_3$): δ -1.34, -2.08; HRMS (ESI) *m/z* calcd. for $[M+COOH]^-$ $C_{140}H_{211}N_2O_{28}P_2$ 2430.4629, found 2430.4653.

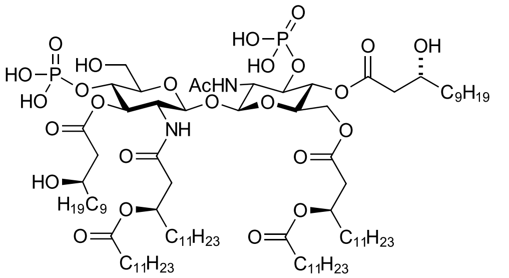

2-Deoxy-2-[(*R*)-3-(dodecanoyloxy)tetradecanoylamino]-3-*O*-[(*R*)-3-(hydroxy)dodecanoyl]-4-*O*-phosphoryl-β-D-glucopyranosyl-(1↔1)-2-acetamido-2-deoxy-6-*O*-[(*R*)-3-(dodecanoyloxy)tetradecanoyl]-4-*O*-[(*R*)-3-(hydroxy)dodecanoyl]-3-*O*-phosphoryl-β-D-glucopyranoside (*ββ*-DLAM 933)

A solution of **27** (7 mg, 0.003 mmol) in toluene−MeOH (3 mL, 1:1) was hydrogenated over Pd-black (5 mg, 47 µmol) for 20 h. The mixture was diluted with toluene – MeOH, 2:1 (10 mL), and the solids were removed by filtration (syringe filter, regenerated cellulose, 45 µm). The filtrate was concentrated, and the residue was purified by size exclusion chromatography on Bio-Beads S-X1 support (200−400 mesh, 100 cm, toluene−DCM - MeOH, 3:1:1) to afford ***ββ*-DLAM 933** (4.0 mg, 85%) as a white solid.

^{1}H NMR (600 MHz, $CDCl_3$ / MeOD 5:3): δ 5.27 (dd, 1H, $^3J_{2',3'}$ = $^3J_{3',4'}$ = 9.8 Hz, H-3′), 5.18 (m, 1H, β^{Myr}-C*H*), 5.08 (m, 1H, β^{Myr}-C*H*), 4.99 (dd, 1H, $^3J_{3,4}$ = $^3J_{4,5}$ = 9.5 Hz, H-4), 4.94 (d, 1H, $^3J_{1,2}$ = 8.4 Hz, H-1), 4.82 (d, 1H, $^3J_{1',2'}$ = 8.1 Hz, H-1′), 4.22 (dd, 1H, $^2J_{6a,6b}$ = 11.9 Hz, $^3J_{5,6b}$ = 3.8 Hz, H-6b), 4.15 (m, 1H, H-4′), 4.07 (m, 1H, H-6a), 4.04-3.97 (m, 2H, β^{Lau}-C*H*), 3.85 (dd, 1H, $^2J_{6a',6b'}$ = 12.9 Hz, $^3J_{5',6b'}$ = 2.8 Hz, H-6b′), 3.77 (d, 1H, $^2J_{6a',6b'}$ = 12.6 Hz, H-6a′), 3.74-3.59 (m, 3H, H-2, H-5, H-2′), 3.40 (m, 1H, H-5′), 2.64-2.36 (m, 7H, α^{Myr}-CH_2, α^{Lau}-CH_2), 2.33-2.21 (m, 5H, α^{Myr}-CH_2, α^{Lau}-CH_2), 1.94 (s, 3H, CH_3, NHAc), 1.63-1.49 (m, 12H, β^{Lau}-CH_2, γ^{Myr}-CH_2, γ^{Lau}-CH_2), 1.35-1.11 (m, 96H, CH_2), 0.89-0.75 (m, 18H, ω^{Myr}-CH_3, ω^{Lau}-CH_3);

^{13}C NMR (151 MHz, $CDCl_3$ / MeOD 5:3): δ 174.0 (C=O, Myr, Lau), 173.4, 171.3, 170.7 (C=O, NHAc, Myr, Lau), 97.9 (CH, C-1′), 97.6 (CH, C-1), 76.2 (CH, C-3), 75.8 (CH, C-5′), 73.3 (CH, C-3′), 71.8 (CH, C-5), 71.6 (CH, C-4′), 71.2, 70.5 (β^{Myr}-*C*H), 69.4 (CH, C-4), 68.6 (β^{Lau}-*C*H), 62.5 (CH_2, C-6), 60.8 (CH_2, C-6′), 55.5 (CH, C-2), 54.1 (CH, C-2′), 41.9, 41.3, 39.2 (α^{Myr}-*C*H_2, α^{Lau}-*C*H_2), 37.4, 37.3, 34.6, 34.3, 34.1, 32.2-28.1, 25.4, 25.2, 22.7 (CH_2, Myr, Lau), 22.7 (CH_3, NH*Ac*), 14.0 (ω^{Myr}-*C*H_3, ω^{Lau}-*C*H_3), All ^{13}C shifts except for those arising from carbonyl carbons were gained from HSQC. Carbonyl ^{13}C shifts were obtained from HMBC;

^{31}P NMR (243 MHz, $CDCl_3$ / MeOD 5:3): δ 0.09, -0.65; MALDI-TOF MS: *m/z* calcd. for $[M-H]^-$ $C_{90}H_{167}N_2O_{26}P_2$ 1754.13, found 1753.99.

References

[1] R. Liu, C. Chanthamontri, H. Han, J. M. Hernandez-Torres, K. V. Wood, S. A. McLuckey, A. Wei, *J. Org. Chem.* **2008**, *73*, 6059-6072.

[2] (a) D. S. Keegan, S. R. Hagen, D. A. Johnson, *Tetrahedron: Asymmetry* **1996**, *7*, 3559-3564; (b) H. G. Bazin, L. S. Bess, M. T. Livesay, K. T. Ryter, C. L. Johnson, J. S. Arnold, D. A. Johnson, *Tetrahedron Lett* **2006**, *47*, 2087-2092; (c) D. Artner, A. Oblak, S. Ittig, J. A. Garate, S. Horvat, C. Arrieumerlou, A. Hofinger, C. Oostenbrink, R. Jerala, P. Kosma, A. Zamyatina, *ACS Chem. Biol.* **2013**, *8*, 2423-2432.

^{1}H NMR (600 MHz, MeOD)

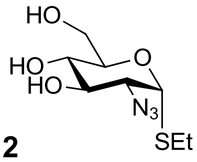

2

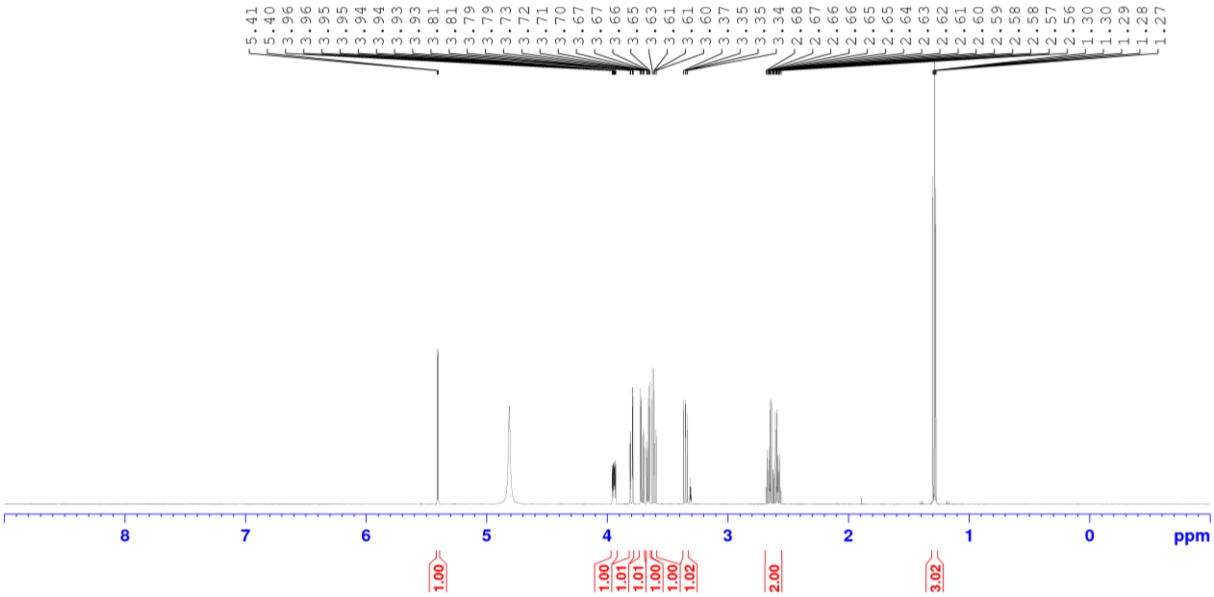

^{13}C NMR (151 MHz, MeOD)

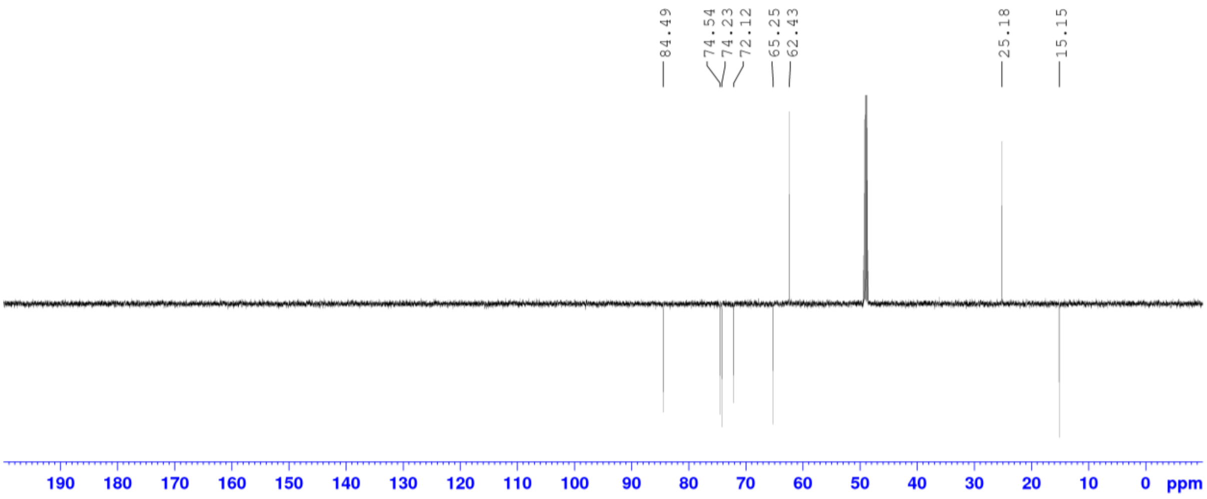

^{1}H NMR (600 MHz, $CDCl_3$)

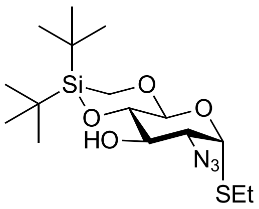

3

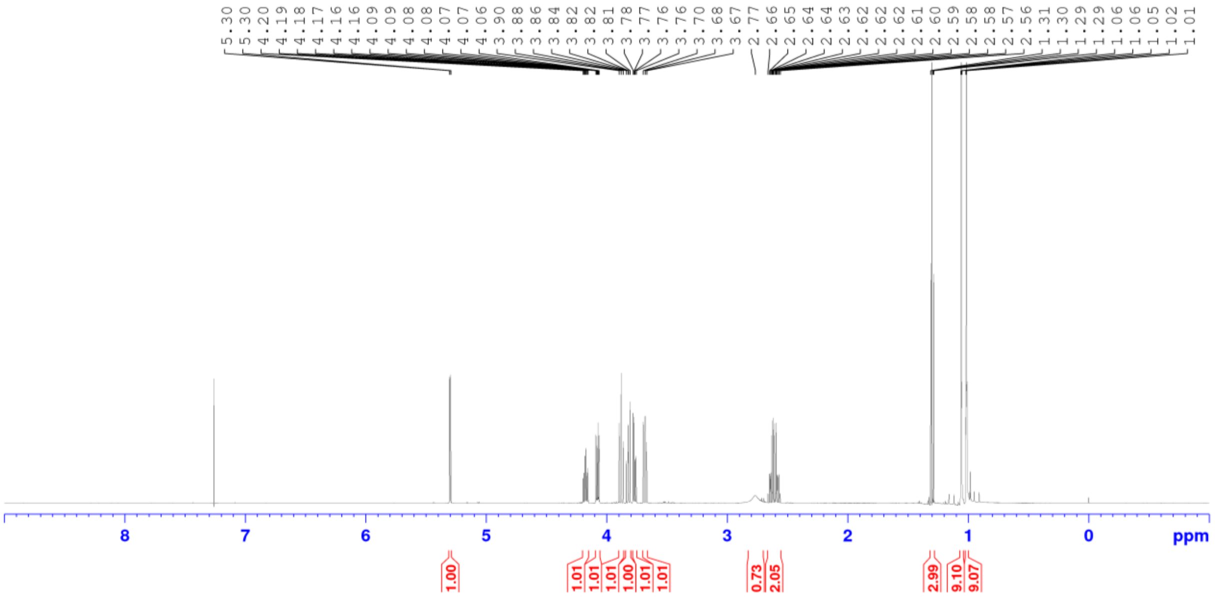

^{13}C NMR (151 MHz, $CDCl_3$)

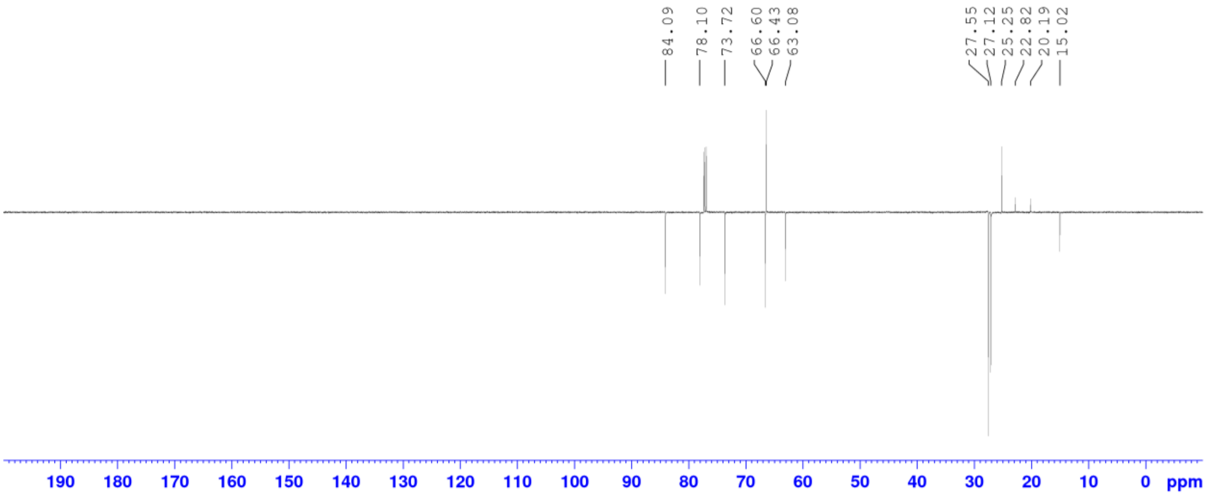

^{1}H NMR (600 MHz, $CDCl_3$)

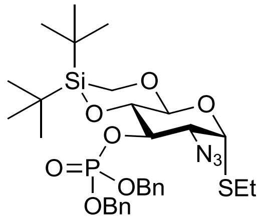

4

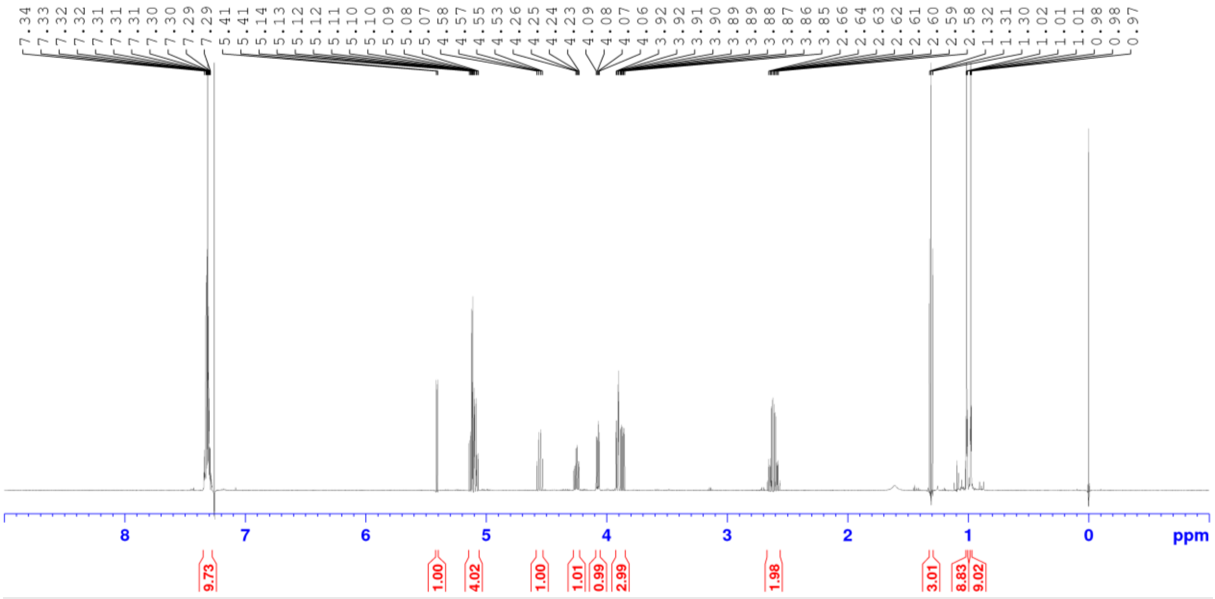

^{13}C NMR (151 MHz, $CDCl_3$)

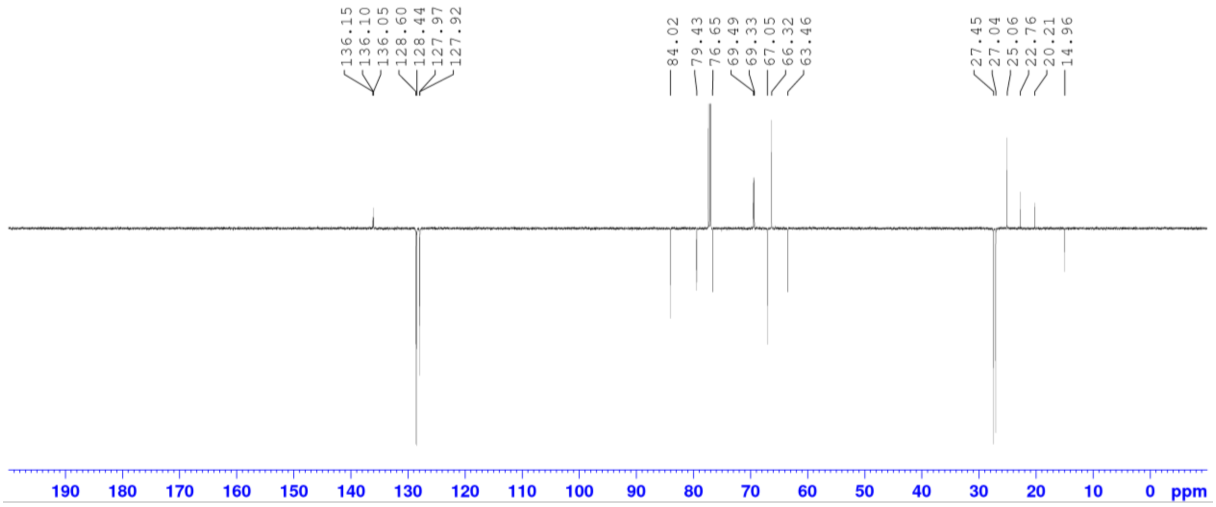

^{1}H NMR (600 MHz, $CDCl_3$)

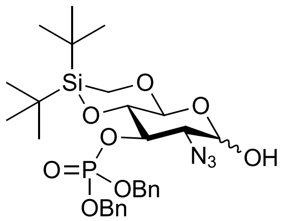

5

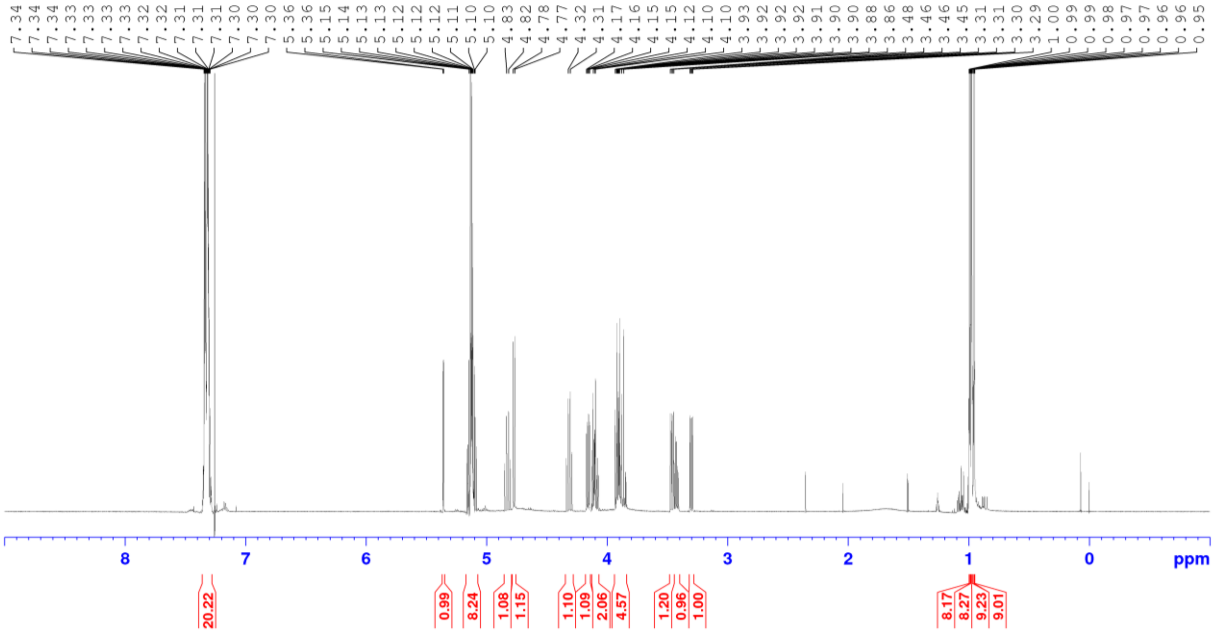

^{13}C NMR (151 MHz, $CDCl_3$)

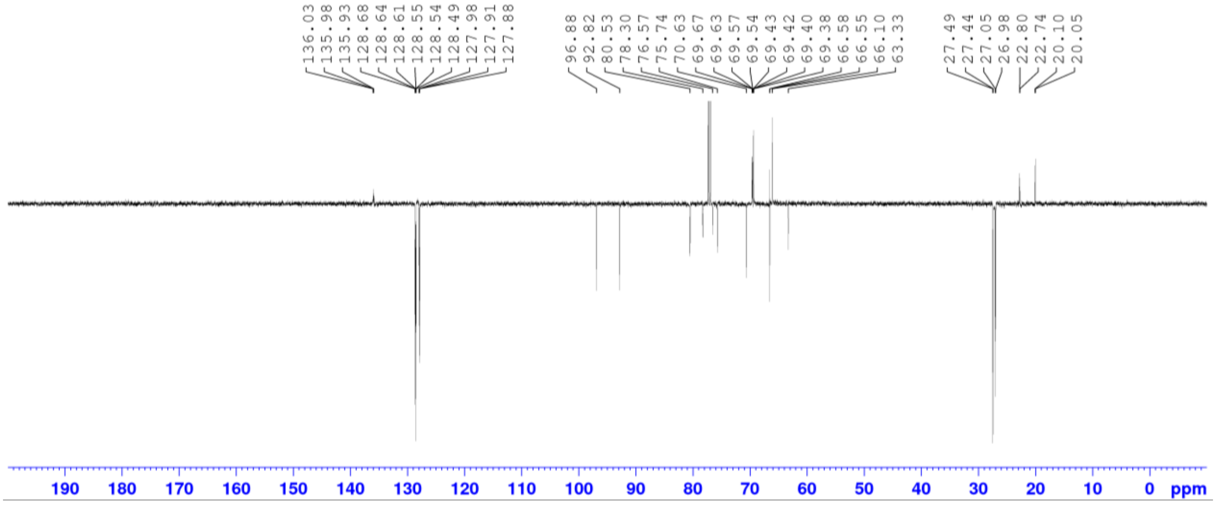

^{1}H NMR (600 MHz, $CDCl_3$)

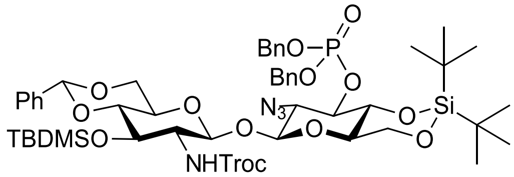

7

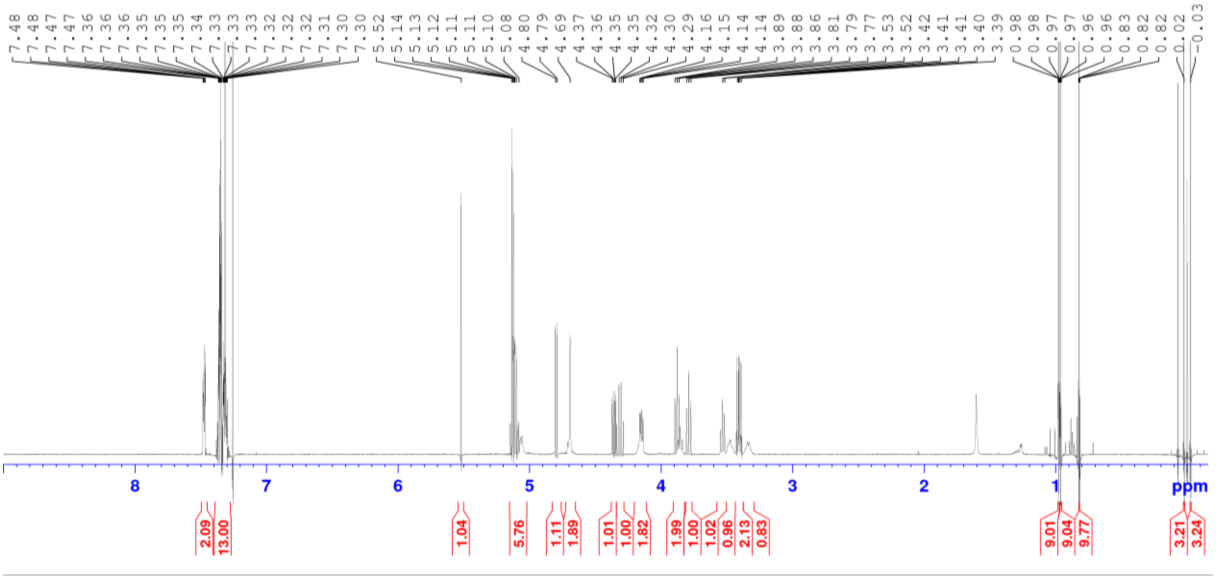

^{13}C NMR (151 MHz, $CDCl_3$)

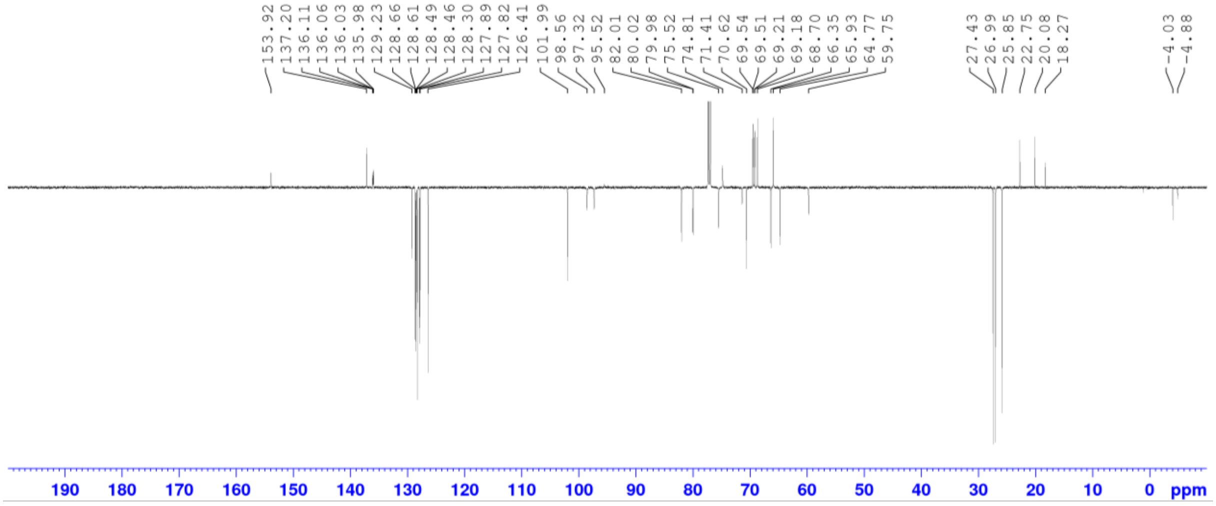

^{1}H NMR (600 MHz, $CDCl_3$)

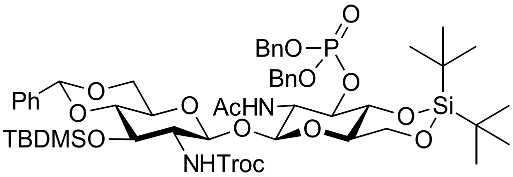

8

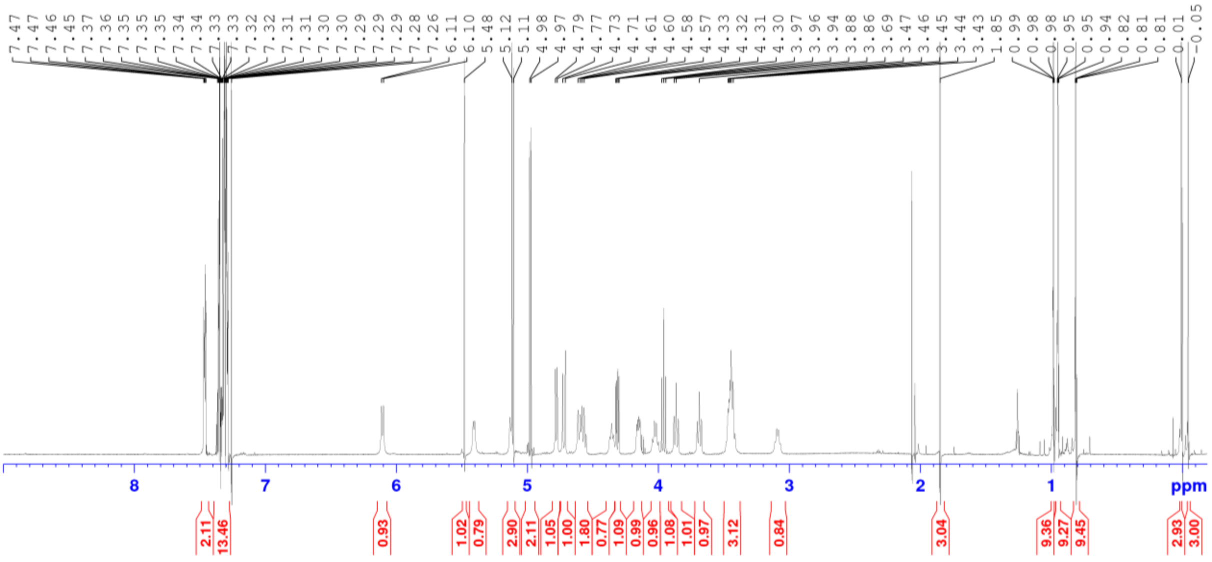

^{13}C NMR (151 MHz, $CDCl_3$)

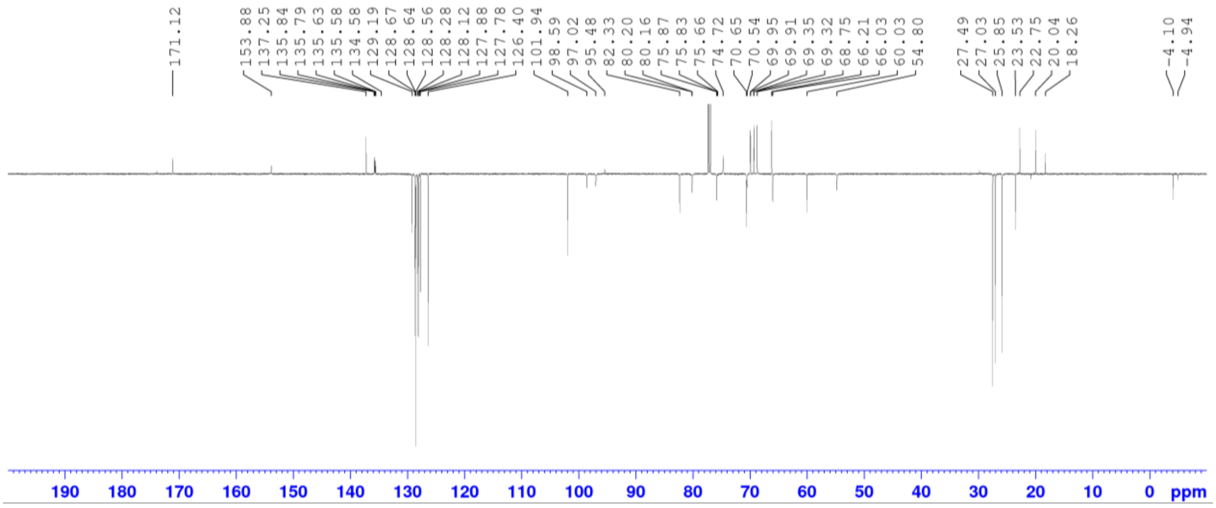

^{1}H NMR (600 MHz, $CDCl_3$)

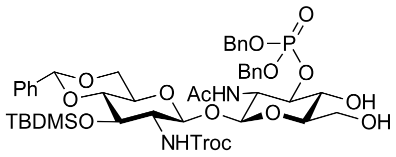

9

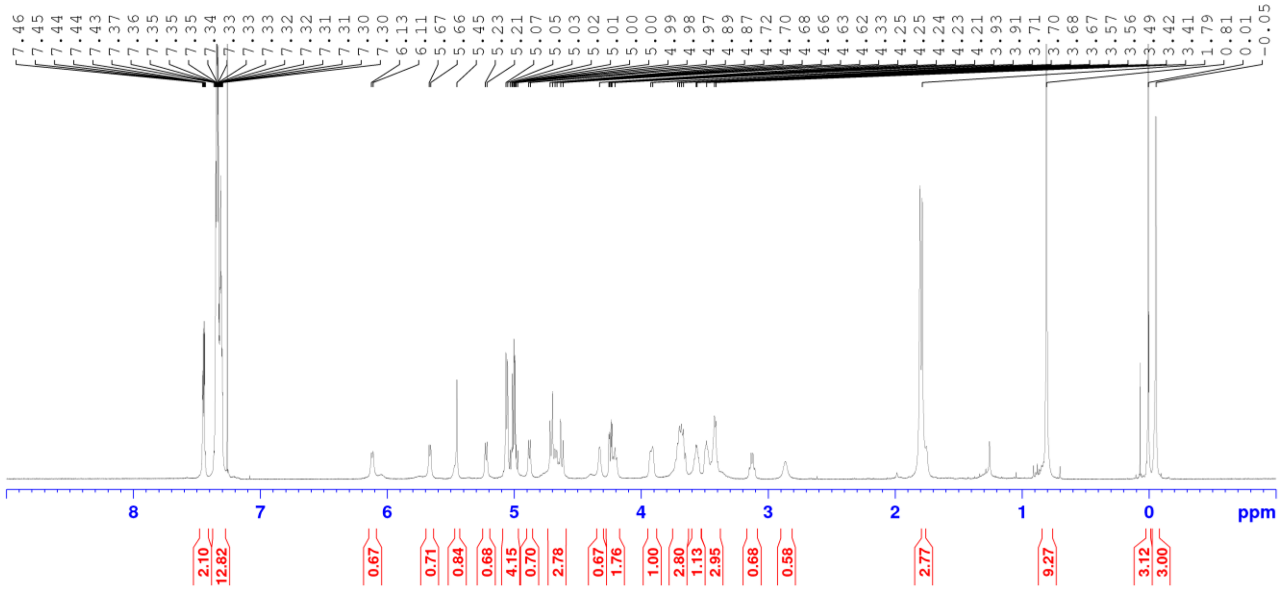

^{13}C NMR (151 MHz, $CDCl_3$)

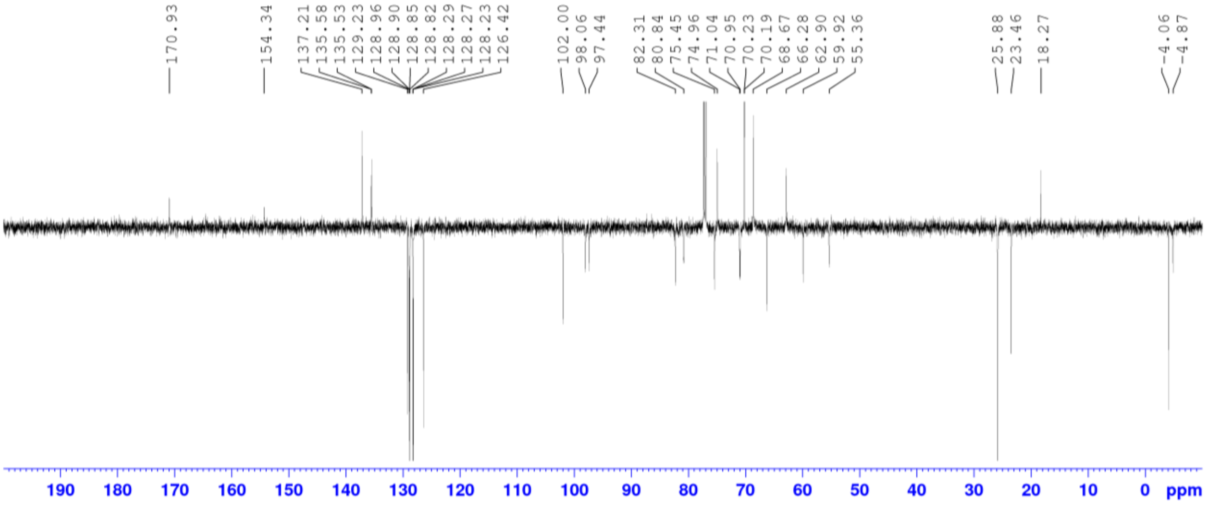

^{1}H NMR (600 MHz, MeOD)

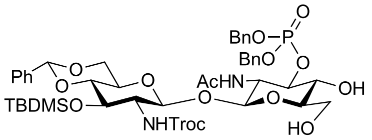

9

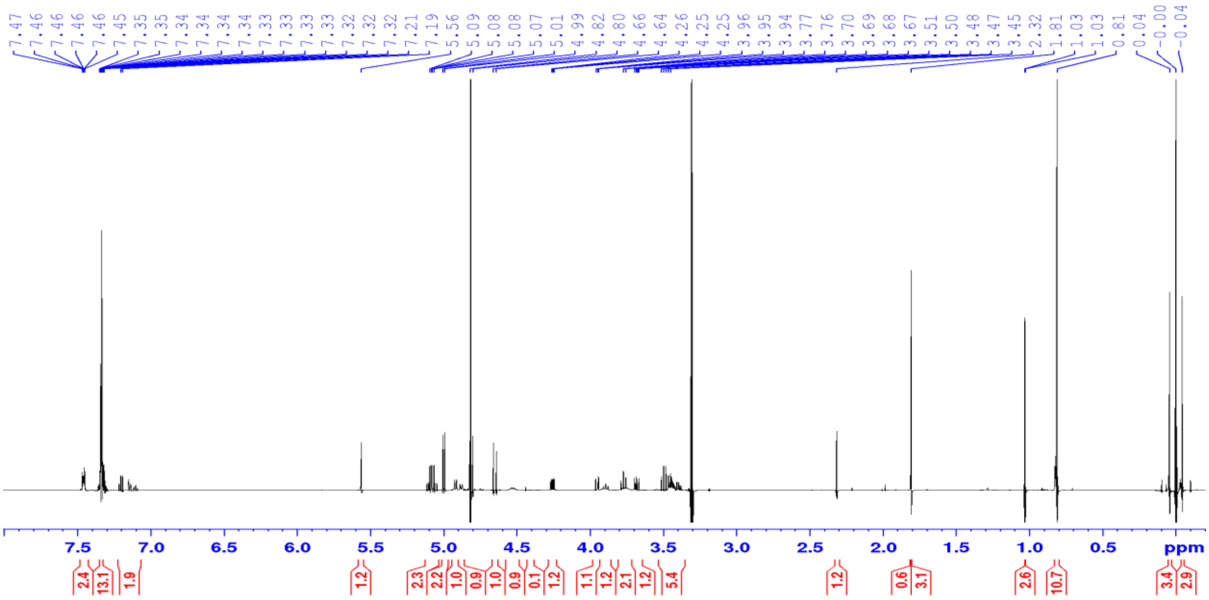

^{13}C NMR (151 MHz, MeOD)

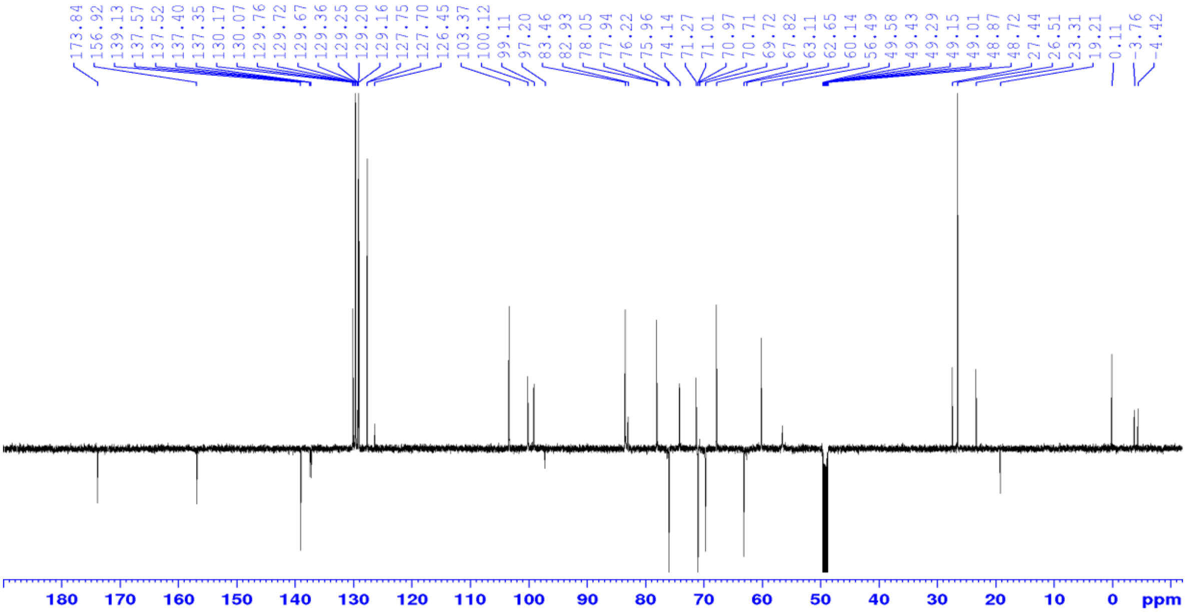

^{1}H NMR (600 MHz, $CDCl_3$)

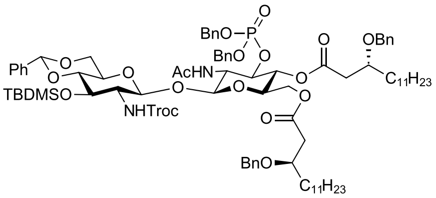

10

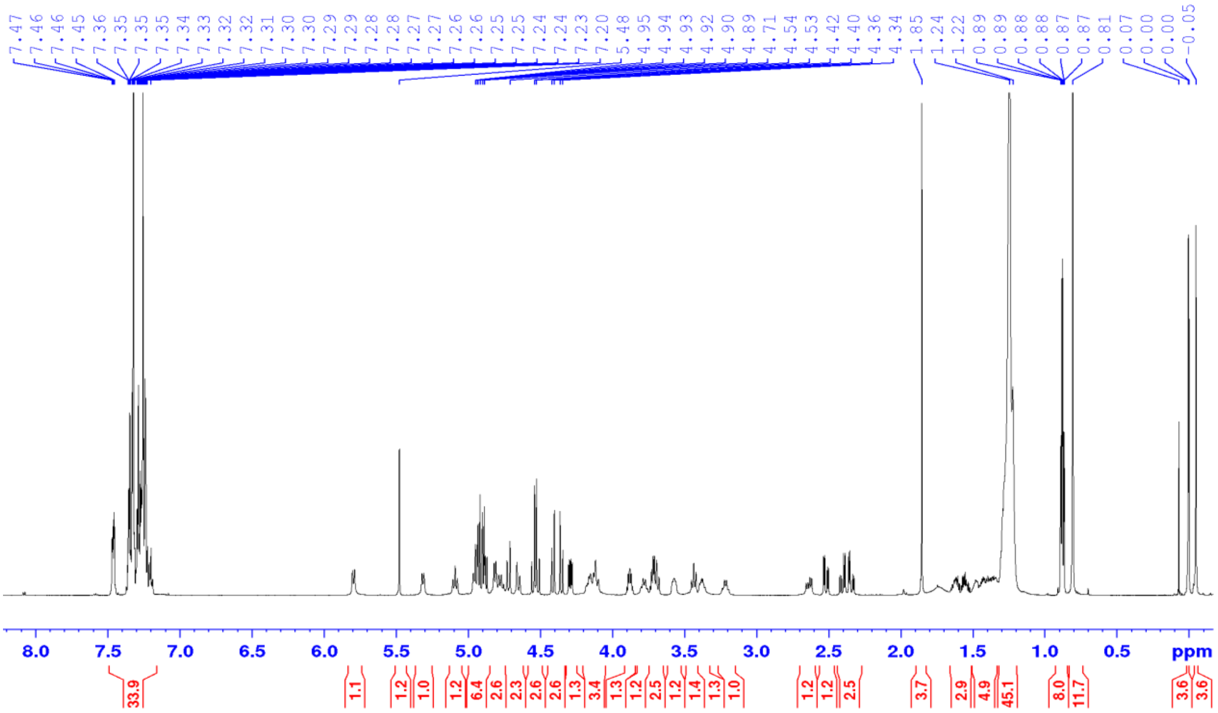

^{13}C NMR (151 MHz, $CDCl_3$)

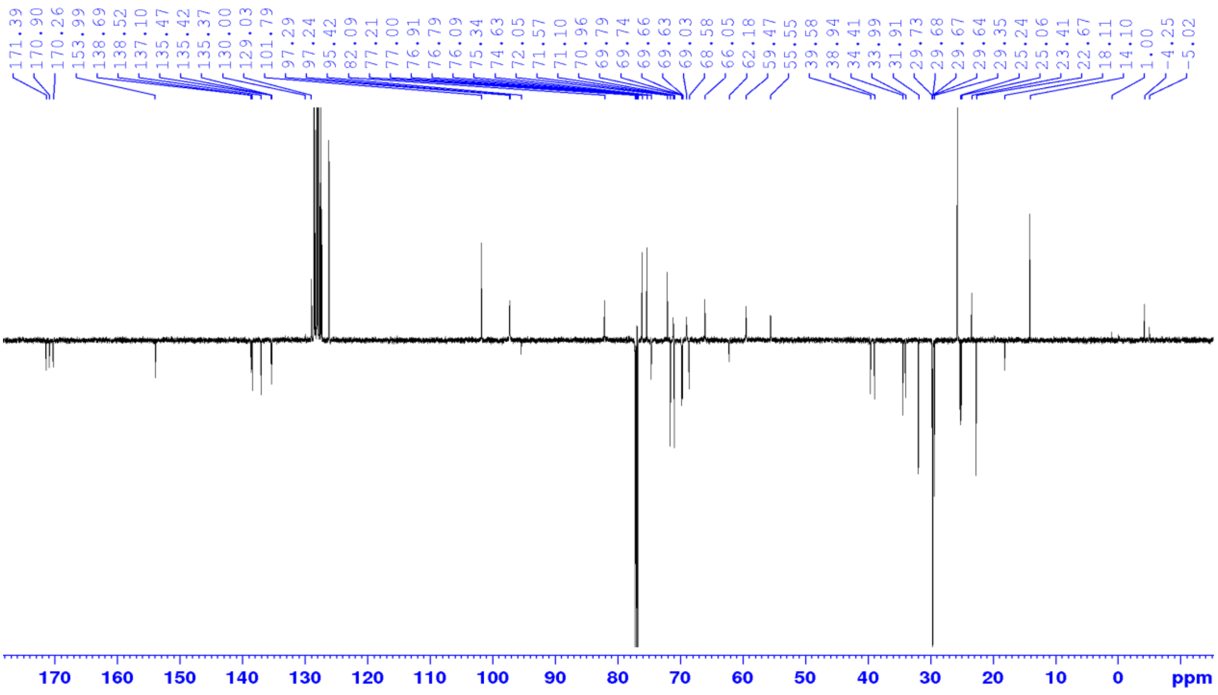

^1H NMR (600 MHz, $CDCl_3$)

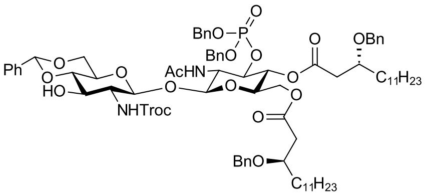

11

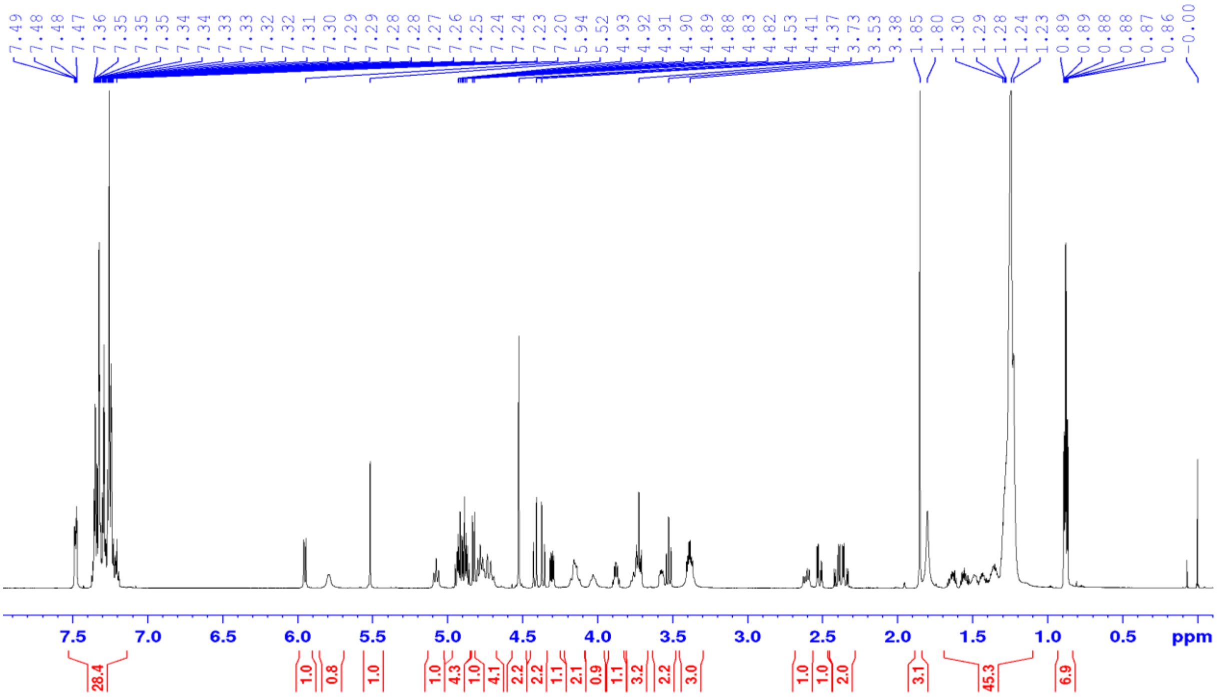

^{13}C NMR (151 MHz, $CDCl_3$)

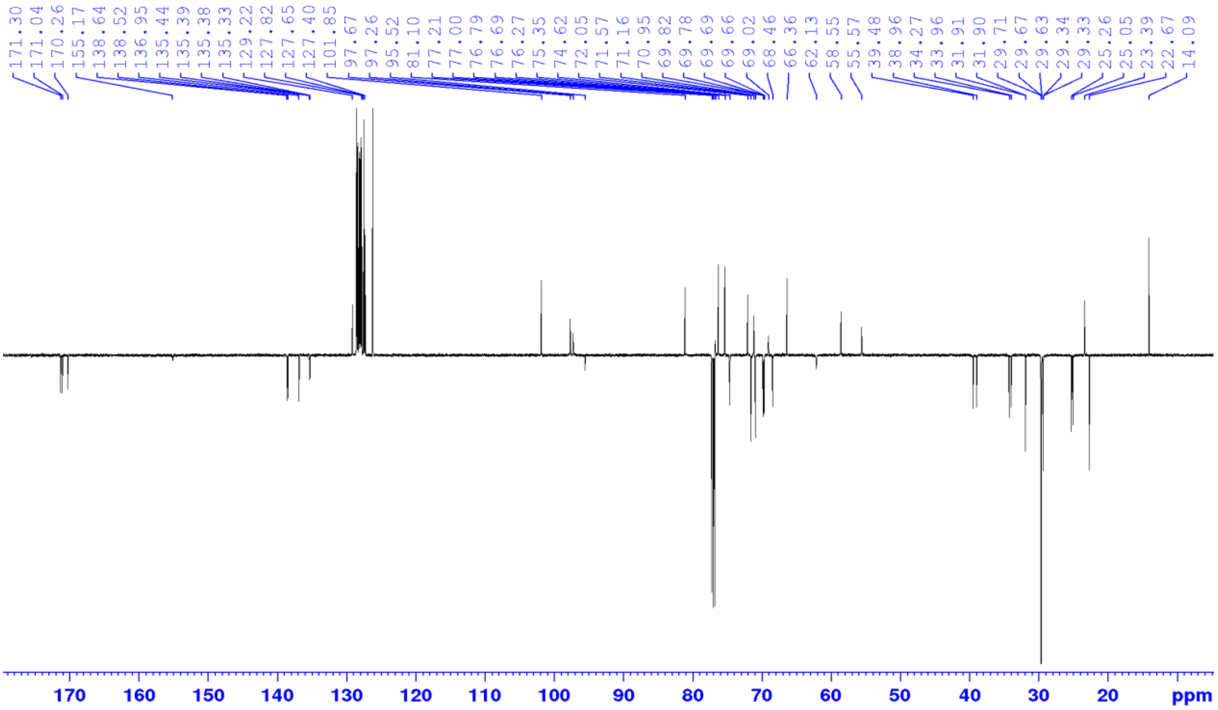

^{1}H NMR (600 MHz, $CDCl_3$)

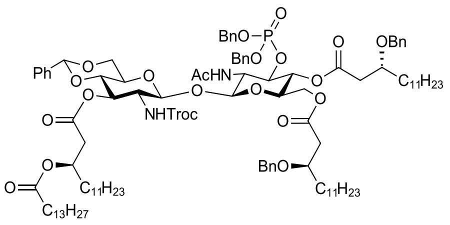

12

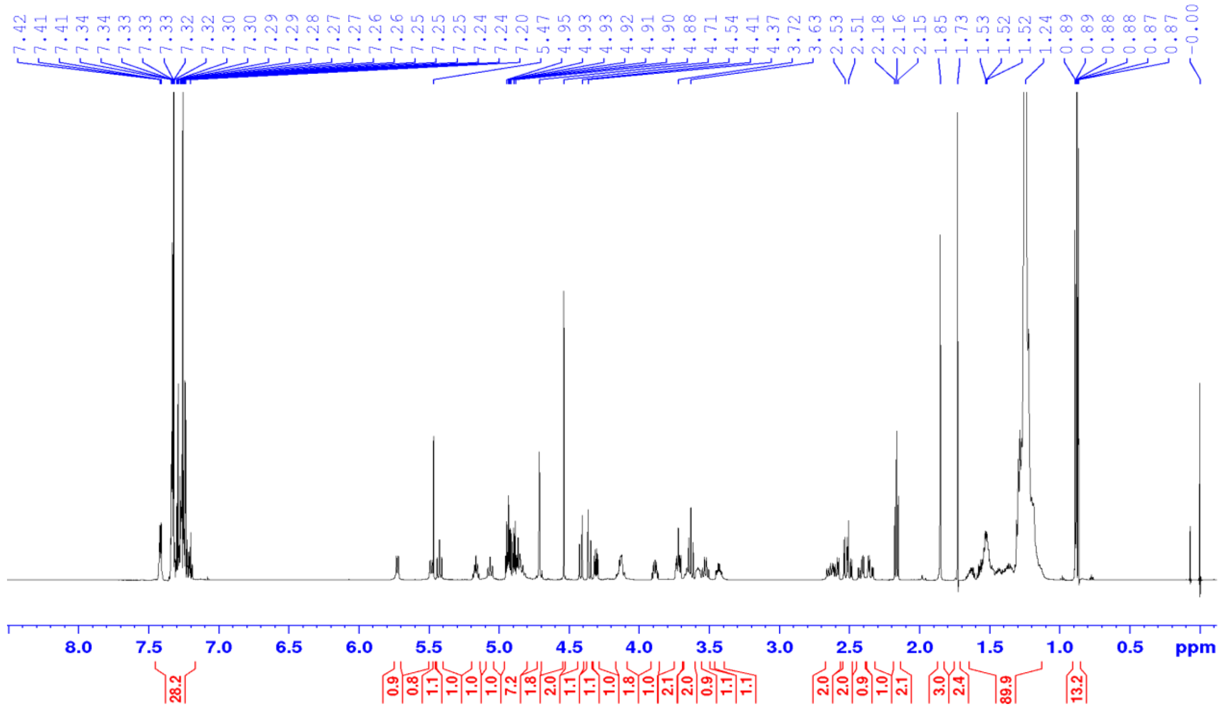

^{13}C NMR (151 MHz, $CDCl_3$)

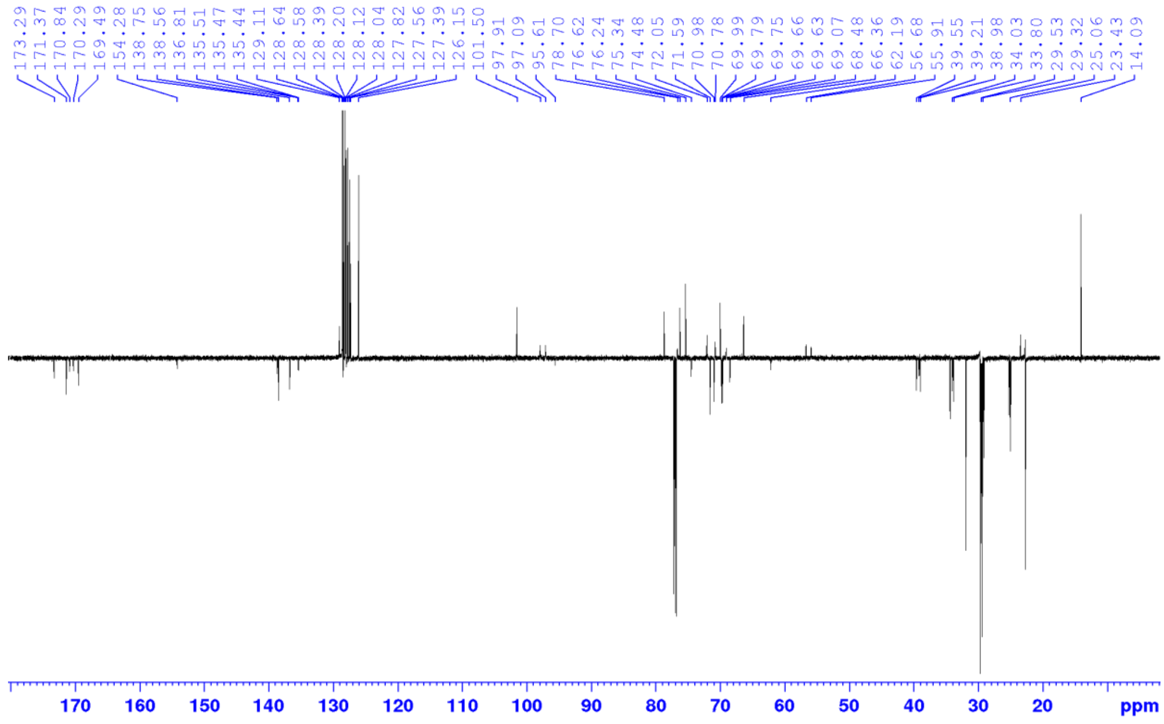

^{1}H NMR (600 MHz, $CDCl_3$)

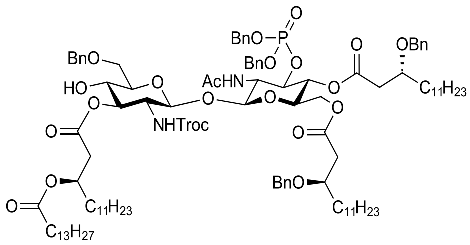

13

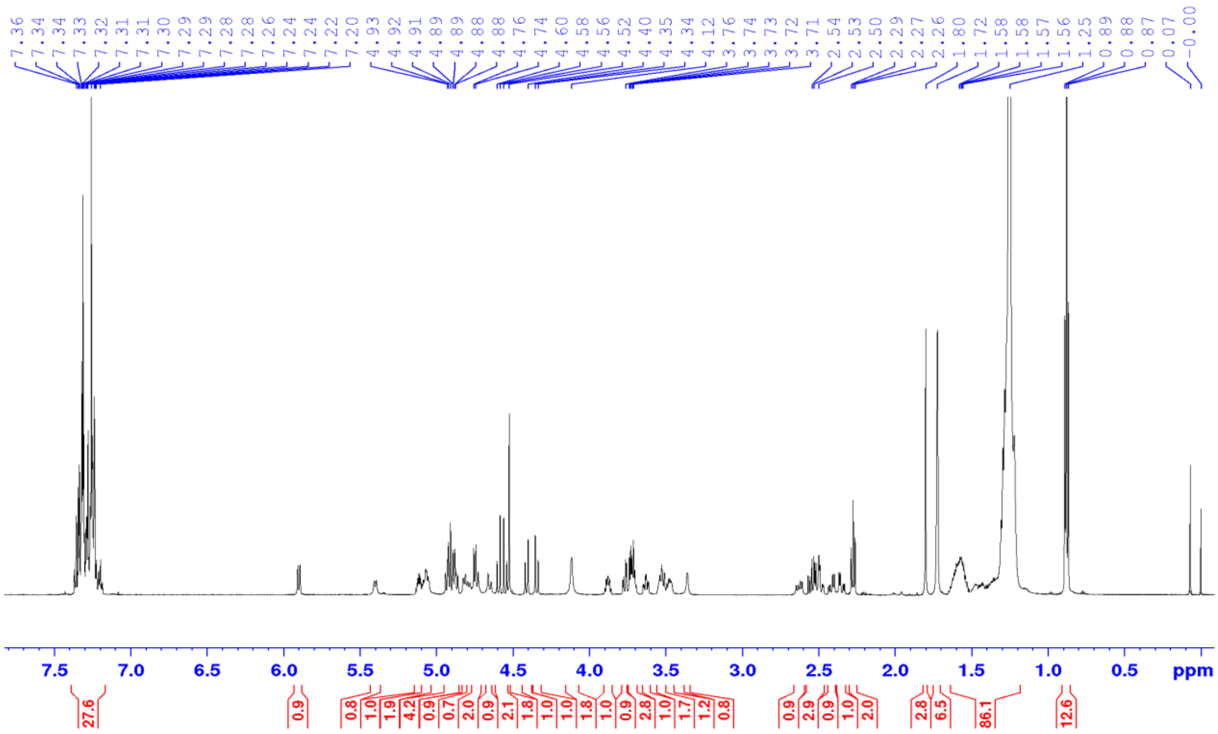

^{13}C NMR (151 MHz, $CDCl_3$)

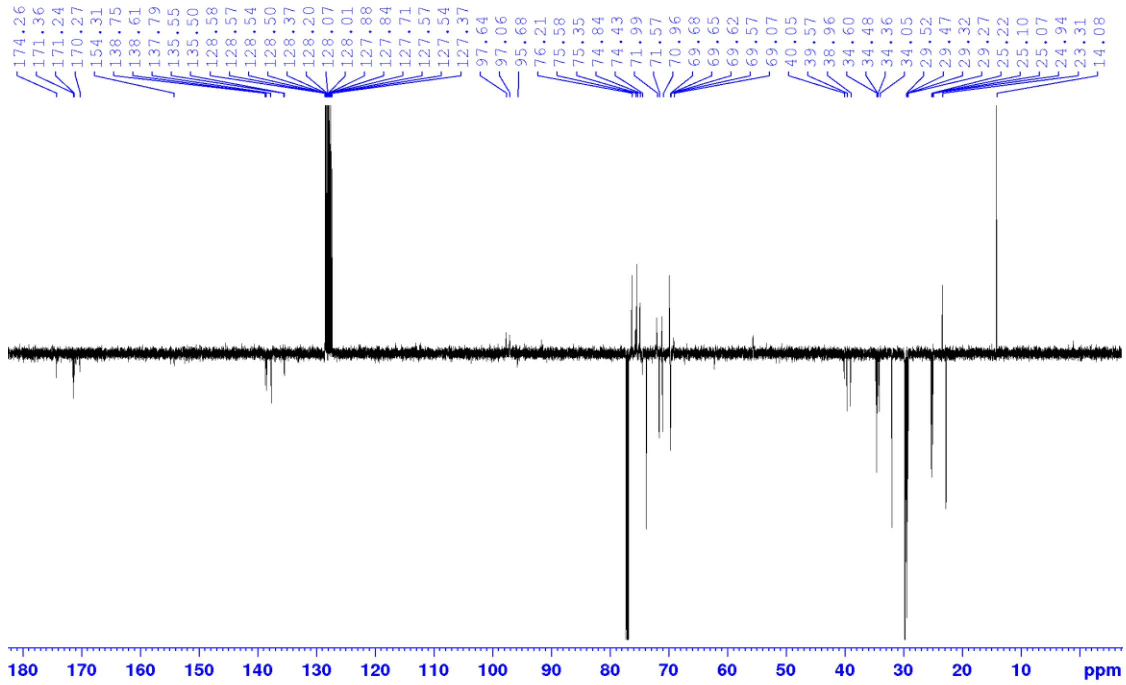

^{1}H NMR (600 MHz, $CDCl_3$)

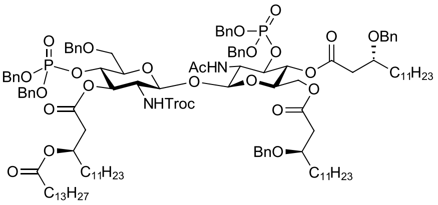

14

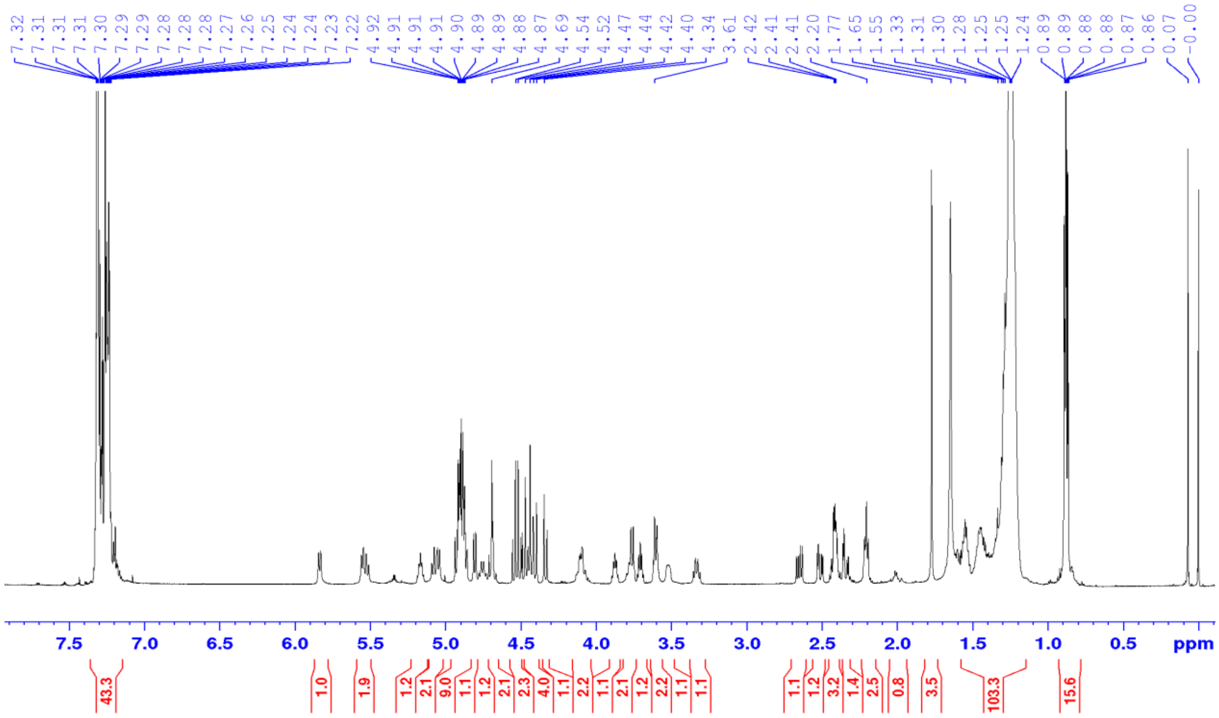

^{13}C NMR (151 MHz, $CDCl_3$)

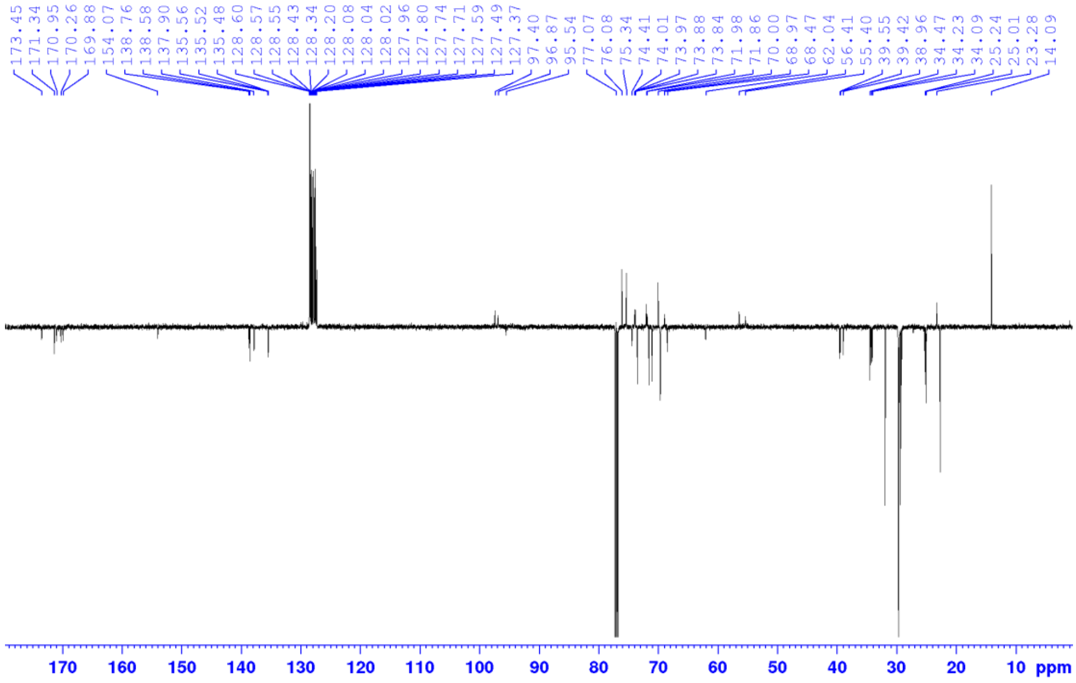

^{1}H NMR (600 MHz, $CDCl_3$)

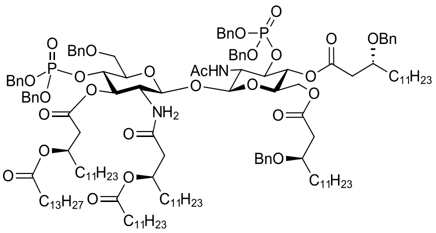

15

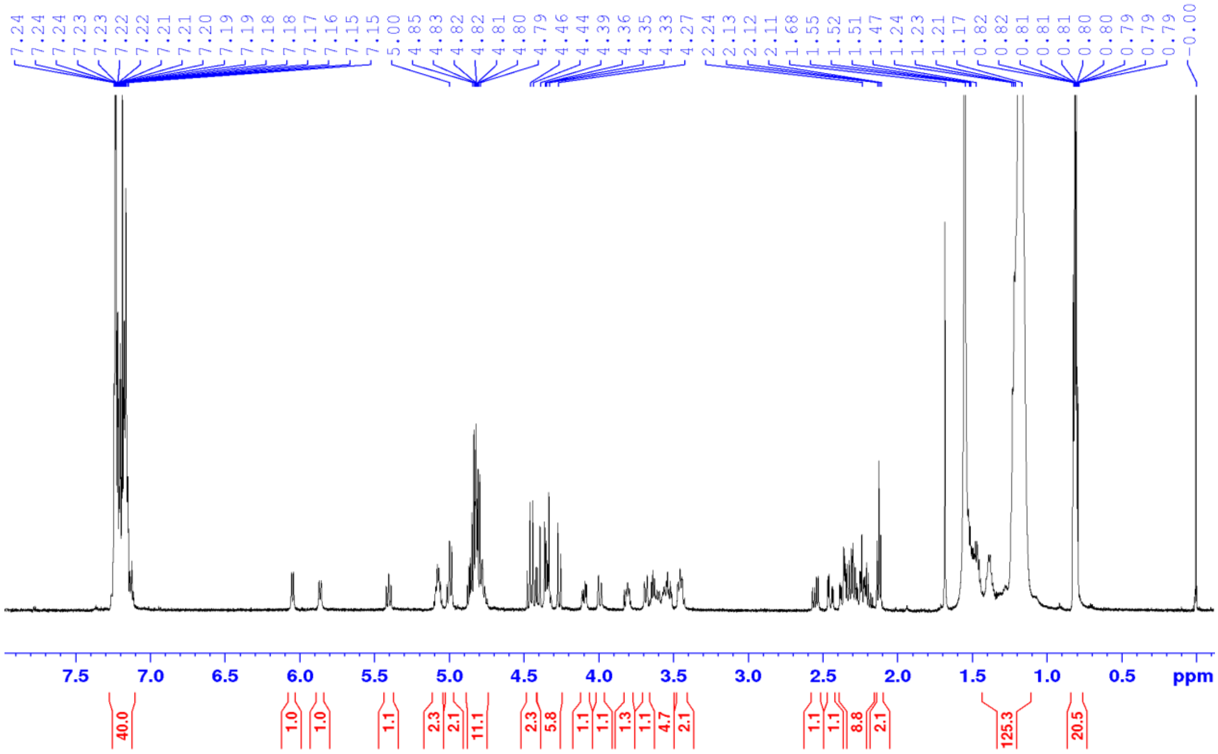

^{13}C NMR (151 MHz, $CDCl_3$)

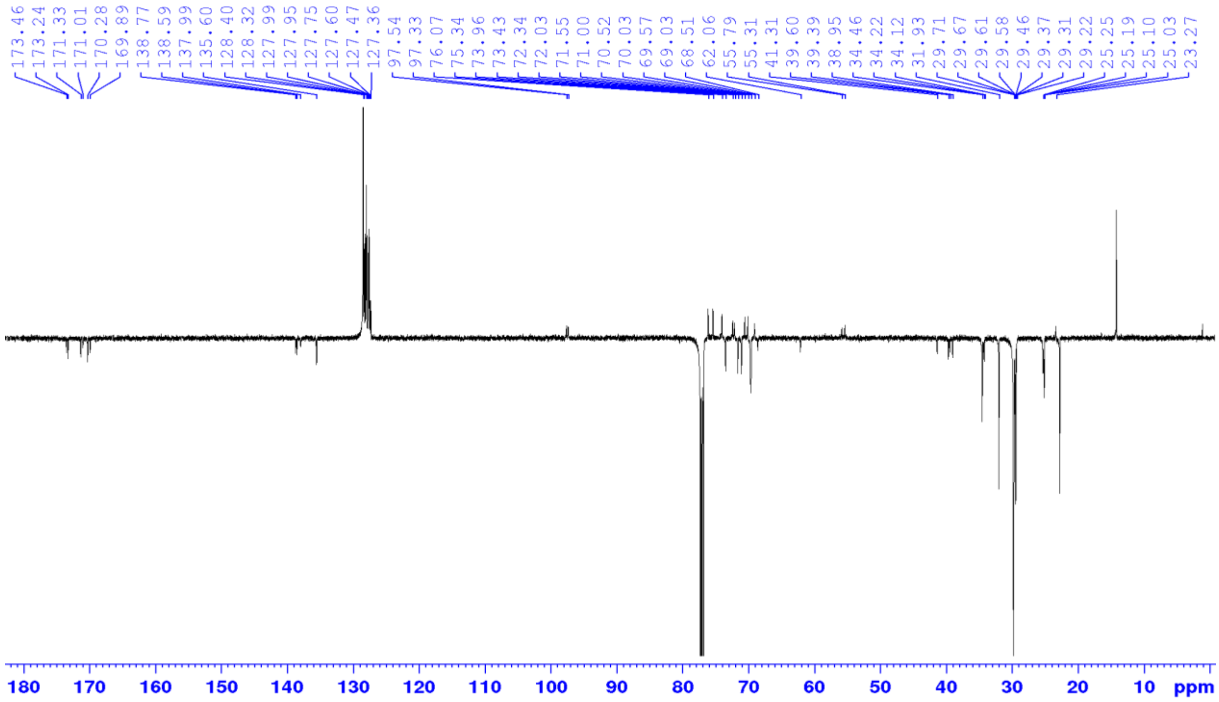

^{1}H NMR (600 MHz, $CDCl_3$ / MeOD 4:1)

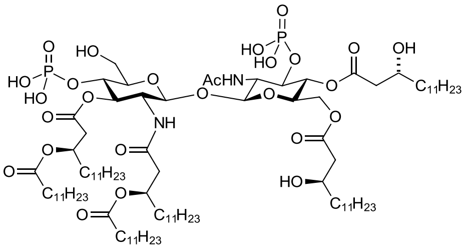

***ββ*-DLAM 909**

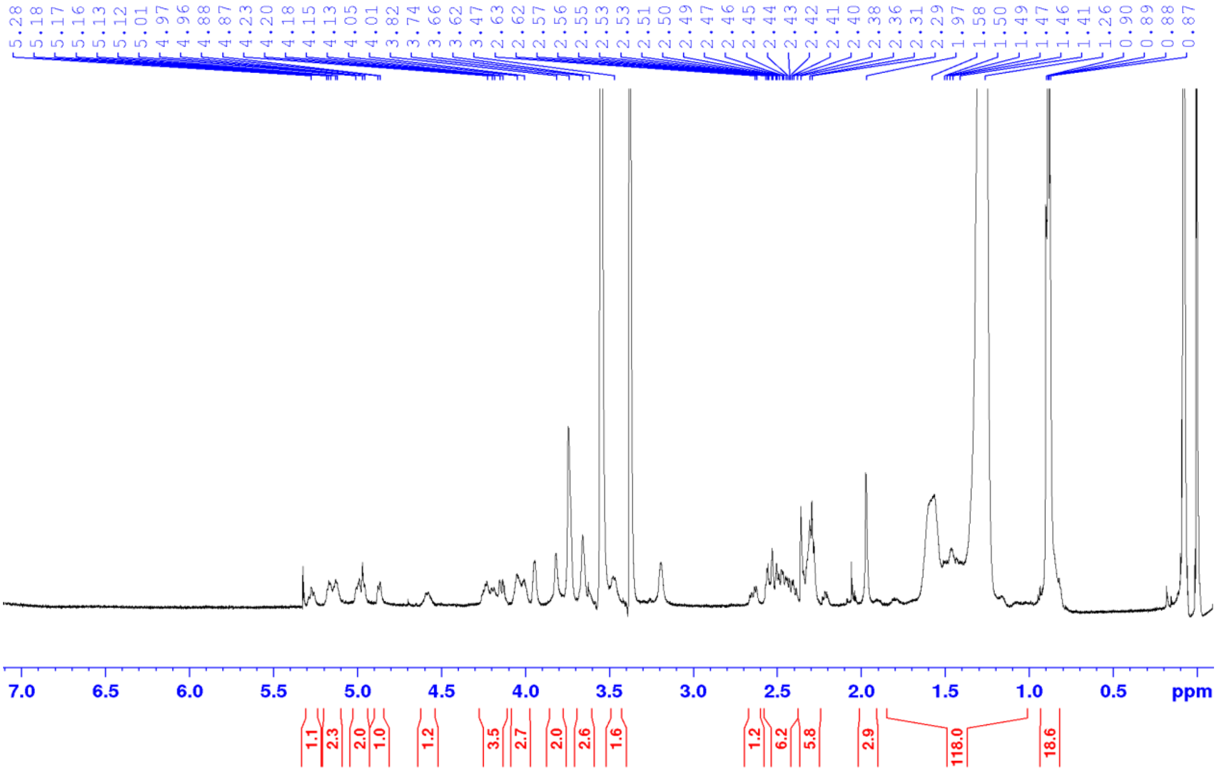

^{31}P NMR (243 MHz, $CDCl_3$ / MeOD 4:1)

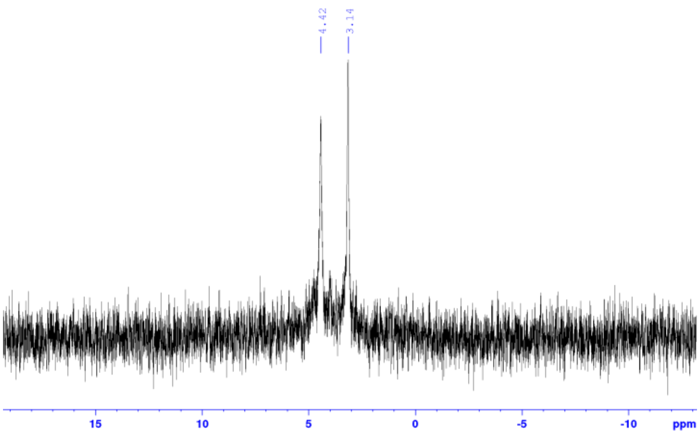

^{1}H-^{13}C HSQC (600/151 MHz, $CDCl_3$ / MeOD 4:1)

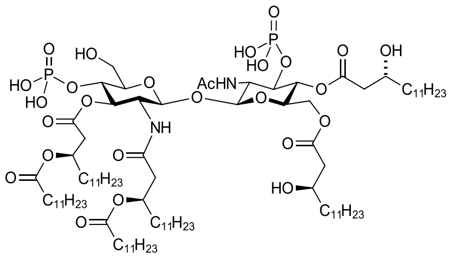

***ββ*-DLAM 909**

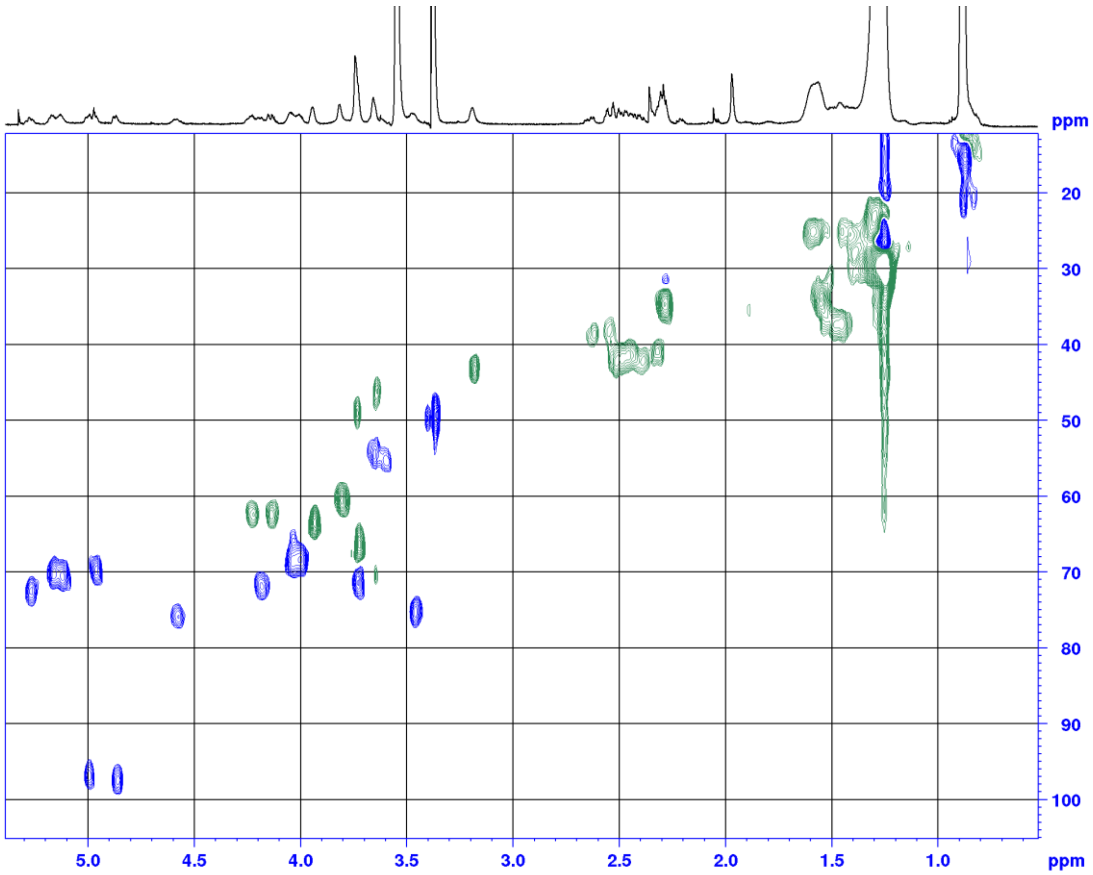

^{1}H NMR (600 MHz, $CDCl_3$)

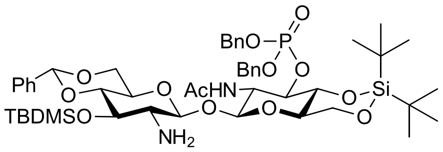

16

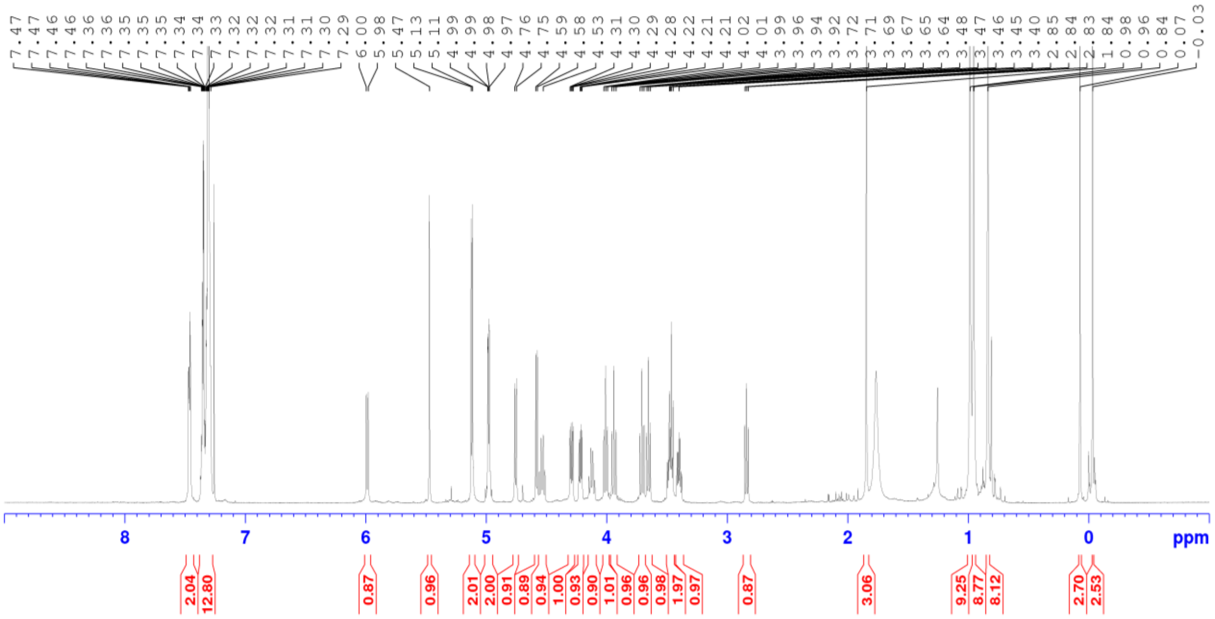

^{13}C NMR (151 MHz, $CDCl_3$)

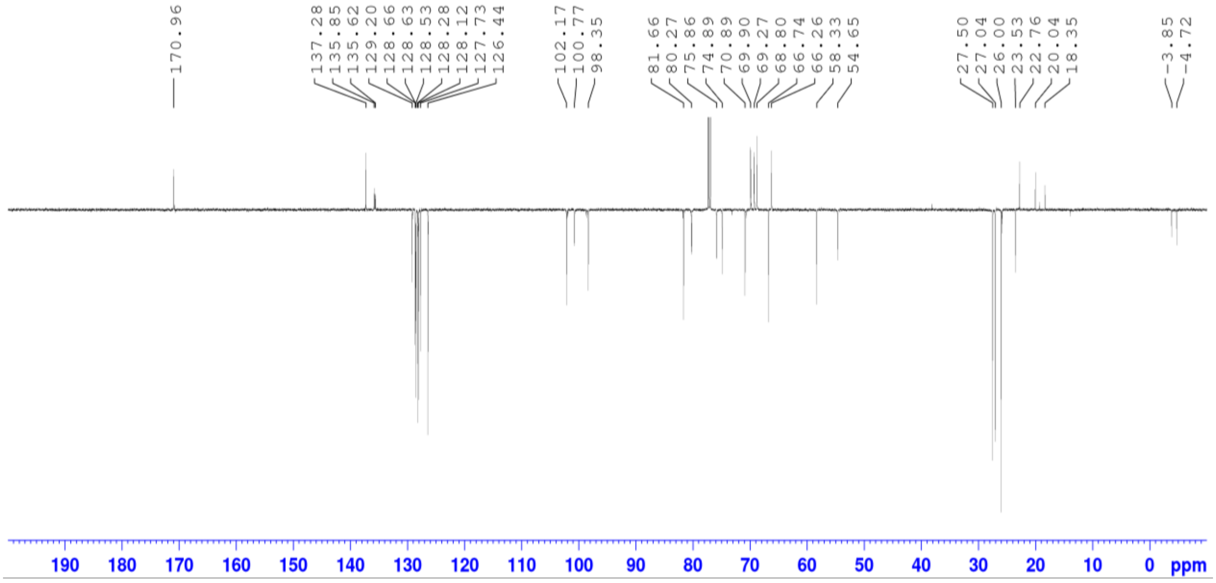

^1H NMR (600 MHz, $CDCl_3$)

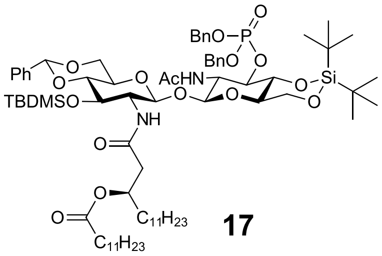

17

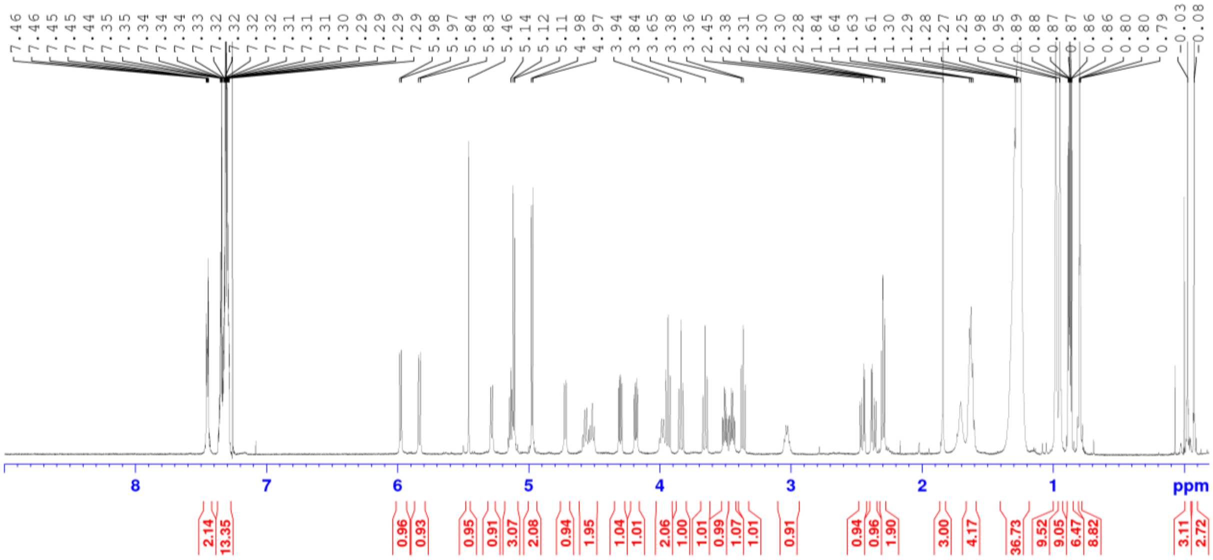

^{13}C NMR (151 MHz, $CDCl_3$)

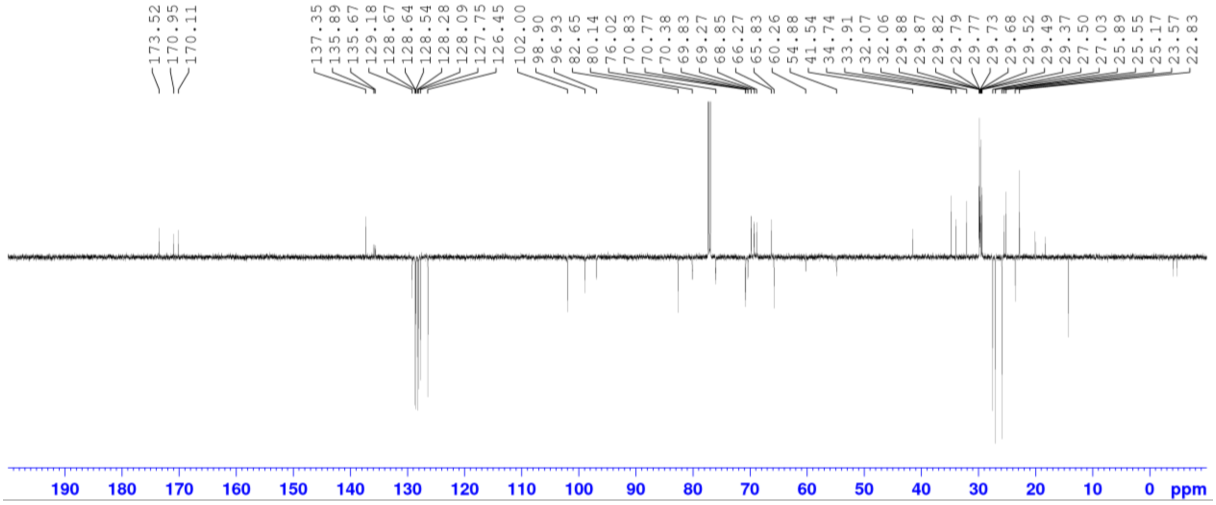

^{1}H NMR (600 MHz, $CDCl_3$)

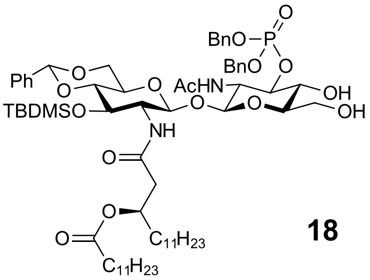

18

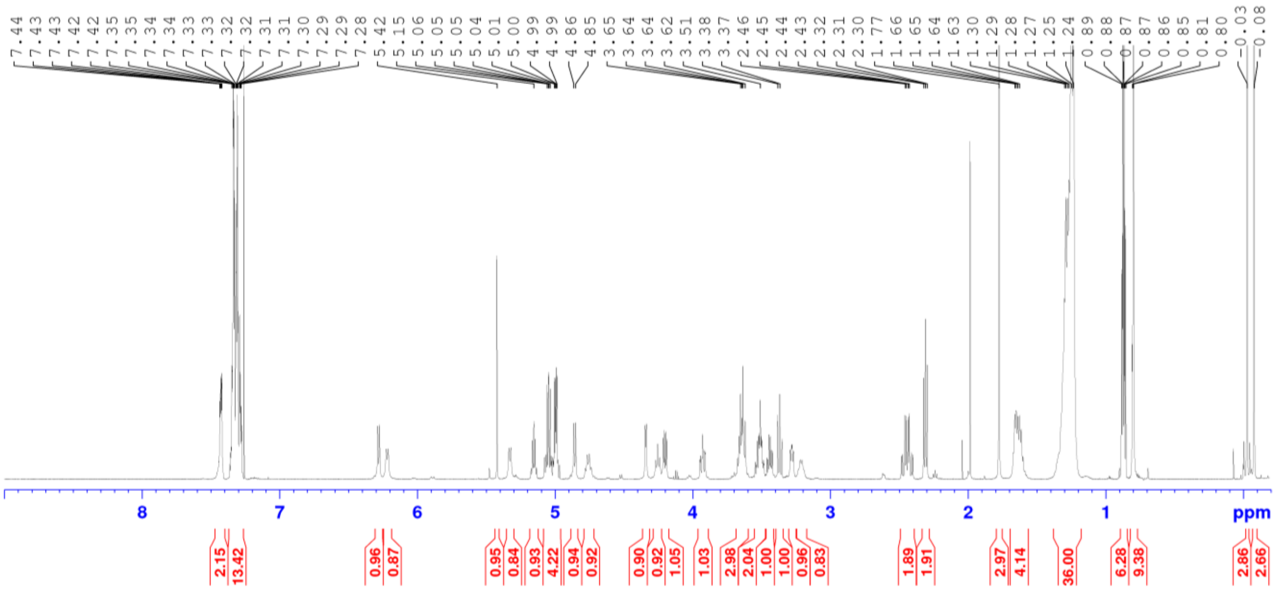

^{13}C NMR (151 MHz, $CDCl_3$)

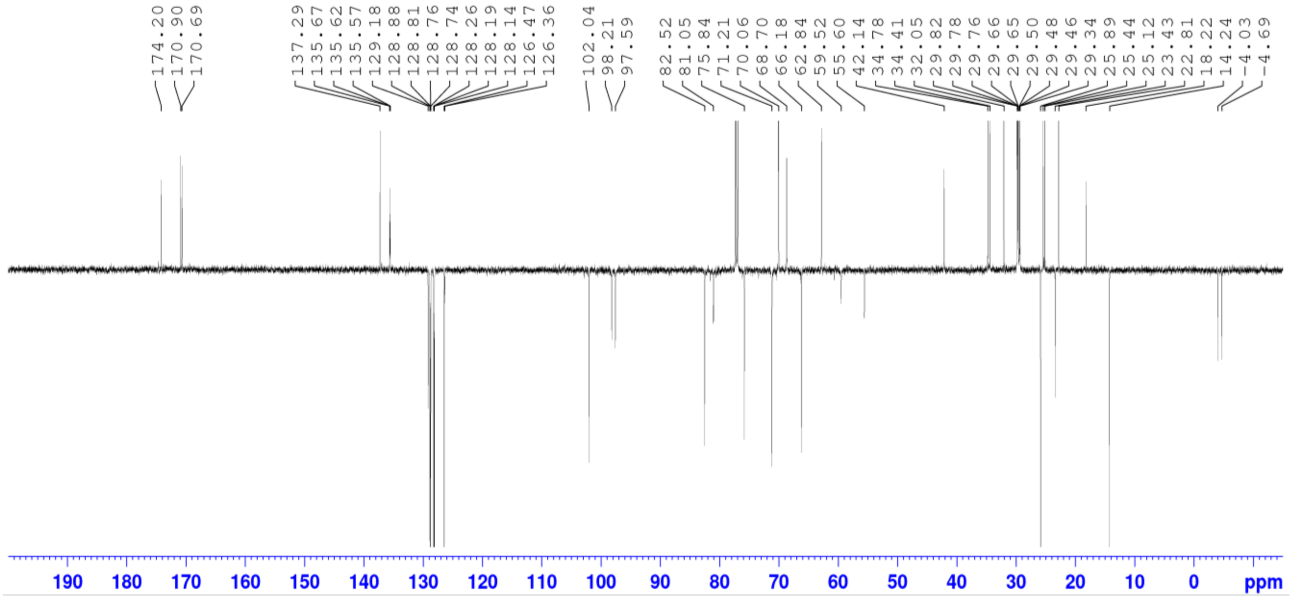

^{1}H NMR (600 MHz, $CDCl_3$)

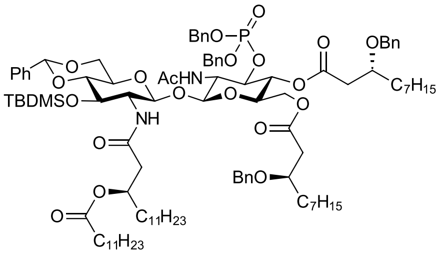

19

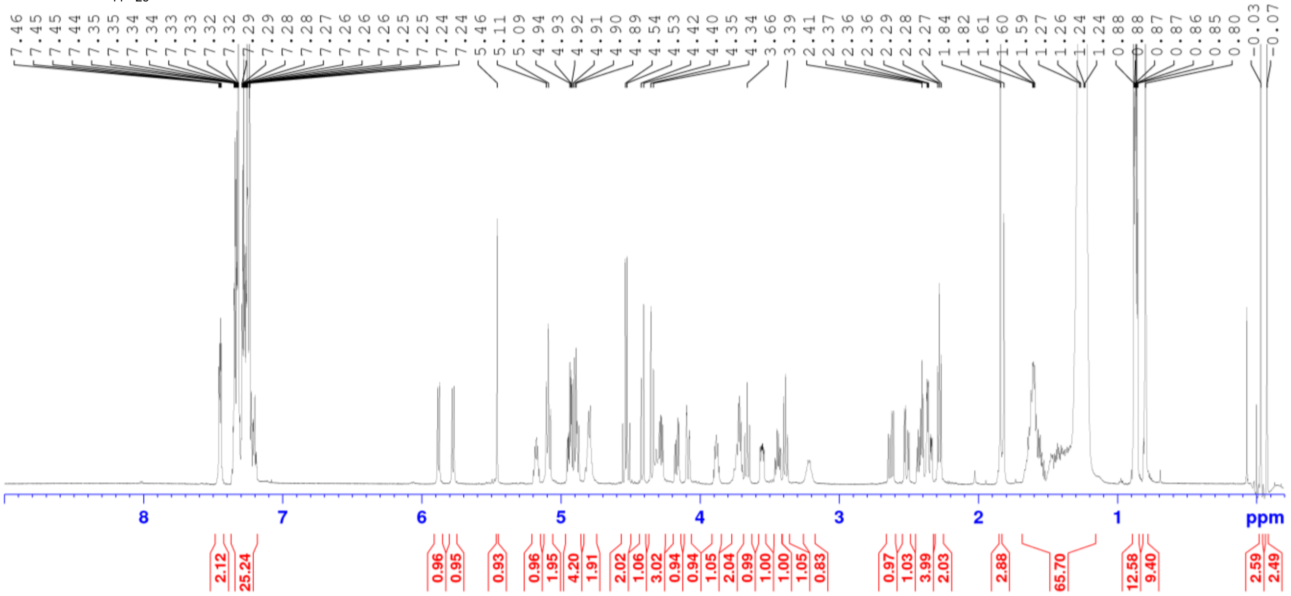

^{13}C NMR (151 MHz, $CDCl_3$)

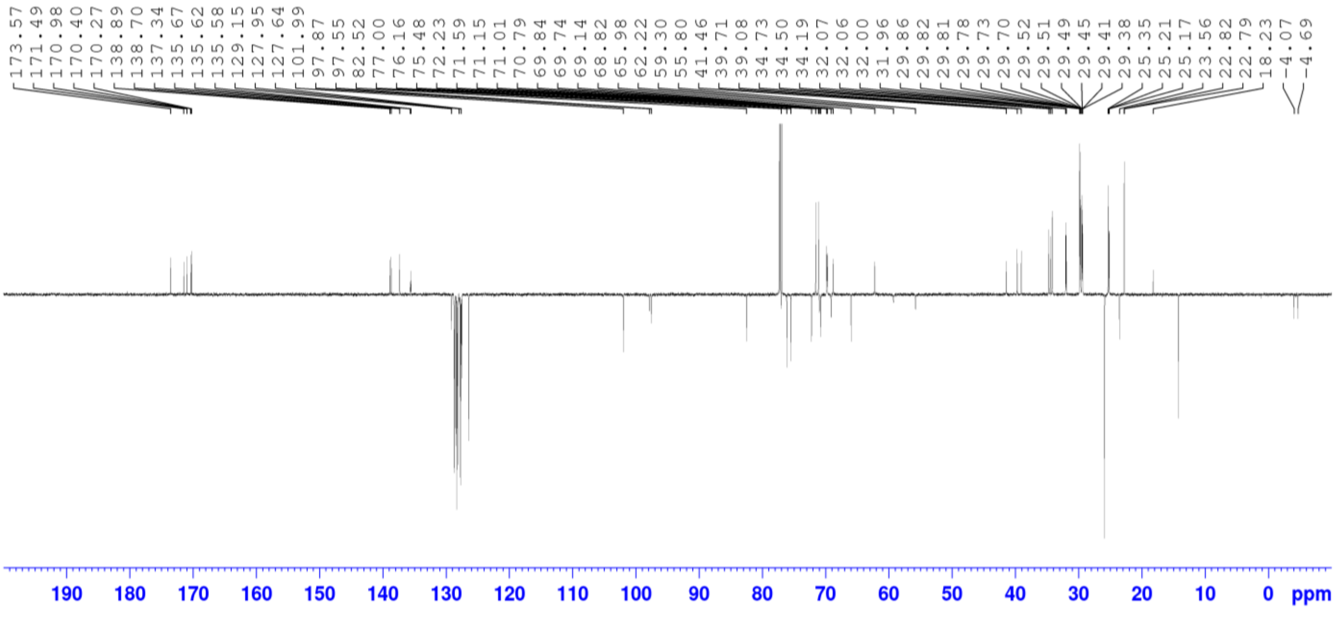

^{1}H NMR (600 MHz, $CDCl_3$)

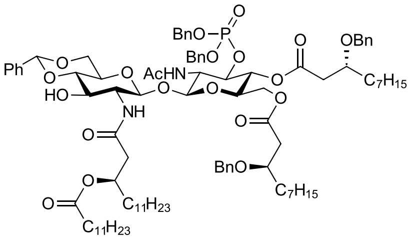

20

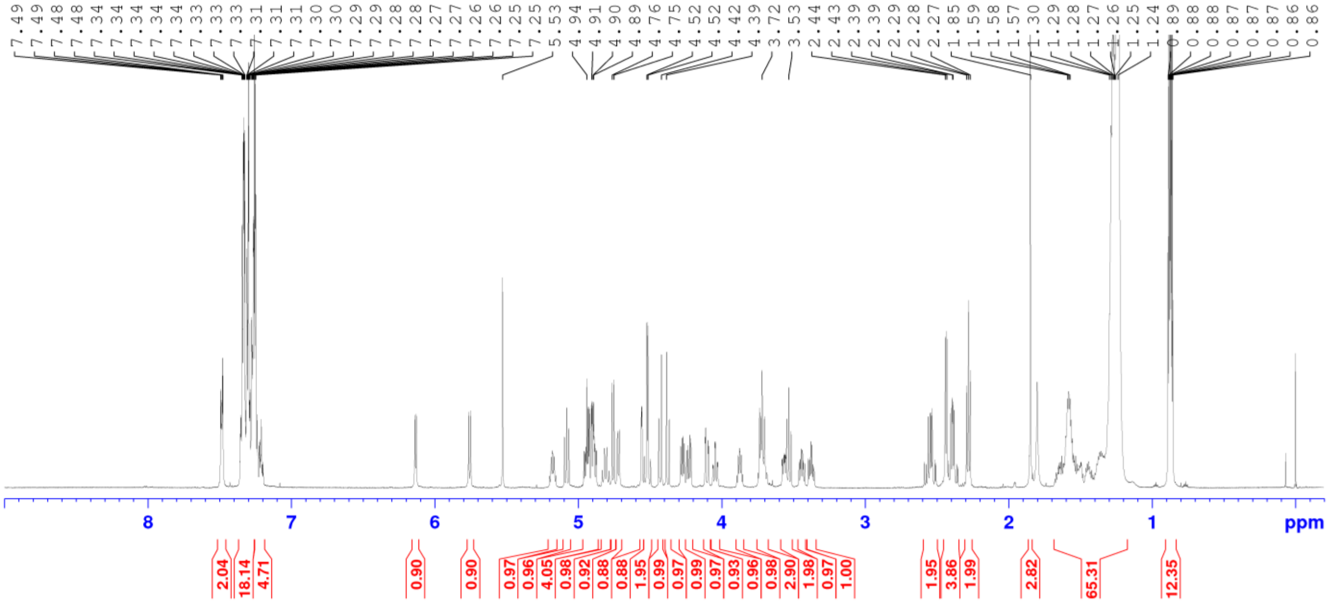

^{13}C NMR (151 MHz, $CDCl_3$)

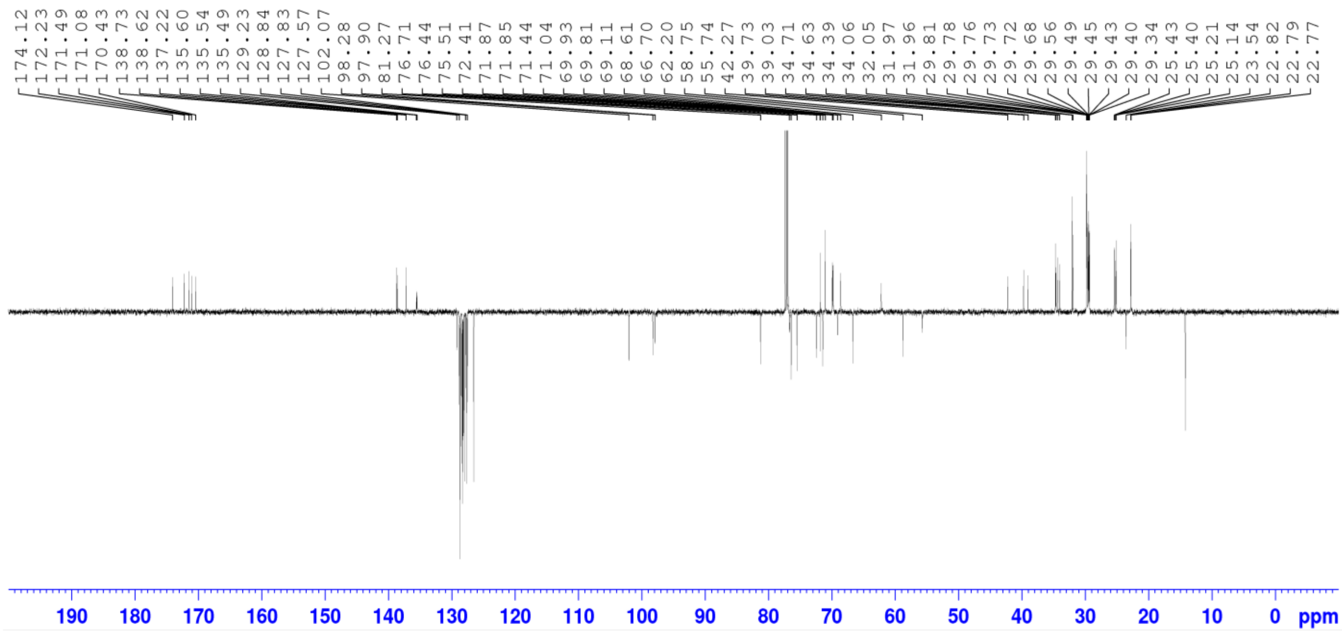

^{1}H NMR (600 MHz, $CDCl_3$)

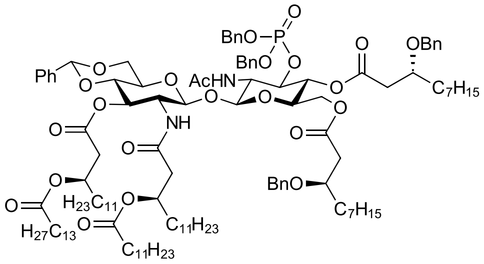

21

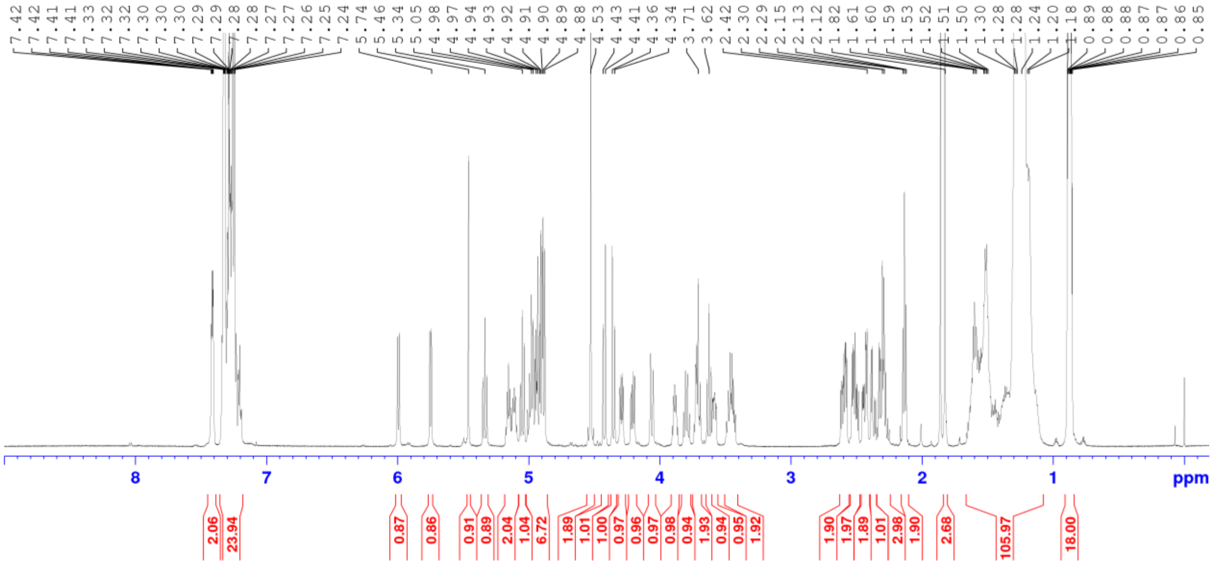

^{13}C NMR (151 MHz, $CDCl_3$)

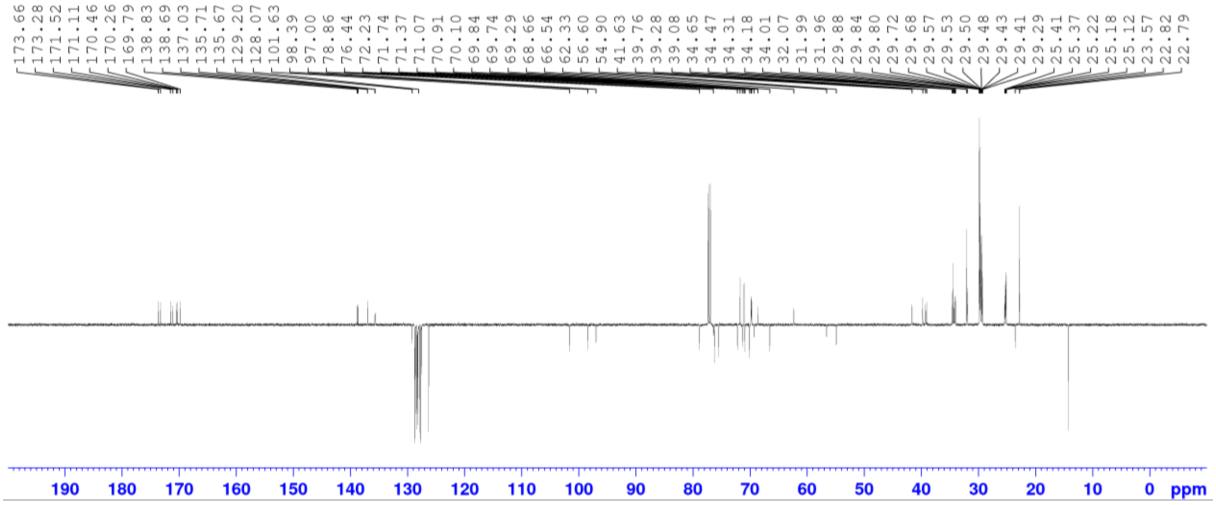

^{1}H NMR (600 MHz, $CDCl_3$)

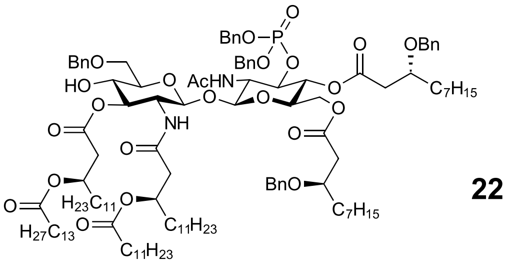

22

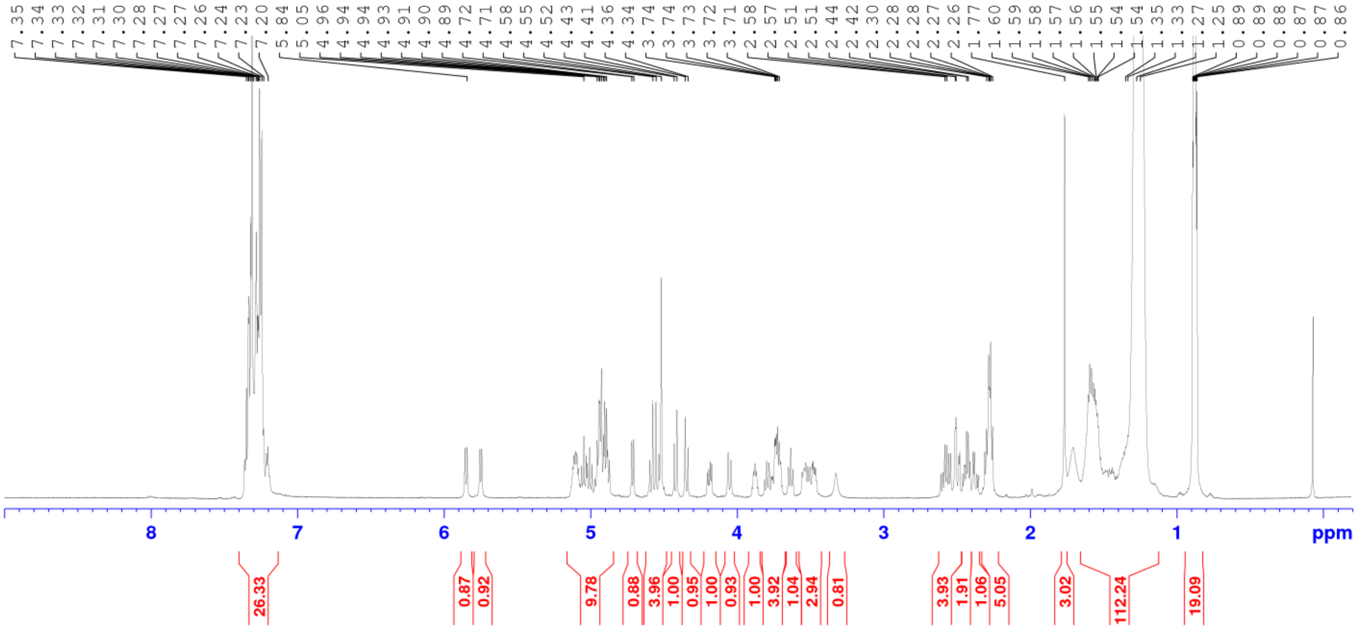

^{13}C NMR (151 MHz, $CDCl_3$)

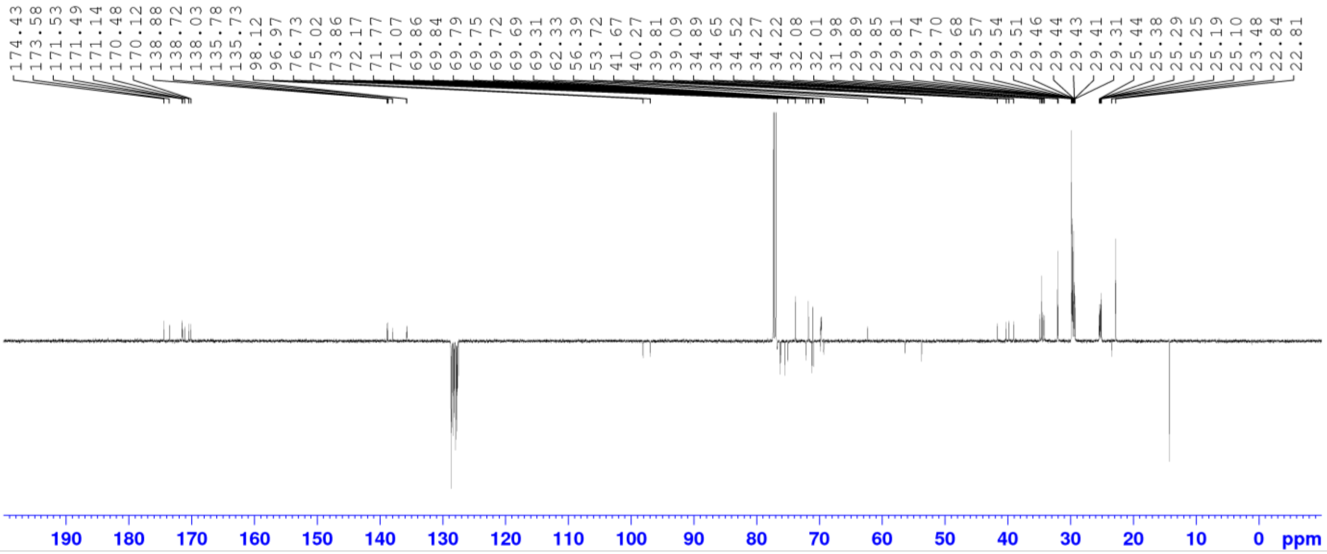

^{1}H NMR (600 MHz, $CDCl_3$)

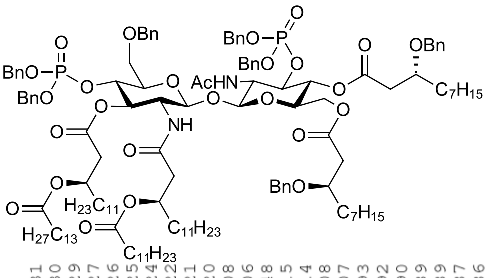

23

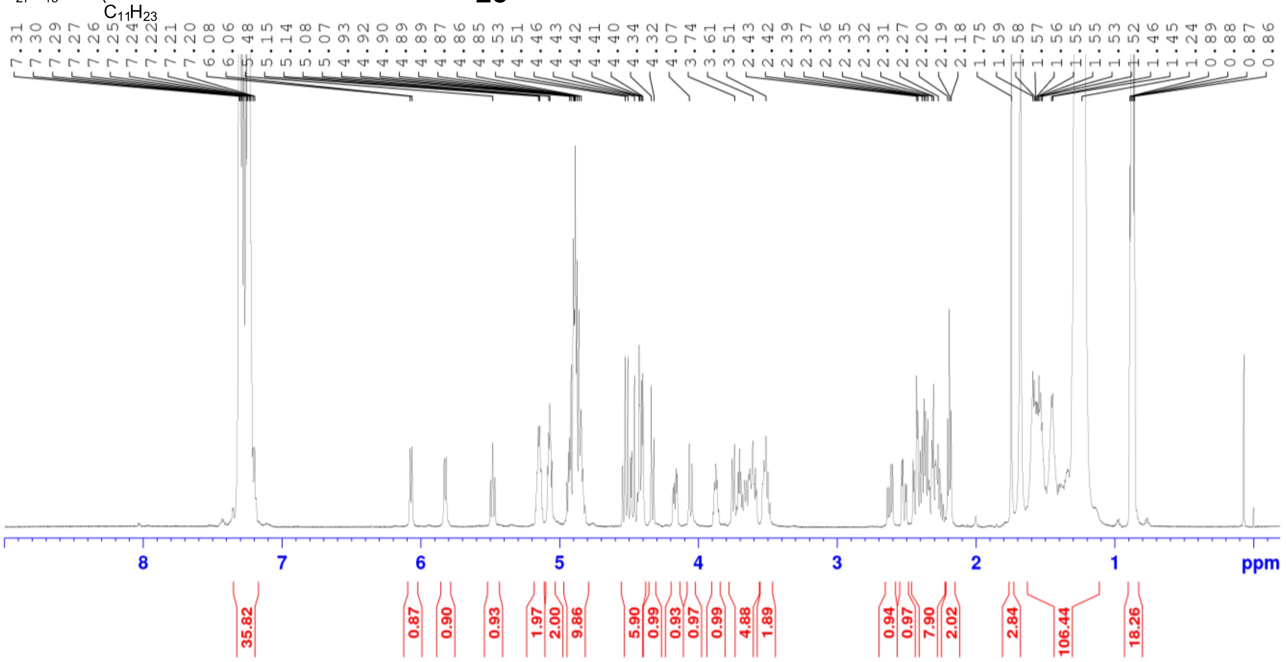

^{13}C NMR (151 MHz, $CDCl_3$)

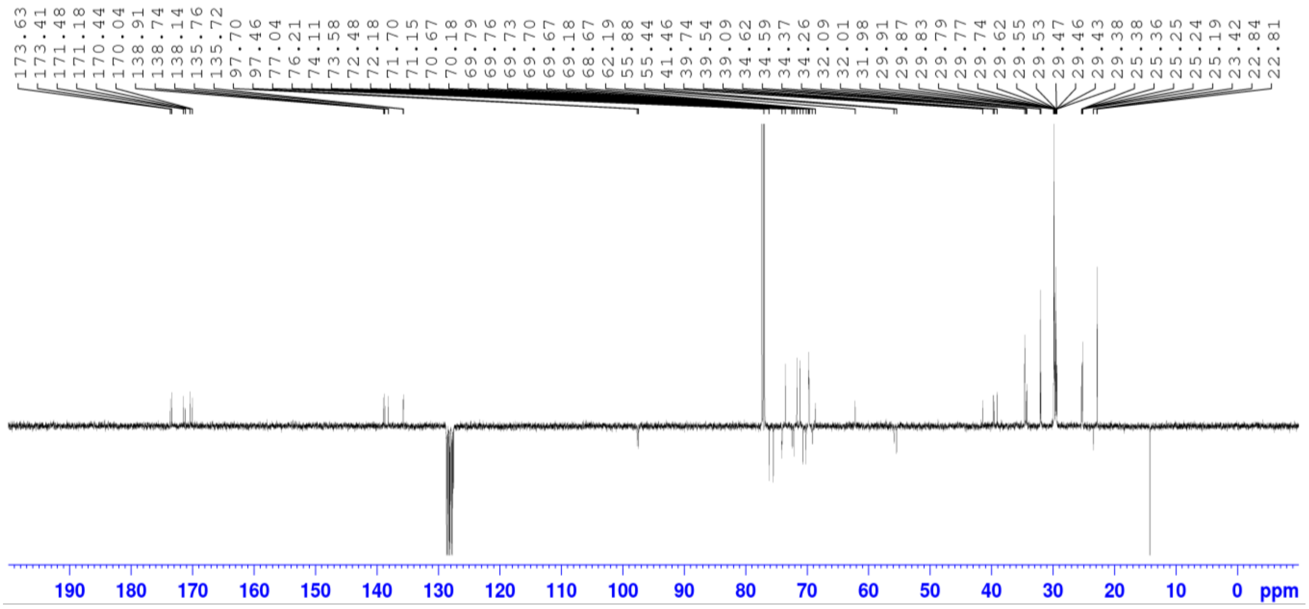

^{1}H NMR (600 MHz, $CDCl_3$ / MeOD 5:3, water suppression)

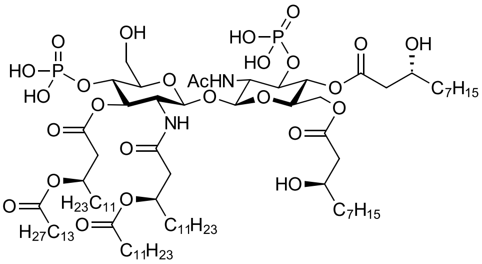

***ββ*-DLAM 919**

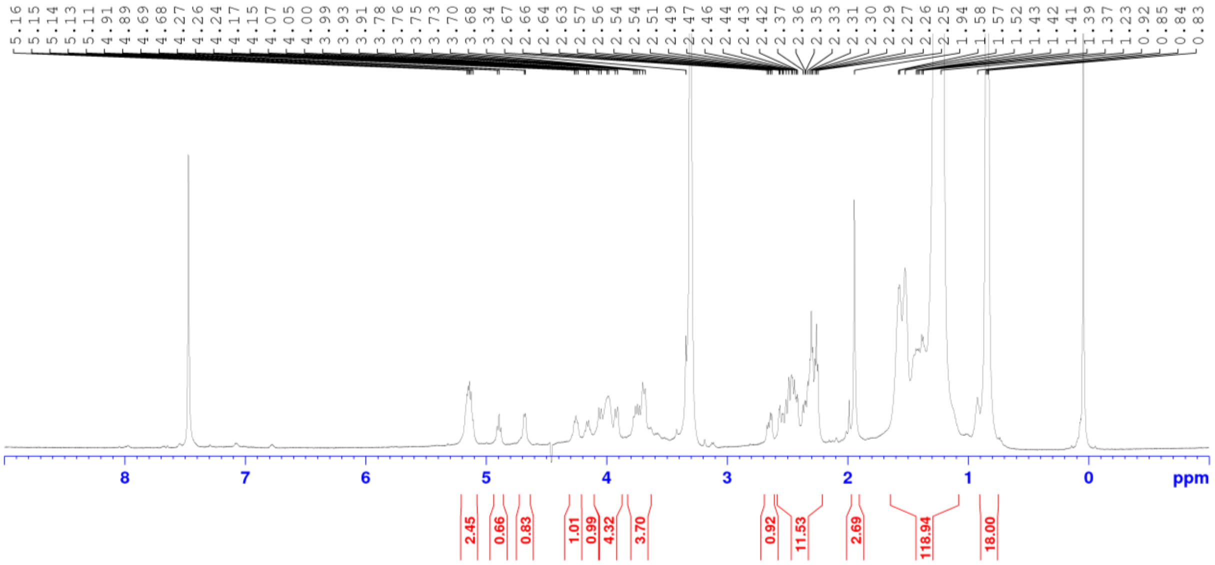

^{1}H-^{31}P HMBC (600/243 MHz, $CDCl_3$ / MeOD 5:3)

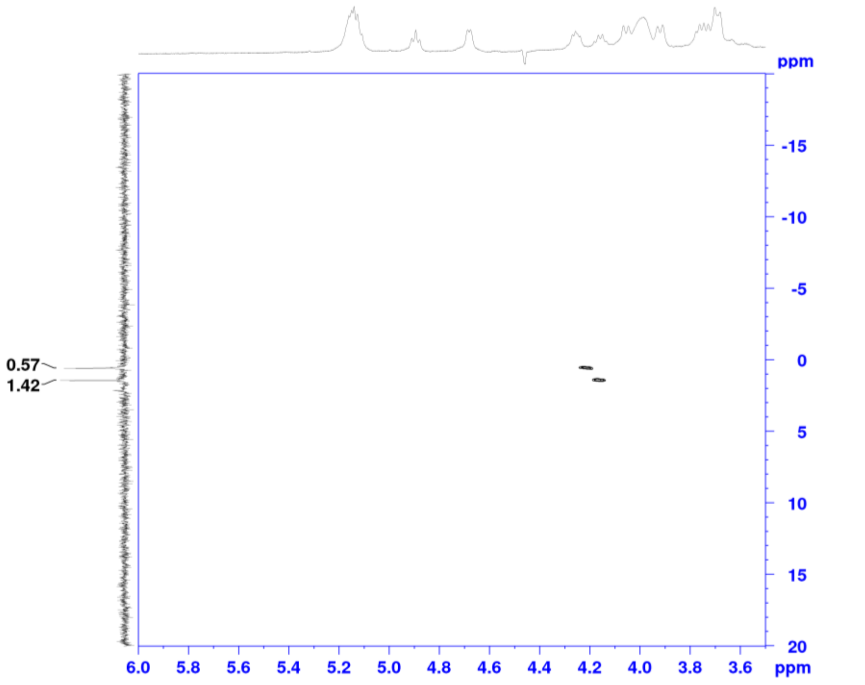

^1H-^{13}C HSQC (600/151 MHz, $CDCl_3$ / MeOD 5:3)

***ββ*-DLAM 919**

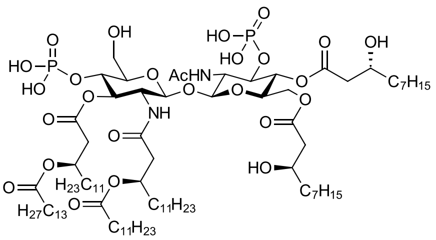

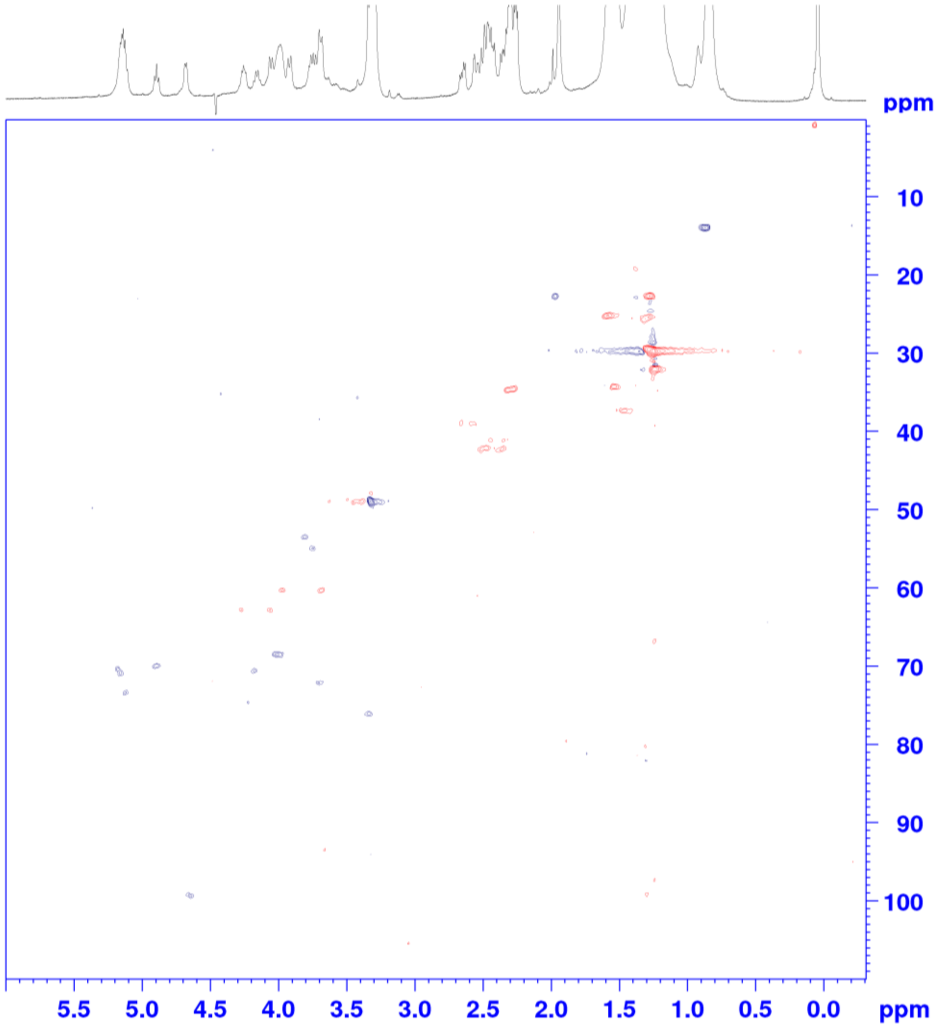

^{1}H NMR (600 MHz, $CDCl_3$)

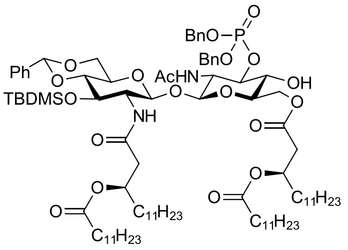

24

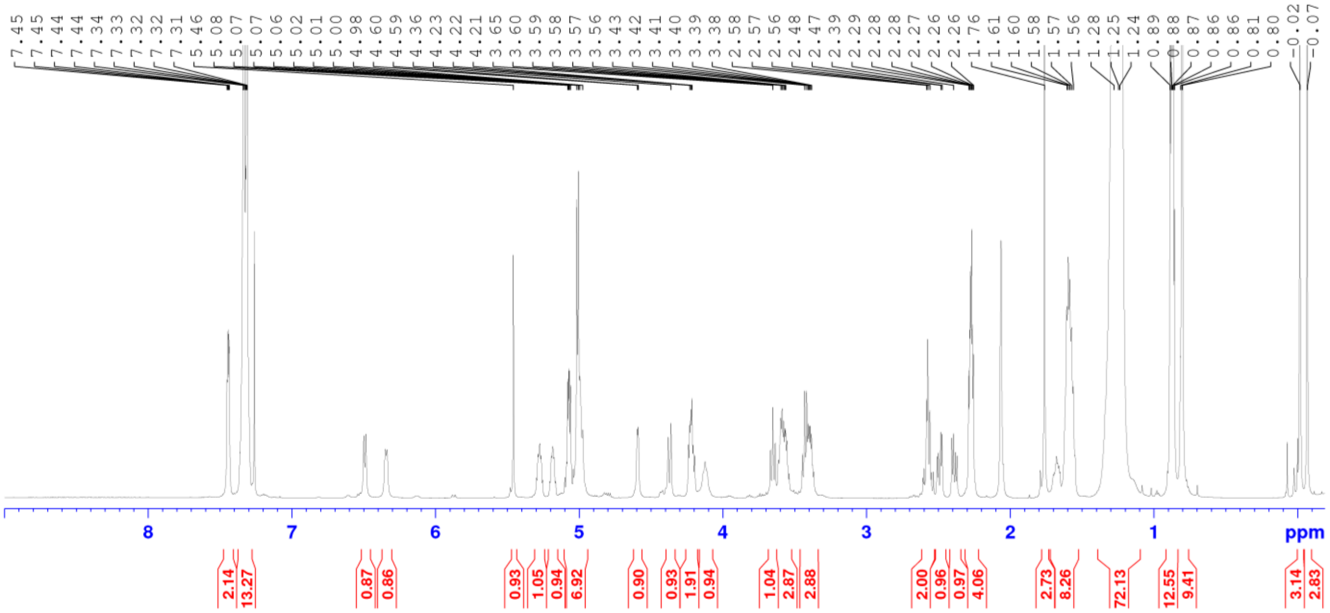

^{13}C NMR (151 MHz, $CDCl_3$)

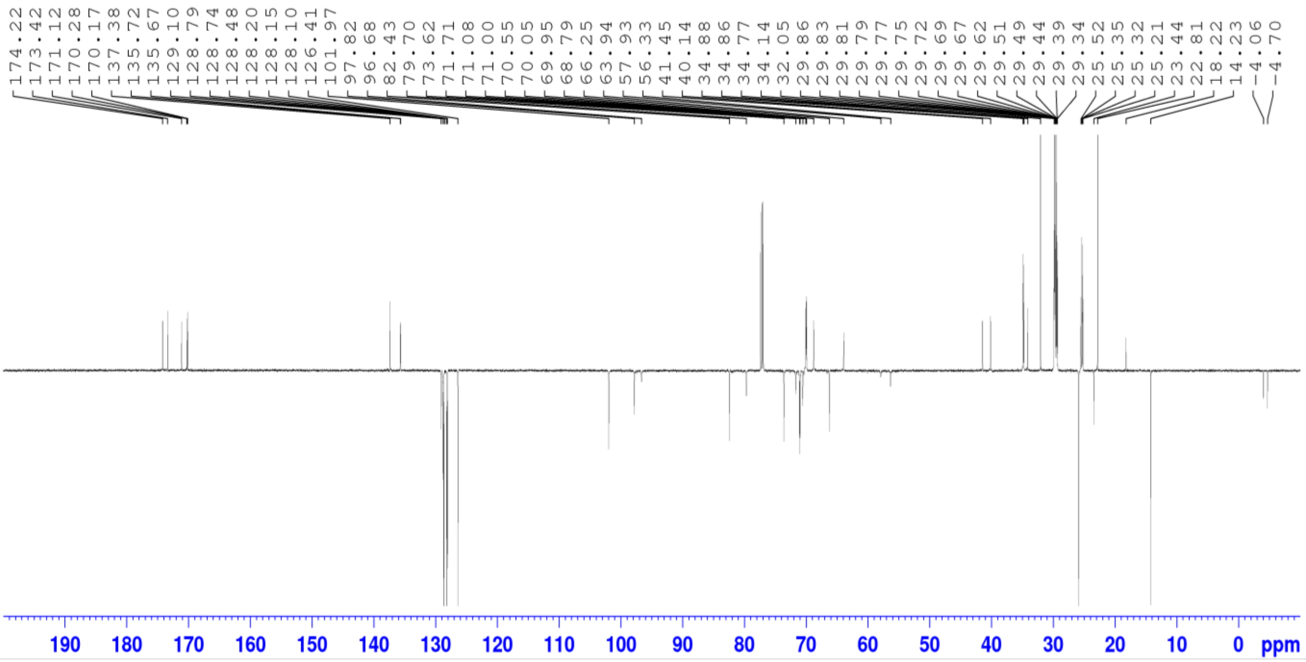

^{1}H NMR (600 MHz, $CDCl_3$)

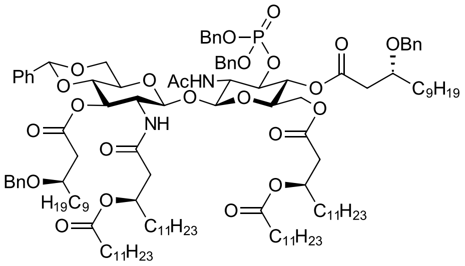

25

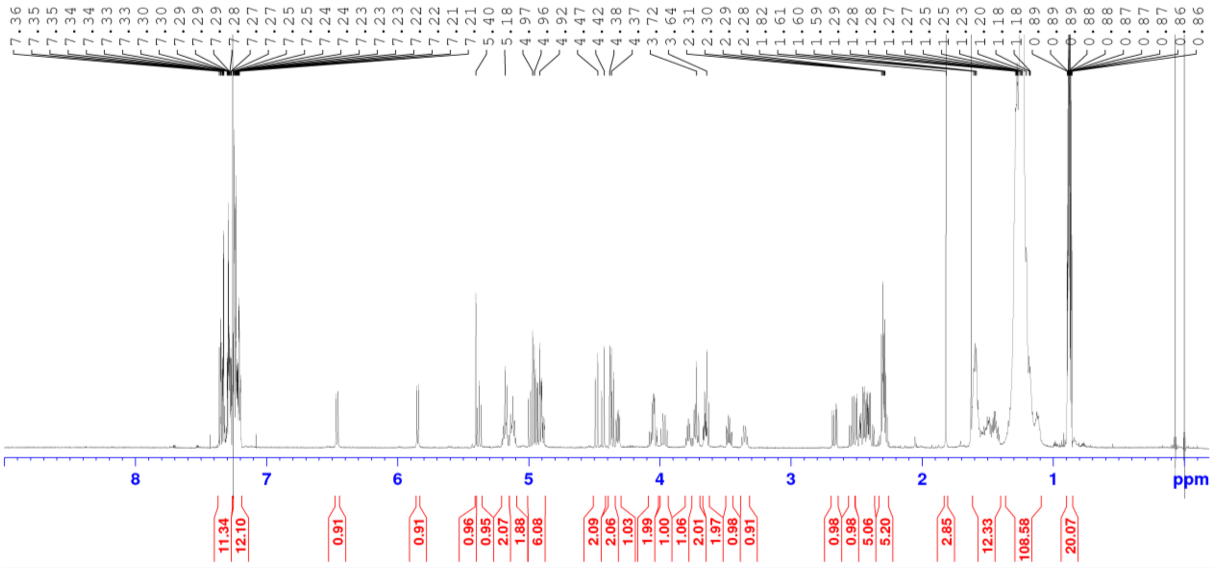

^{13}C NMR (151 MHz, $CDCl_3$)

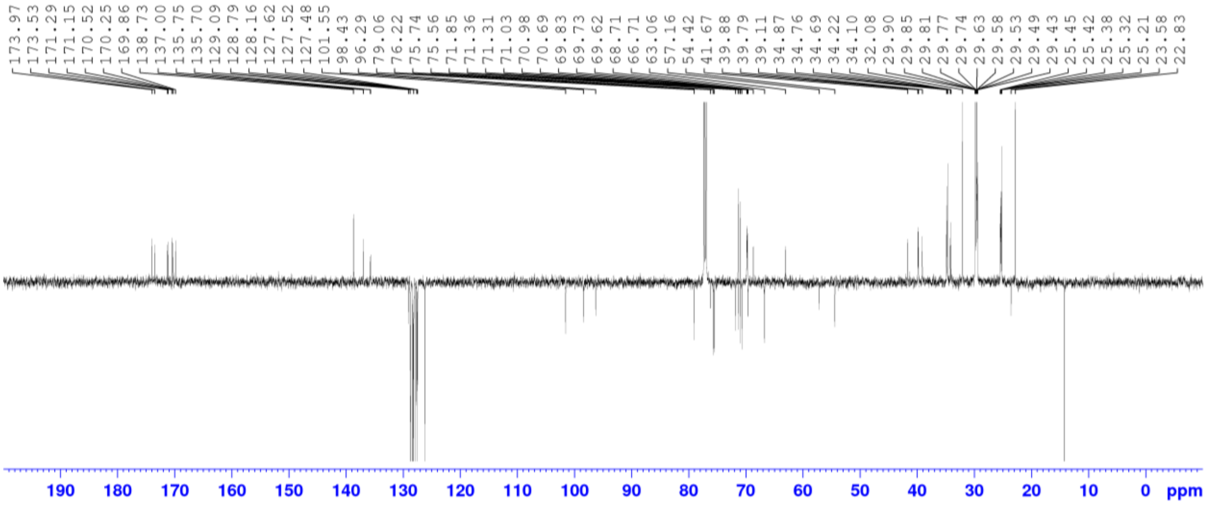

^{1}H NMR (600 MHz, $CDCl_3$)

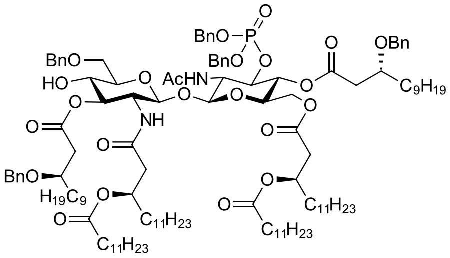

26

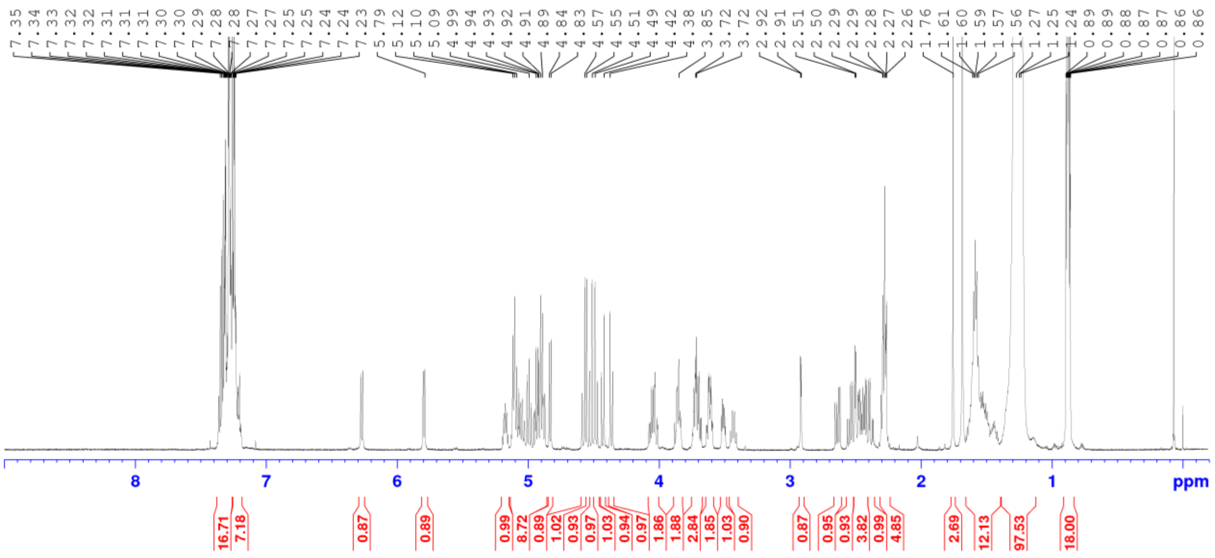

^{13}C NMR (151 MHz, $CDCl_3$)

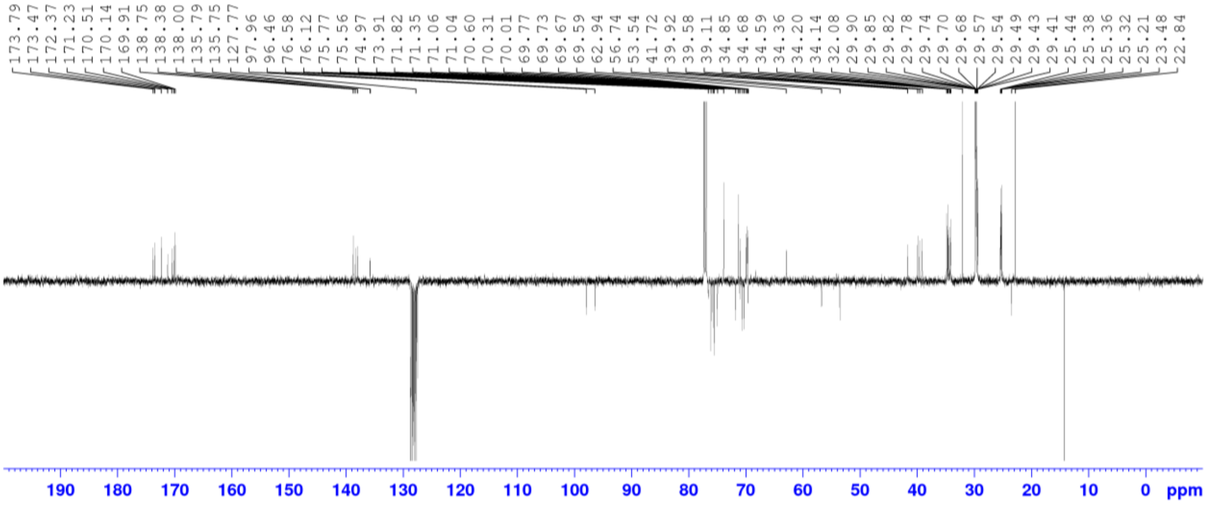

^{1}H NMR (600 MHz, $CDCl_3$)

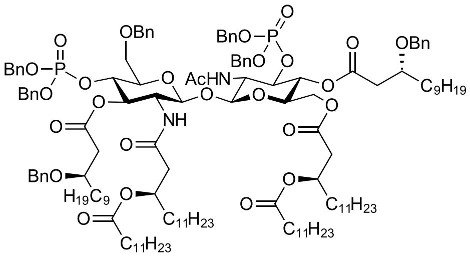

27

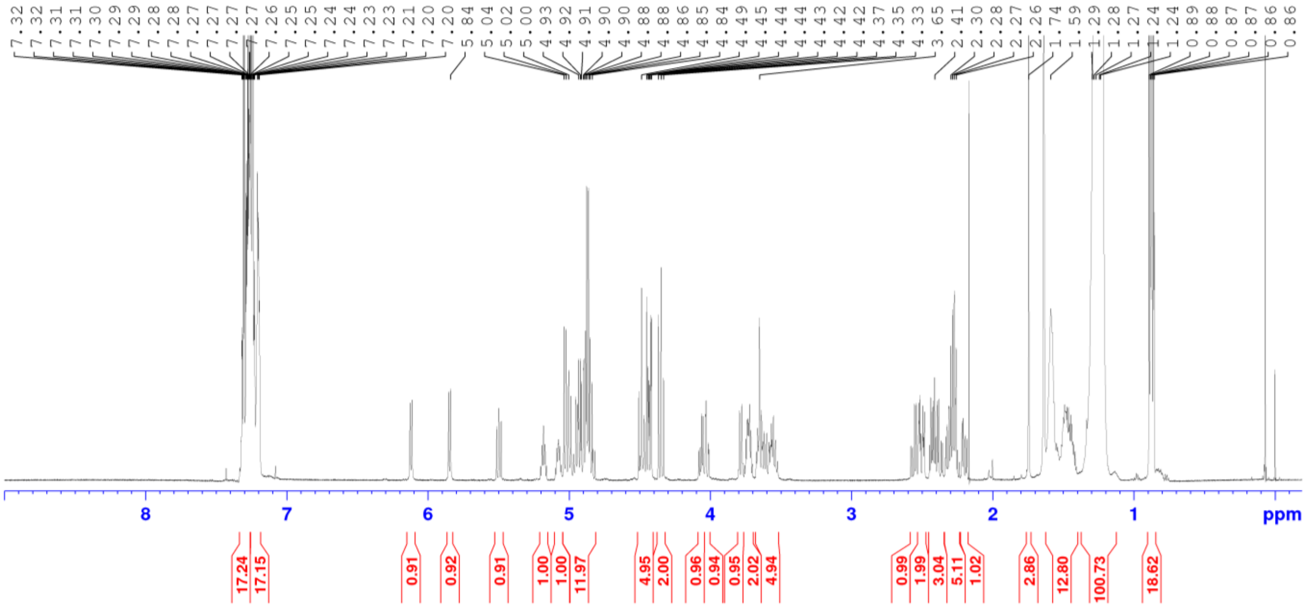

^{13}C NMR (151 MHz, $CDCl_3$)

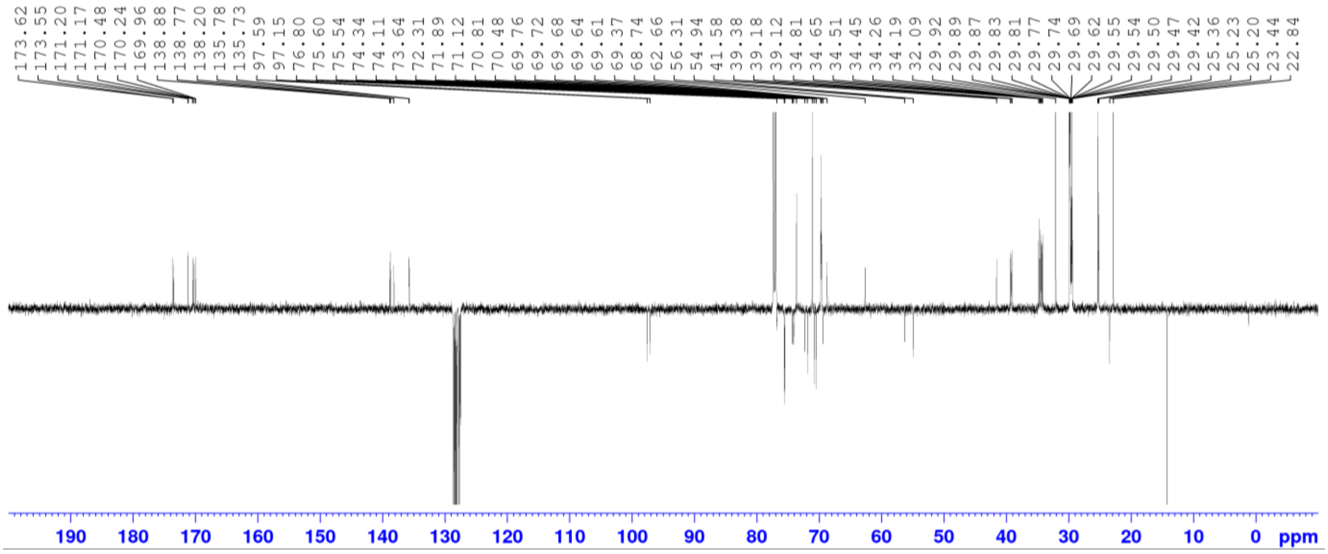

^{1}H NMR (600 MHz, $CDCl_3$ / MeOD 5:3)

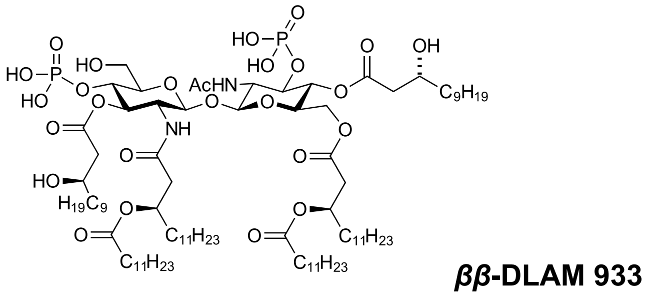

***ββ*-DLAM 933**

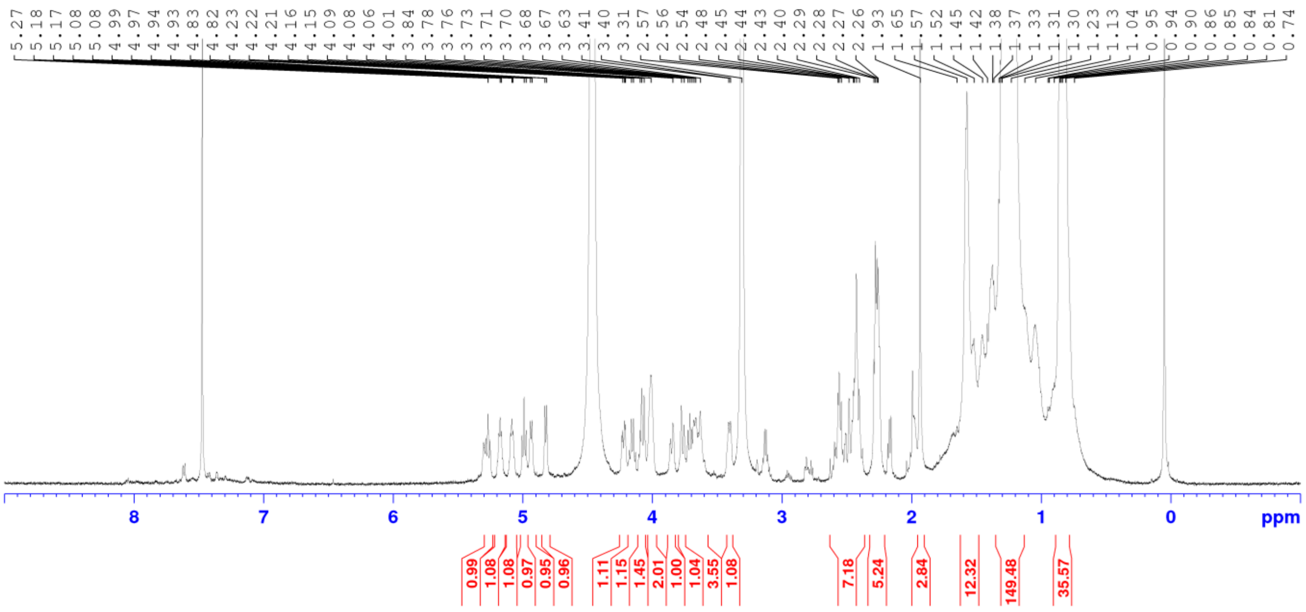

^{1}H-^{31}P HMBC (600/243 MHz, $CDCl_3$ / MeOD 5:3)

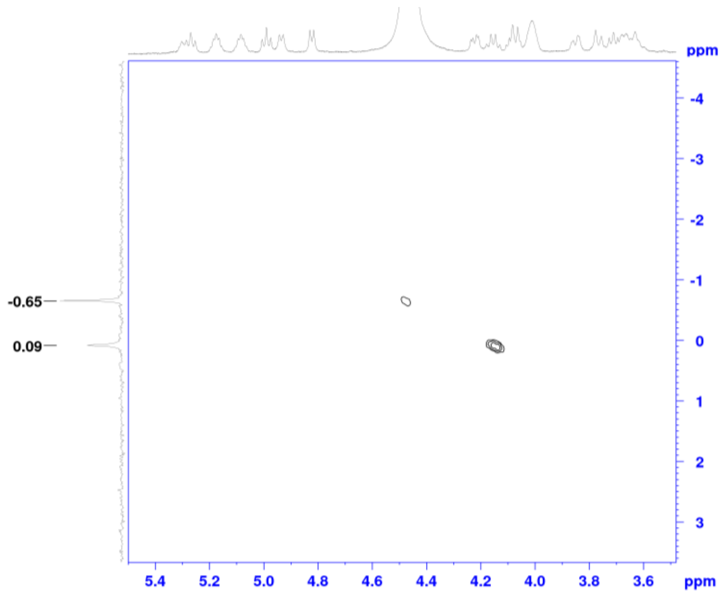

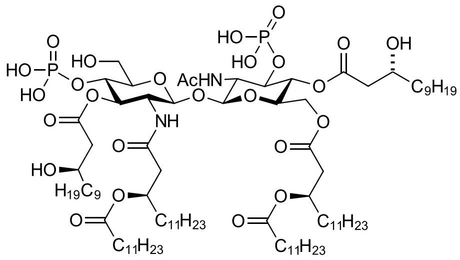

^1H-^{13}C HSQC (600/151 MHz, $CDCl_3$ / MeOD 5:3)

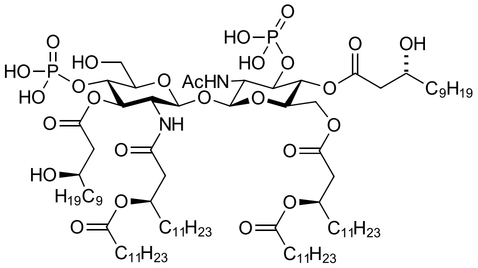

***ββ*-DLAM 933**

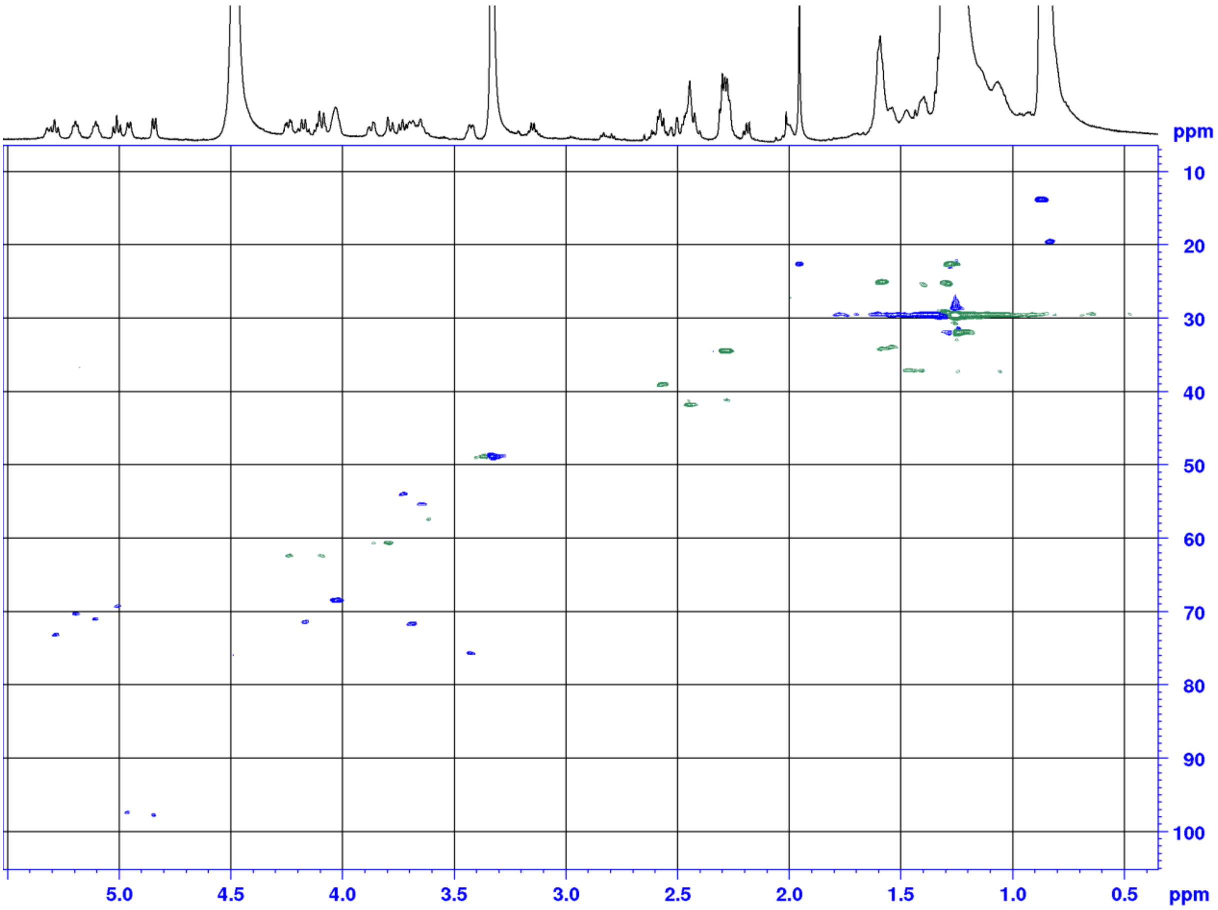

^{1}H NMR (600 MHz, $CDCl_3$)

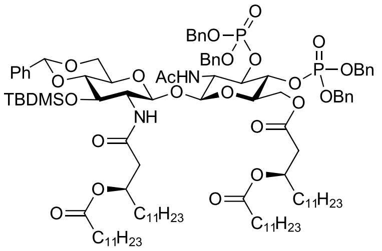

28

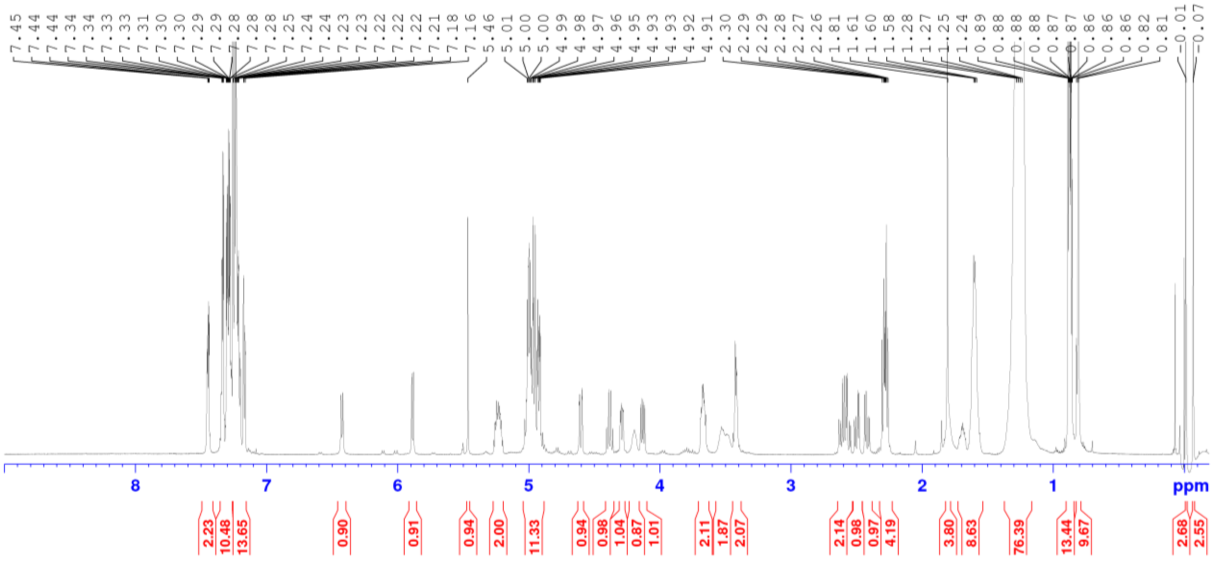

^{13}C NMR (151 MHz, $CDCl_3$)

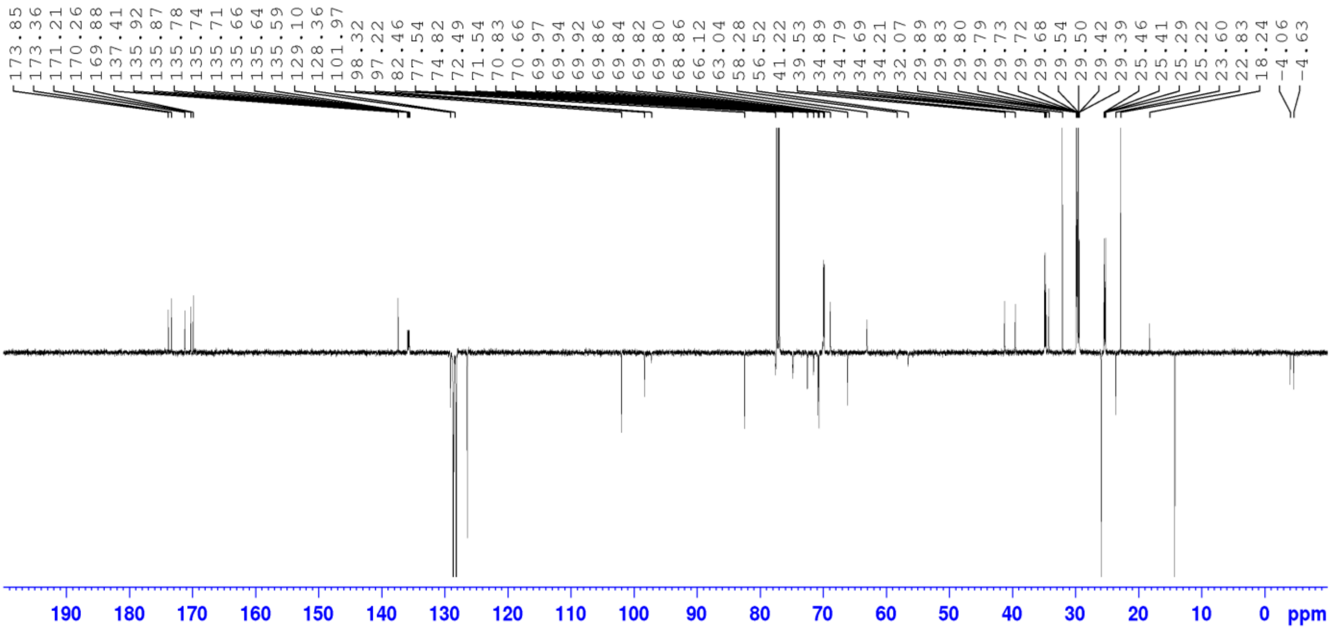

^{1}H NMR (600 MHz, $CDCl_3$)

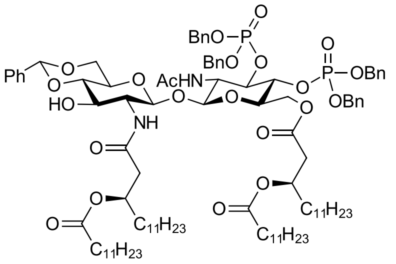

29

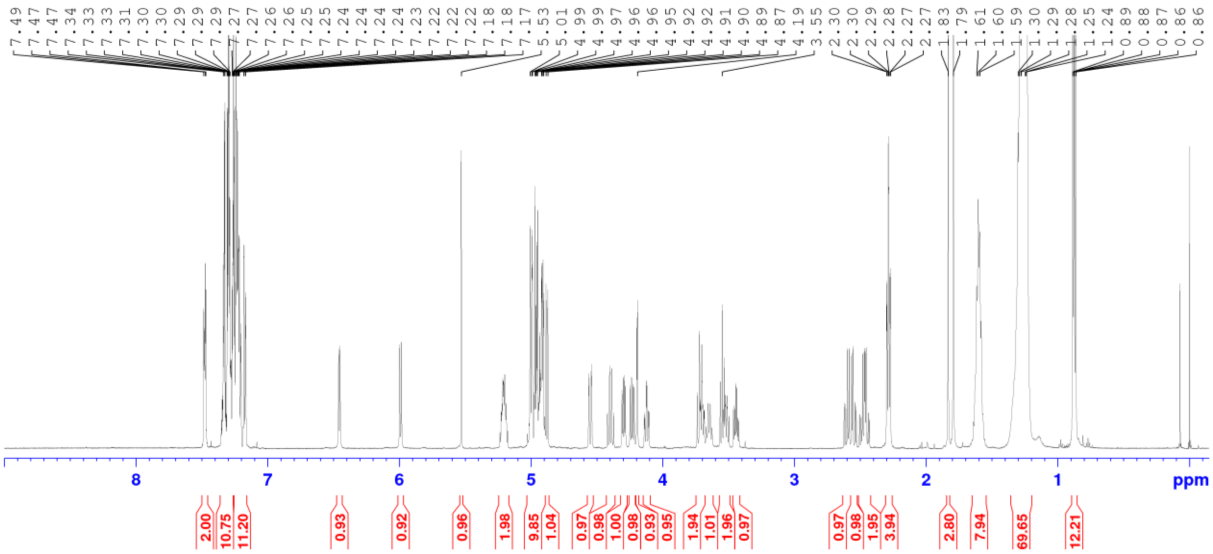

^{13}C NMR (151 MHz, $CDCl_3$)

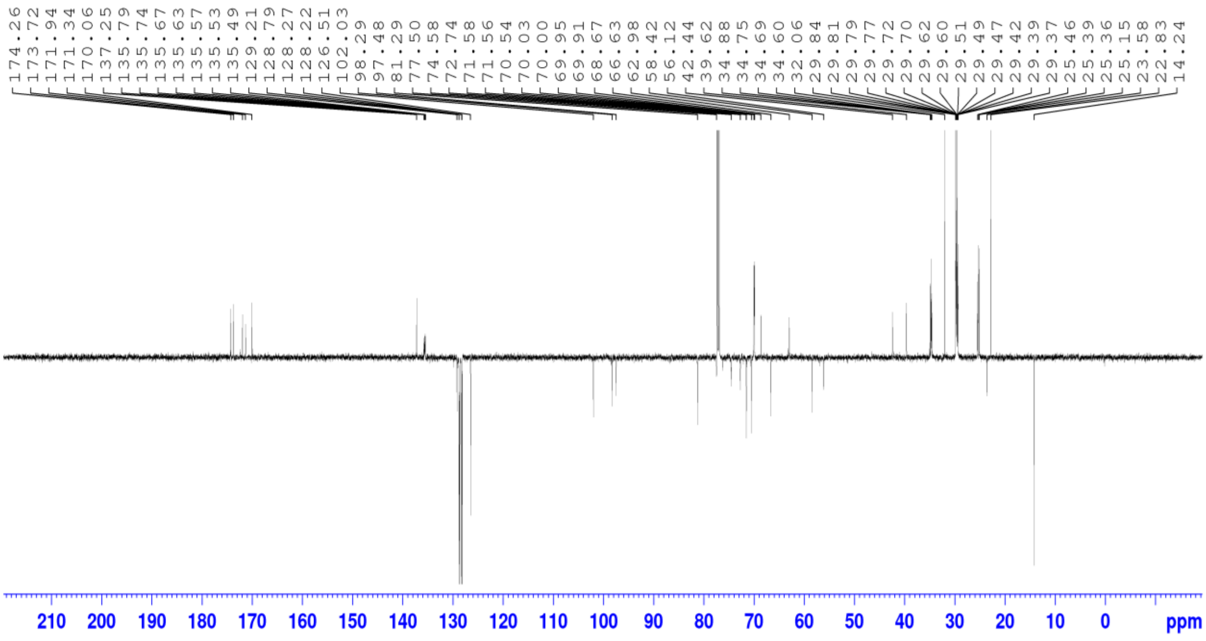

^{1}H NMR (600 MHz, $CDCl_3$)

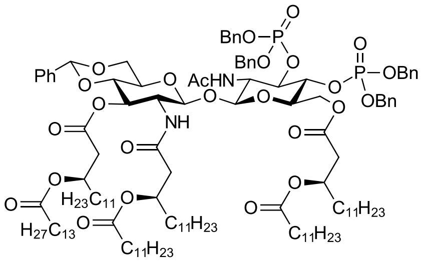

30

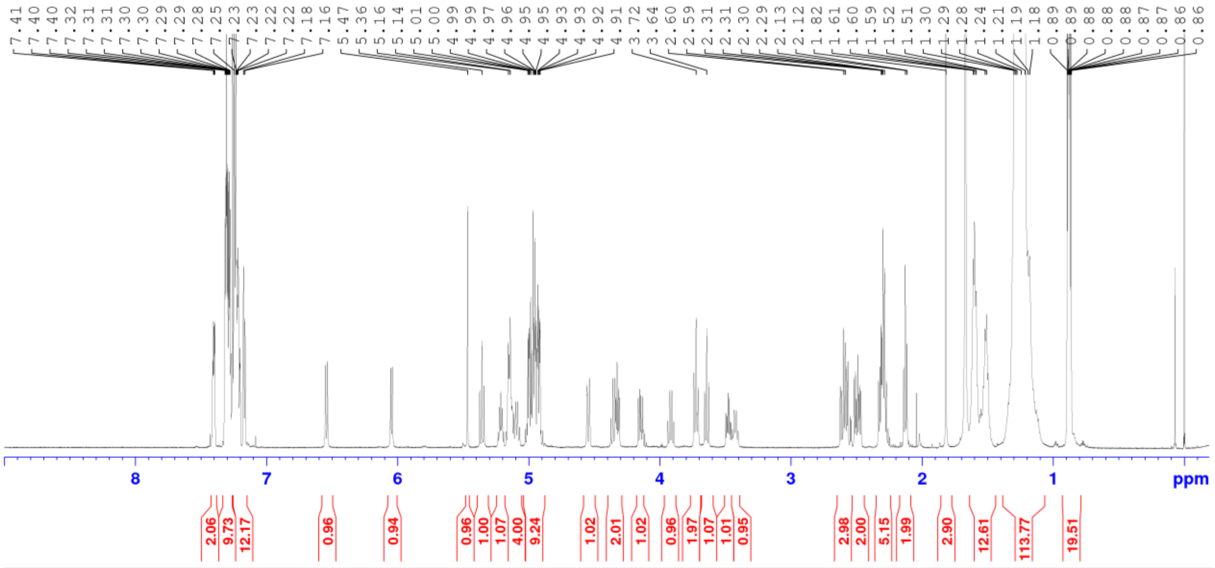

^{13}C NMR (151 MHz, $CDCl_3$)

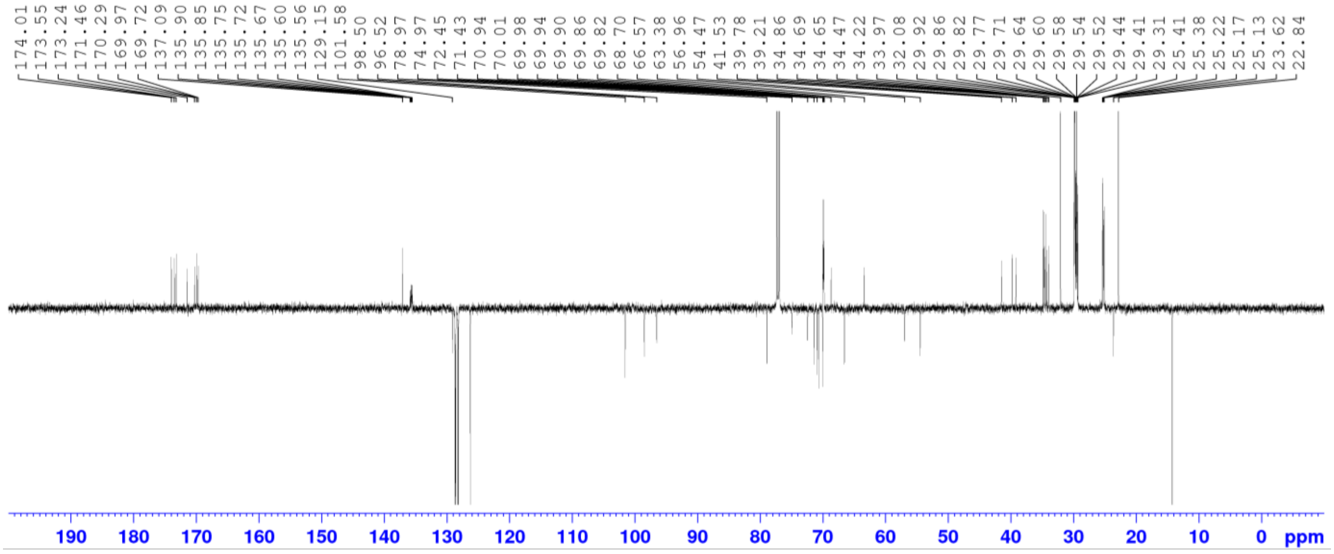

^{1}H NMR (600 MHz, $CDCl_3$)

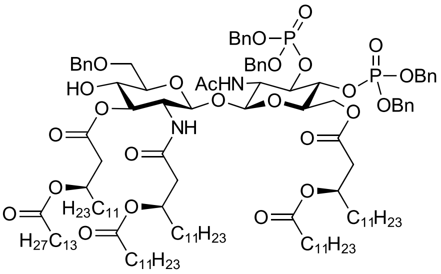

31

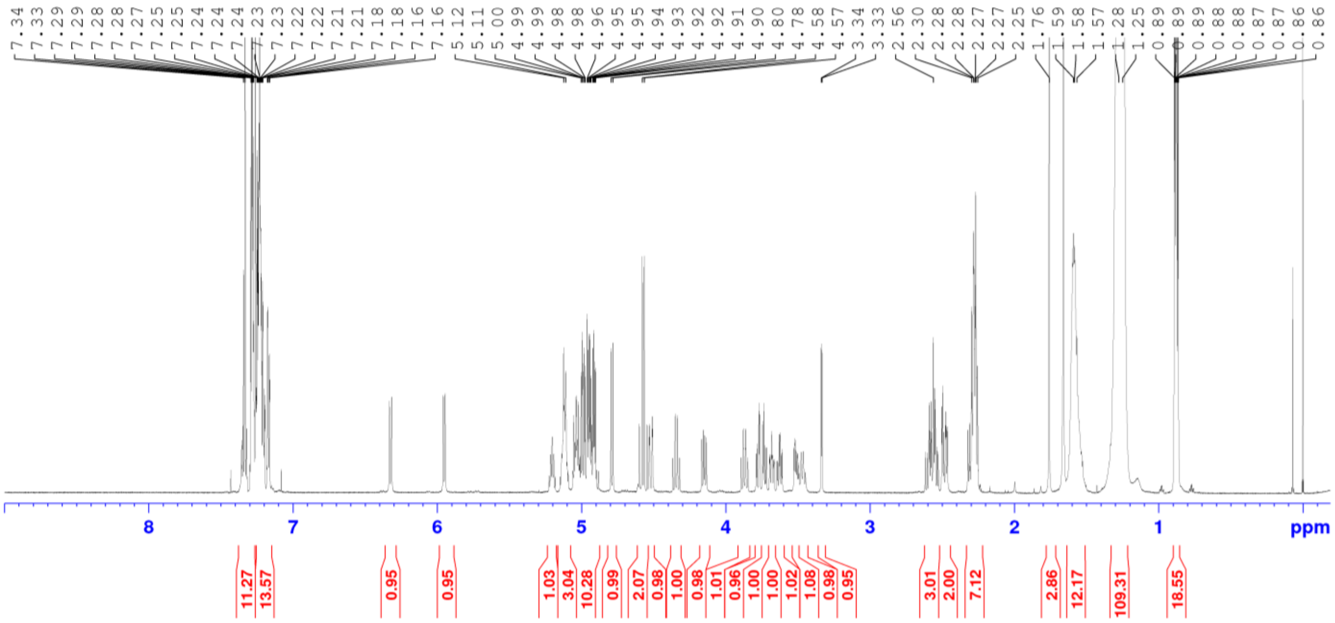

^{13}C NMR (151 MHz, $CDCl_3$)

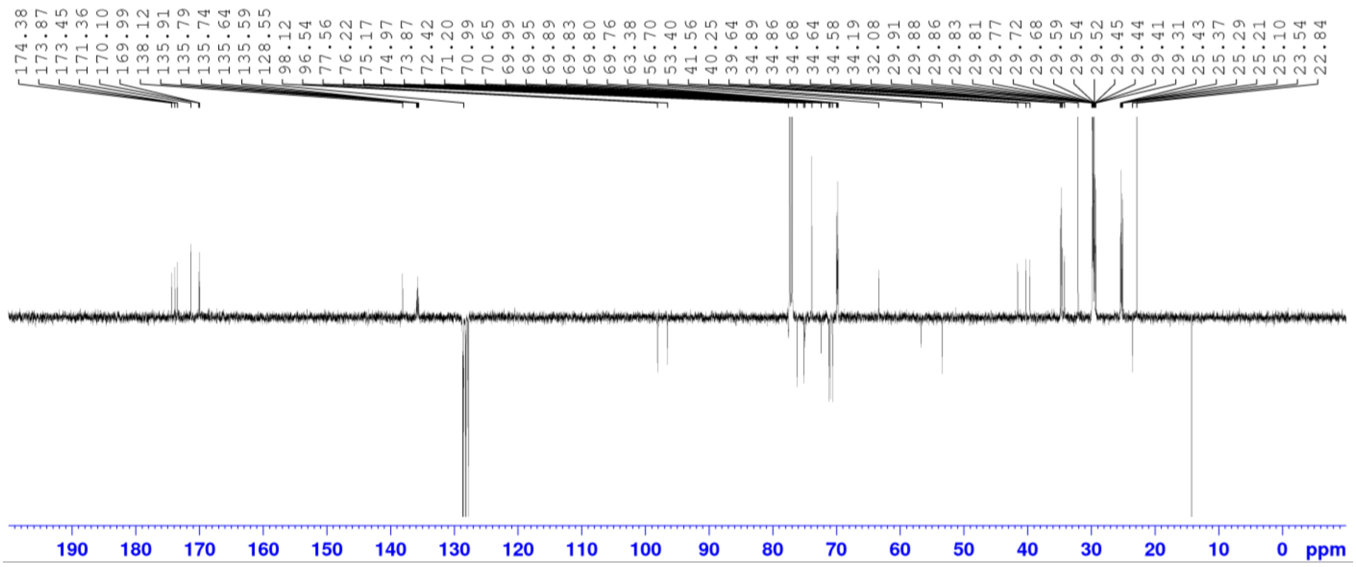

^{1}H NMR (600 MHz, $CDCl_3$)

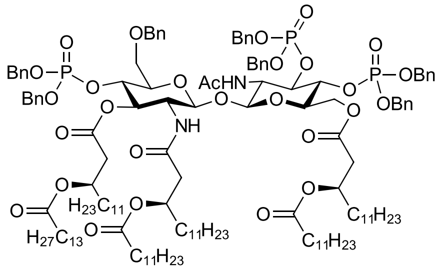

32

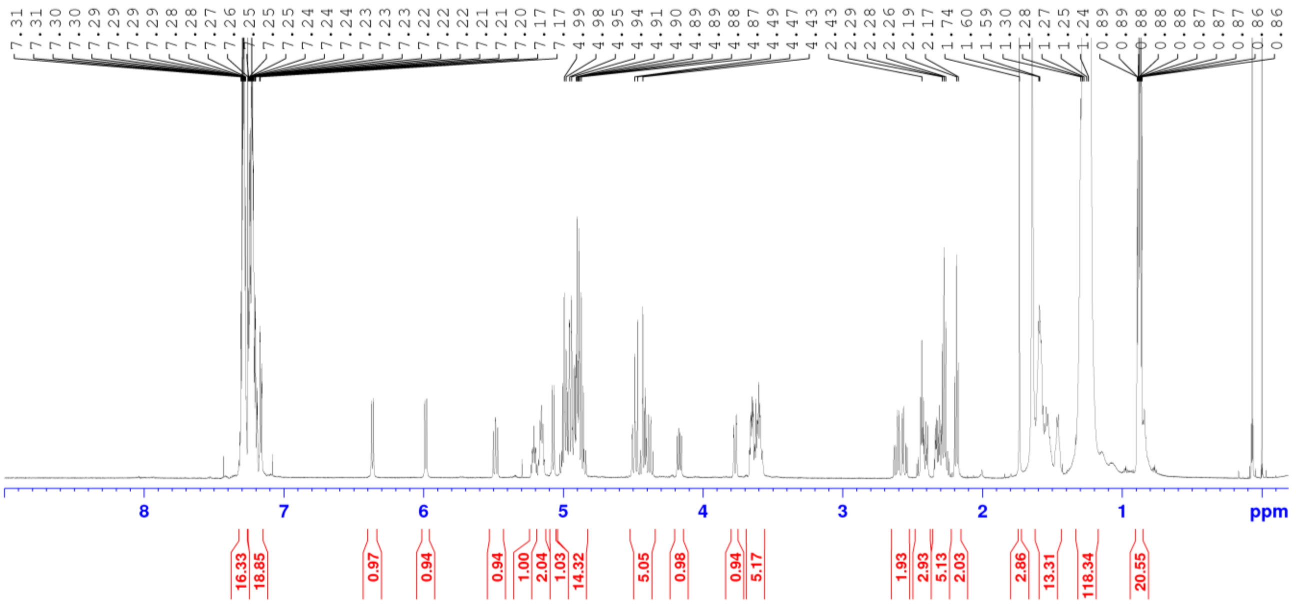

^{13}C NMR (151 MHz, $CDCl_3$)

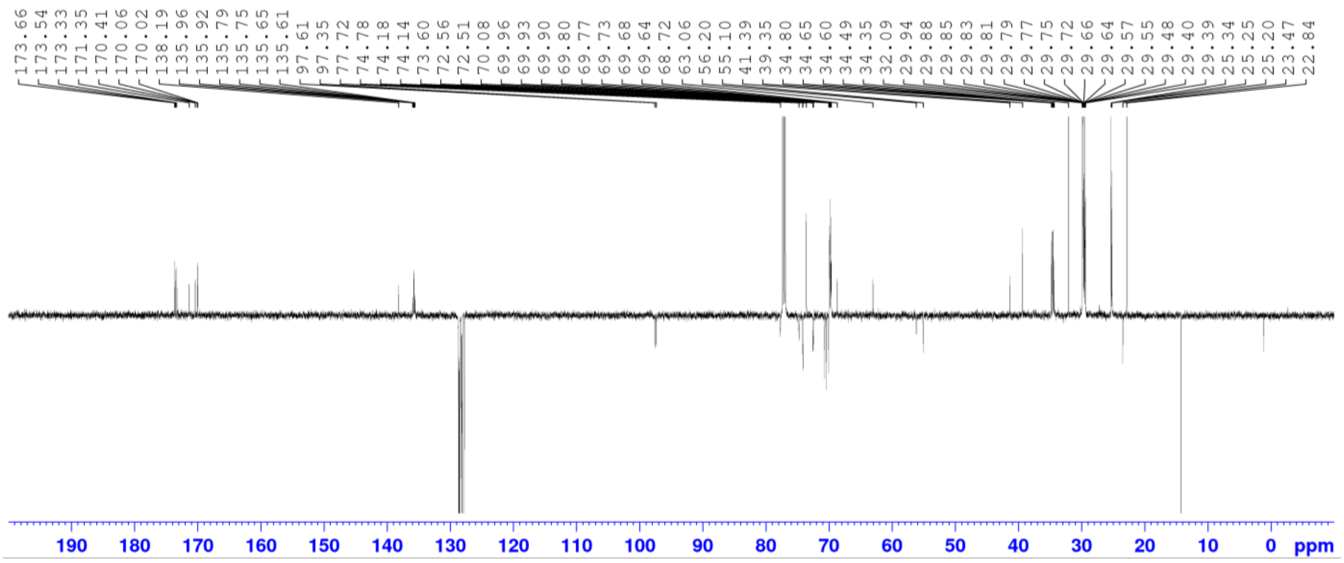

^{1}H NMR (600 MHz, MeOD / $CDCl_3$ 3:2)

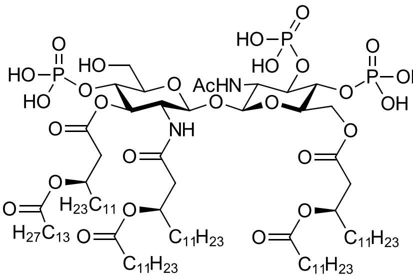

ββ-DLAM 937

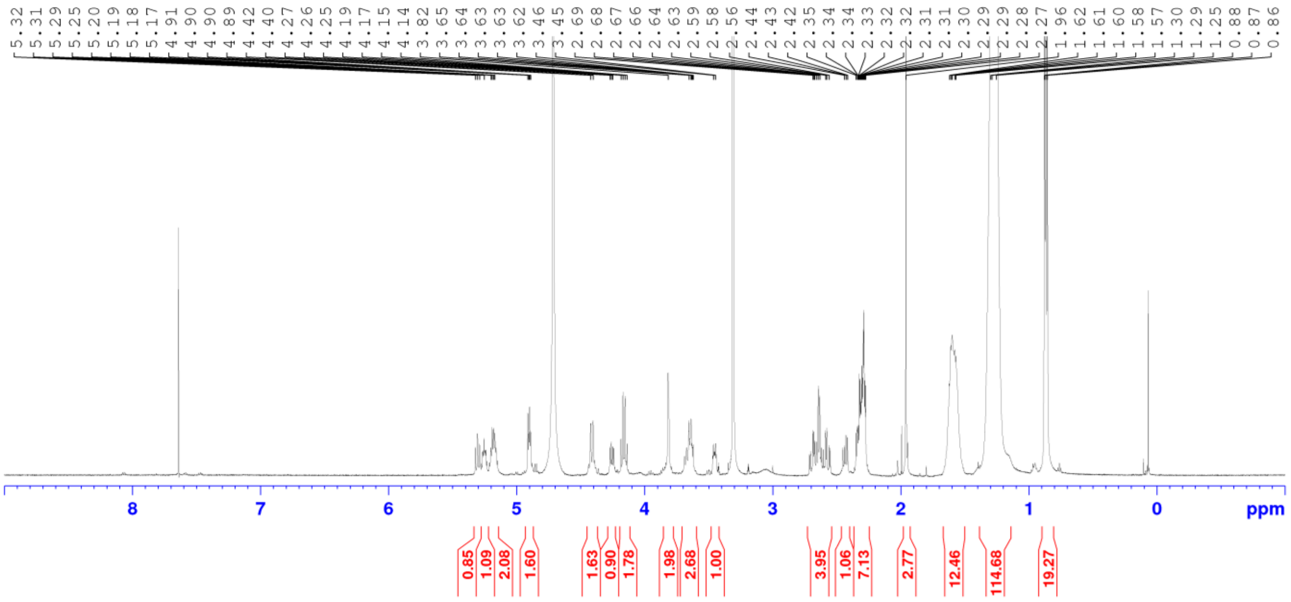

^{1}H-^{31}P HMBC (600/243 MHz, MeOD / $CDCl_3$ 3:2)

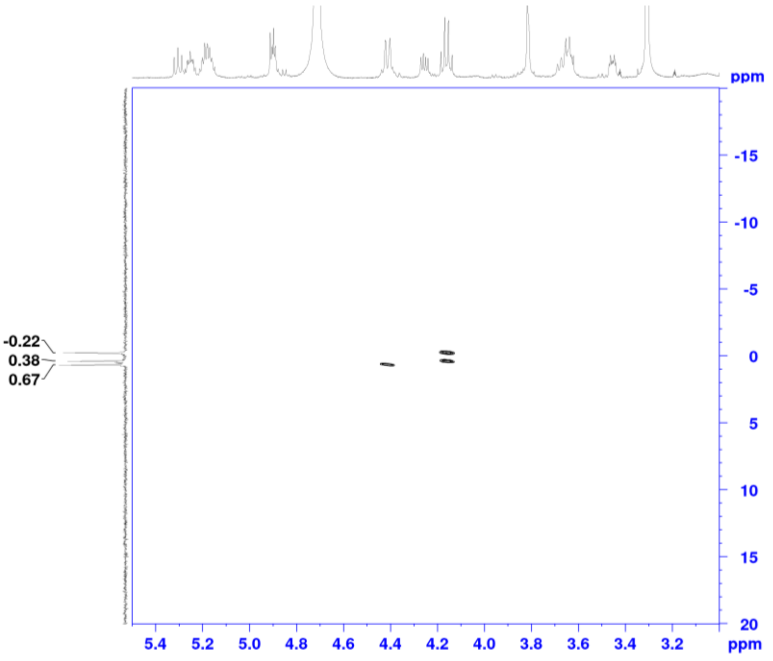

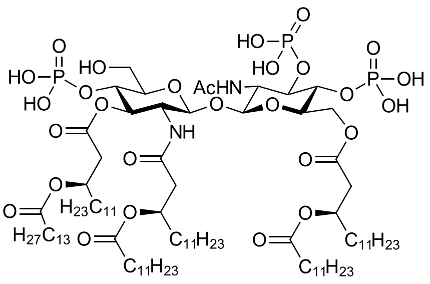

^{1}H-^{13}C HSQC (600/151 MHz, MeOD / $CDCl_3$ 3:2)

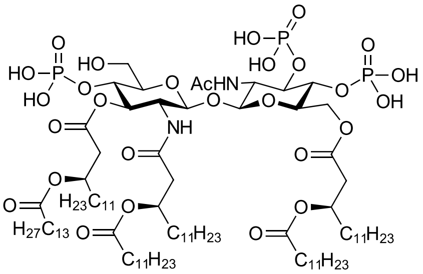

***ββ*-DLAM 937**

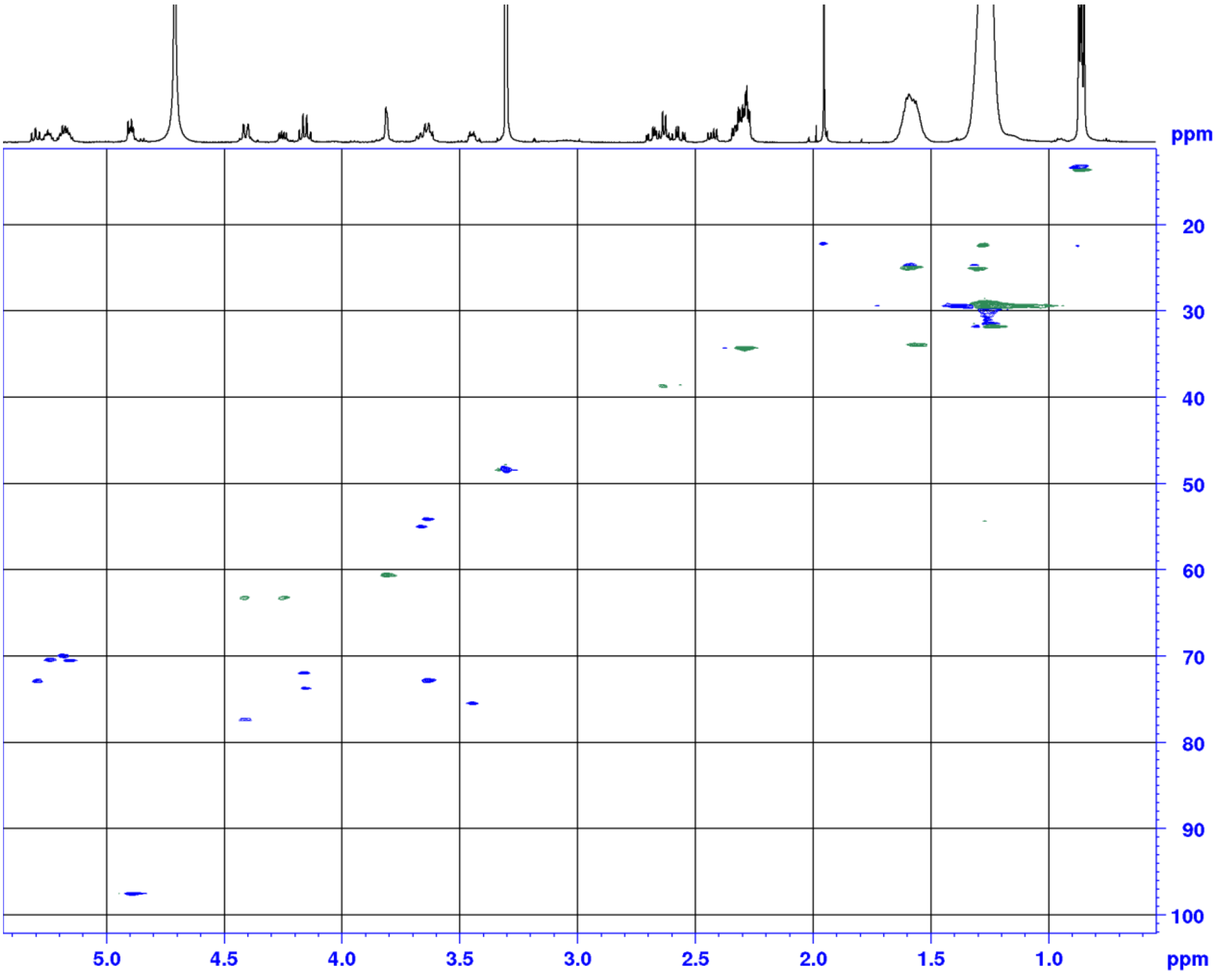

^{1}H NMR (600 MHz, $CDCl_3$)

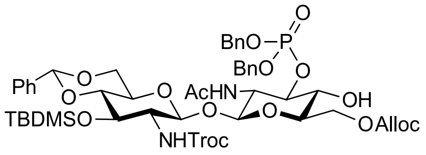

33

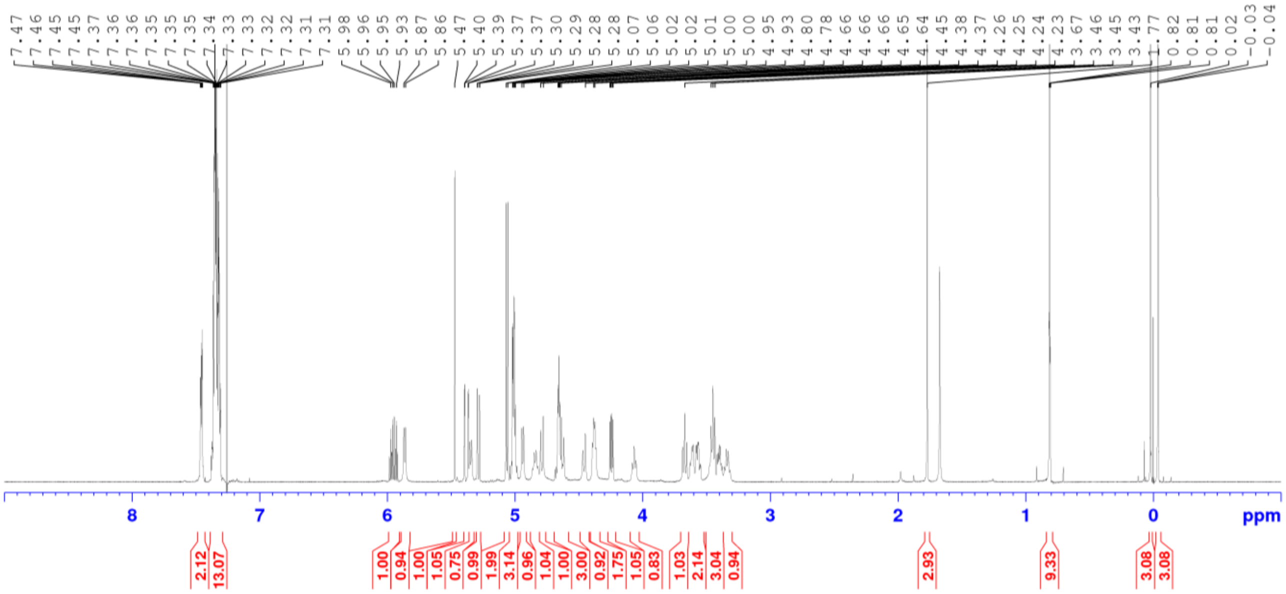

^{13}C NMR (151 MHz, $CDCl_3$)

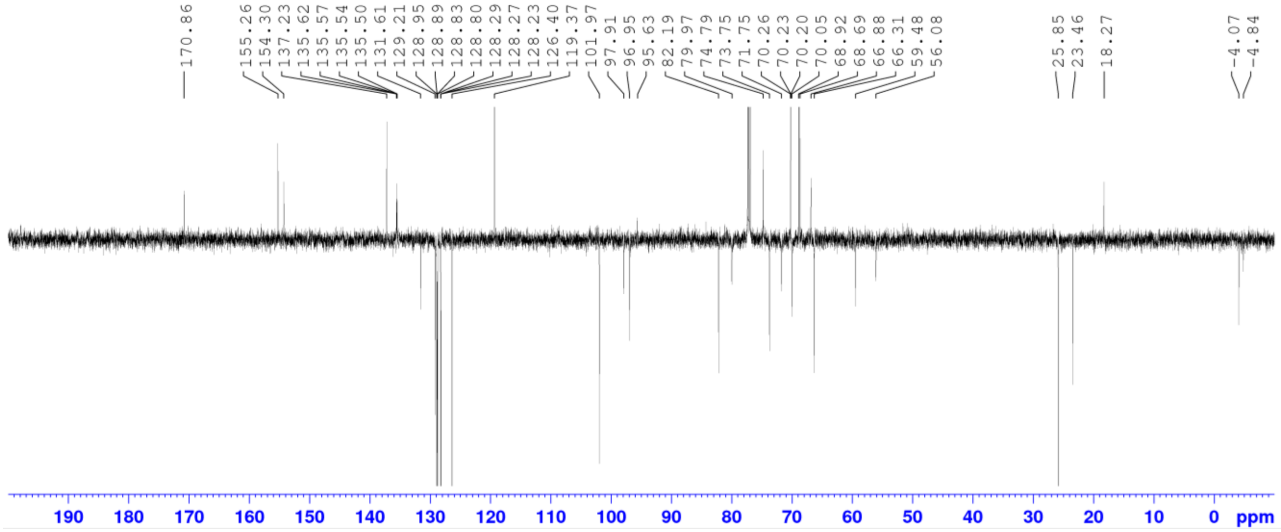

^{1}H NMR (600 MHz, $CDCl_3$)

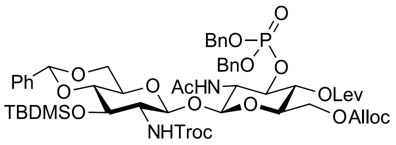

34

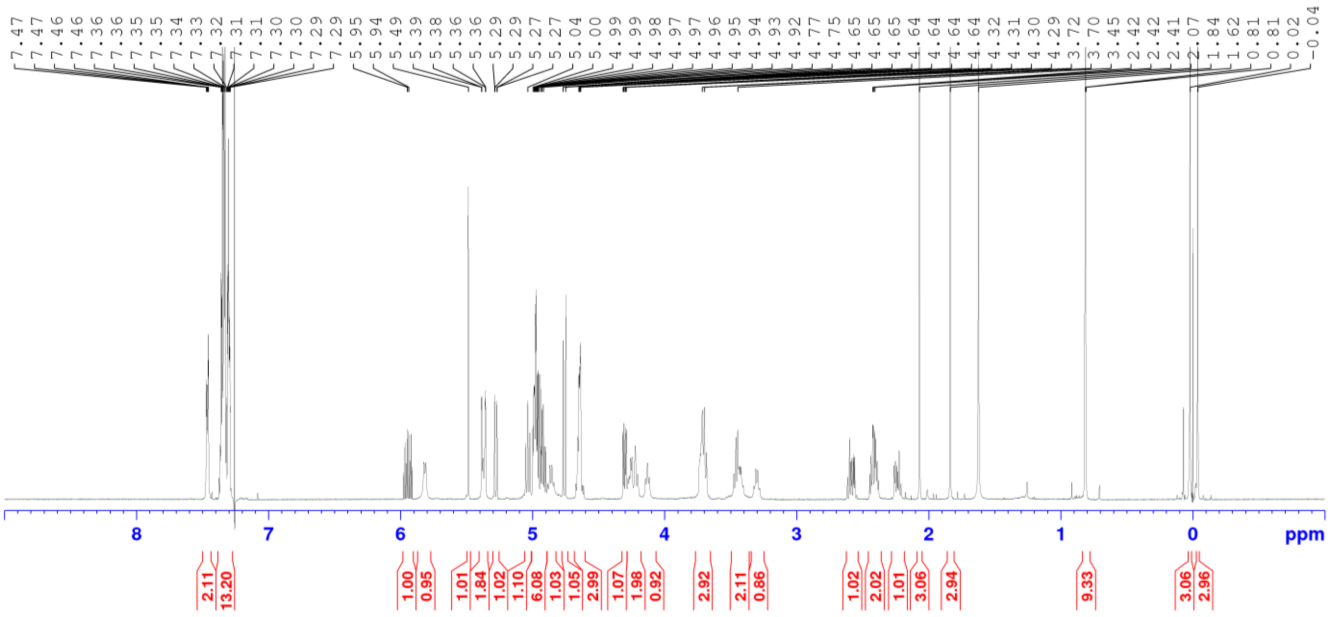

^{13}C NMR (151 MHz, $CDCl_3$)

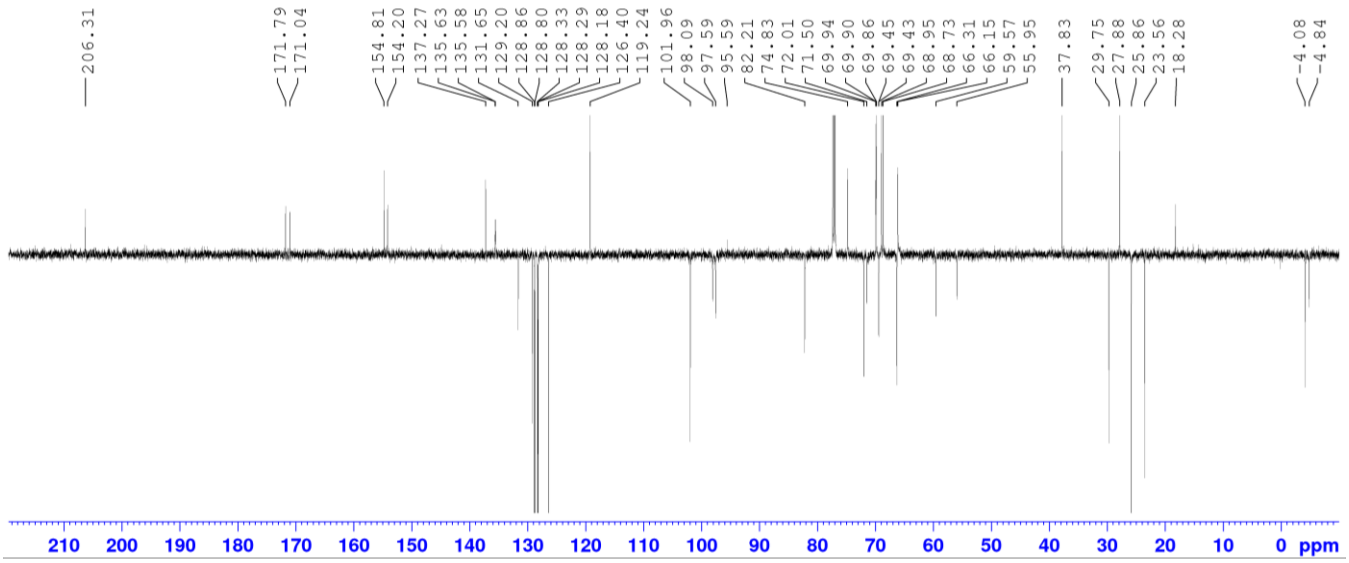

^{1}H NMR (600 MHz, $CDCl_3$)

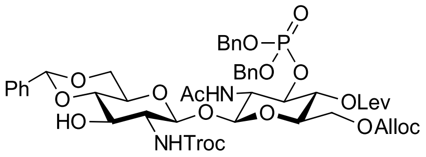

35

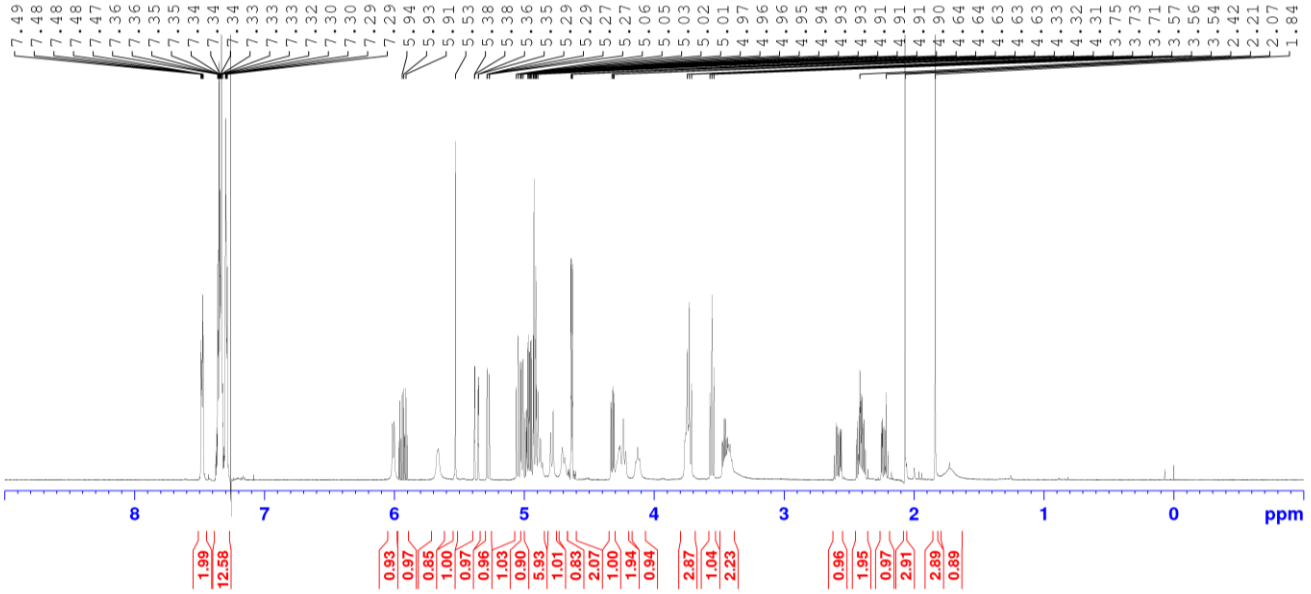

^{13}C NMR (151 MHz, $CDCl_3$)

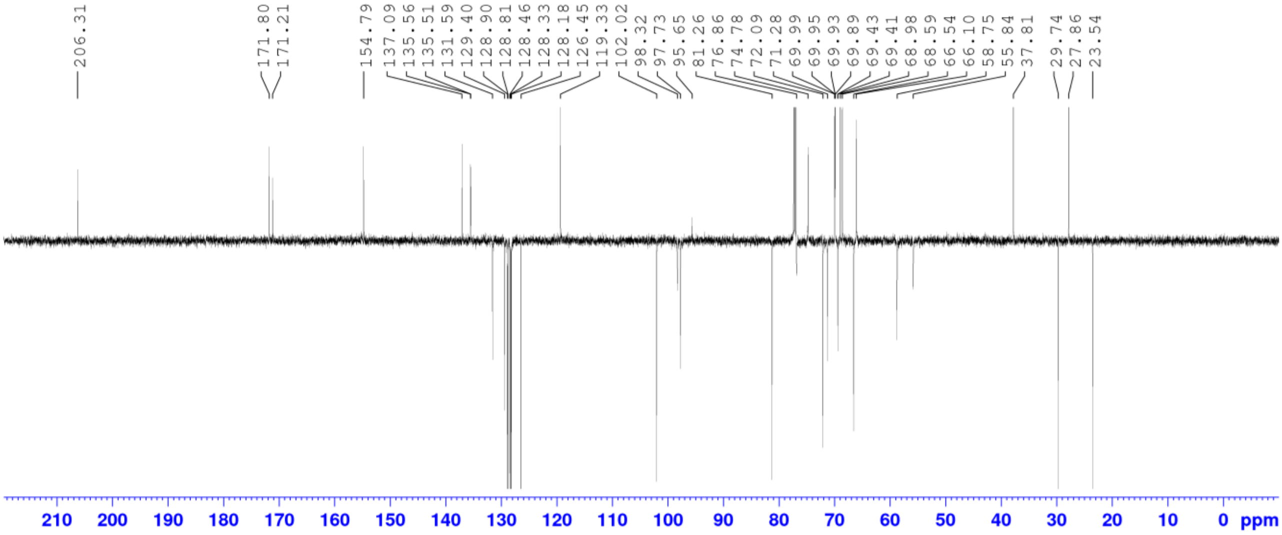

^{1}H NMR (600 MHz, $CDCl_3$)

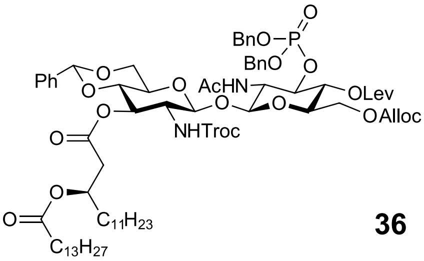

36

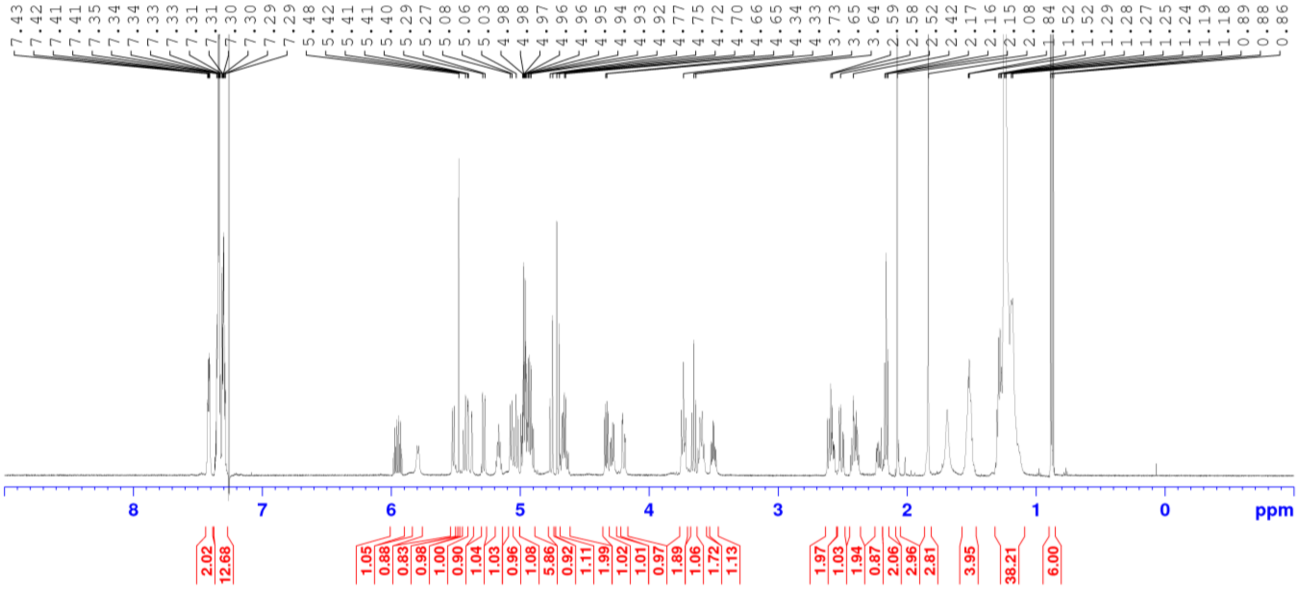

^{13}C NMR (151 MHz, $CDCl_3$)

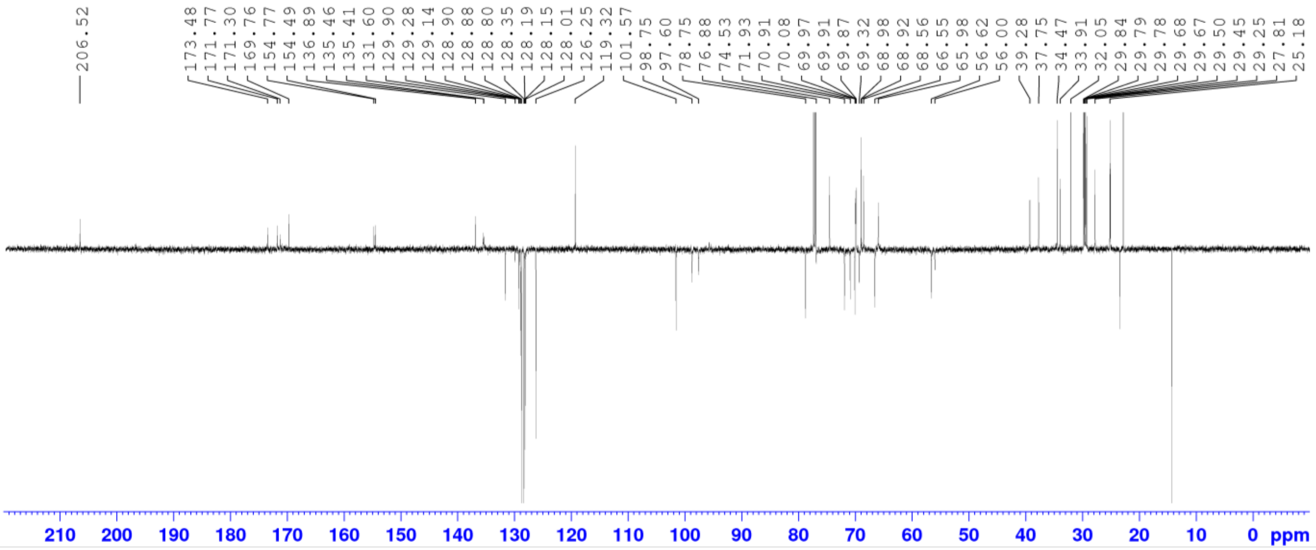

^{1}H NMR (600 MHz, CD_2Cl_2)

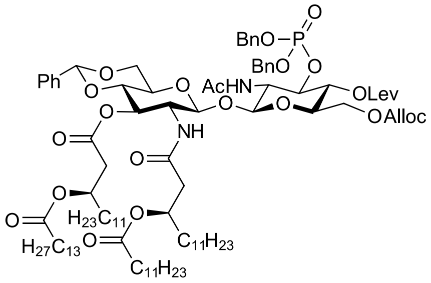

37

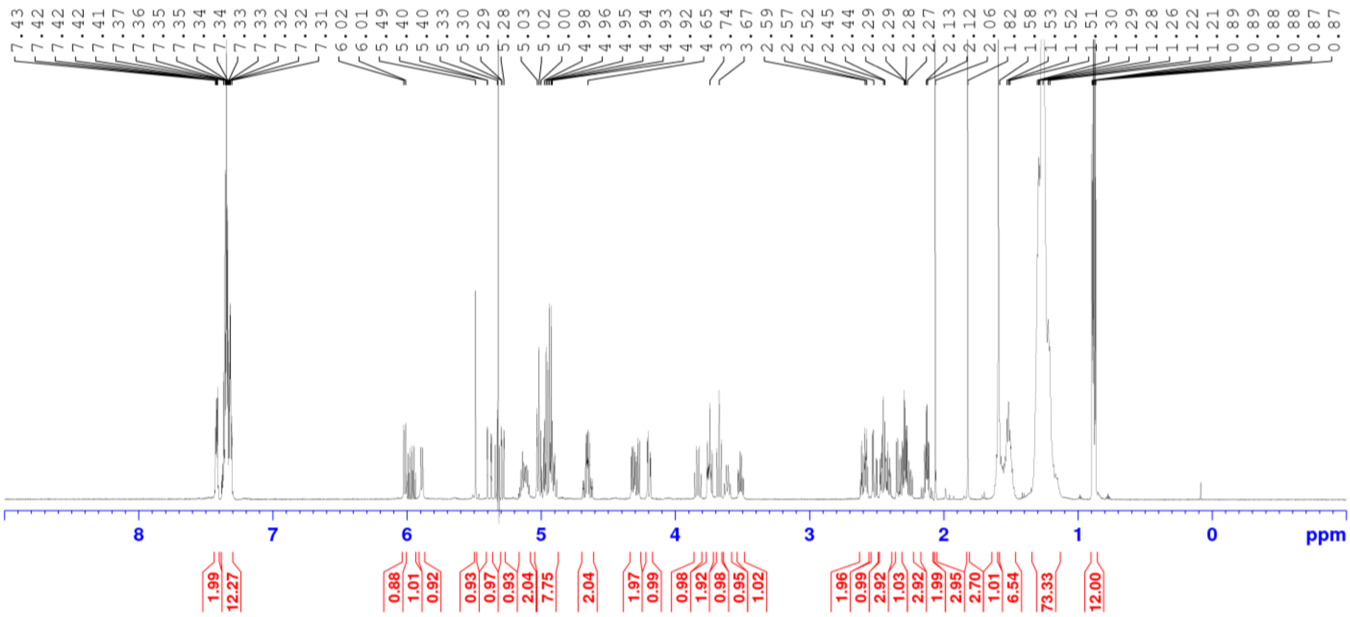

^{13}C NMR (151 MHz, CD_2Cl_2)

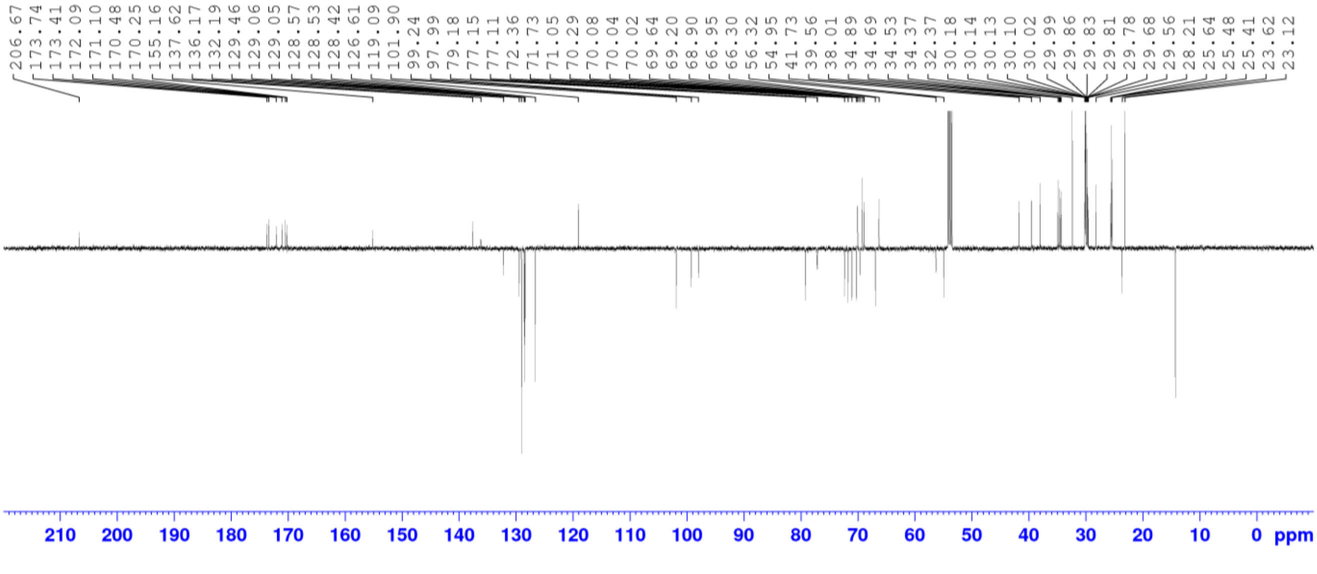

^{1}H NMR (600 MHz, CD_2Cl_2)

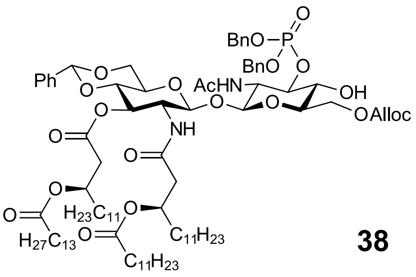

38

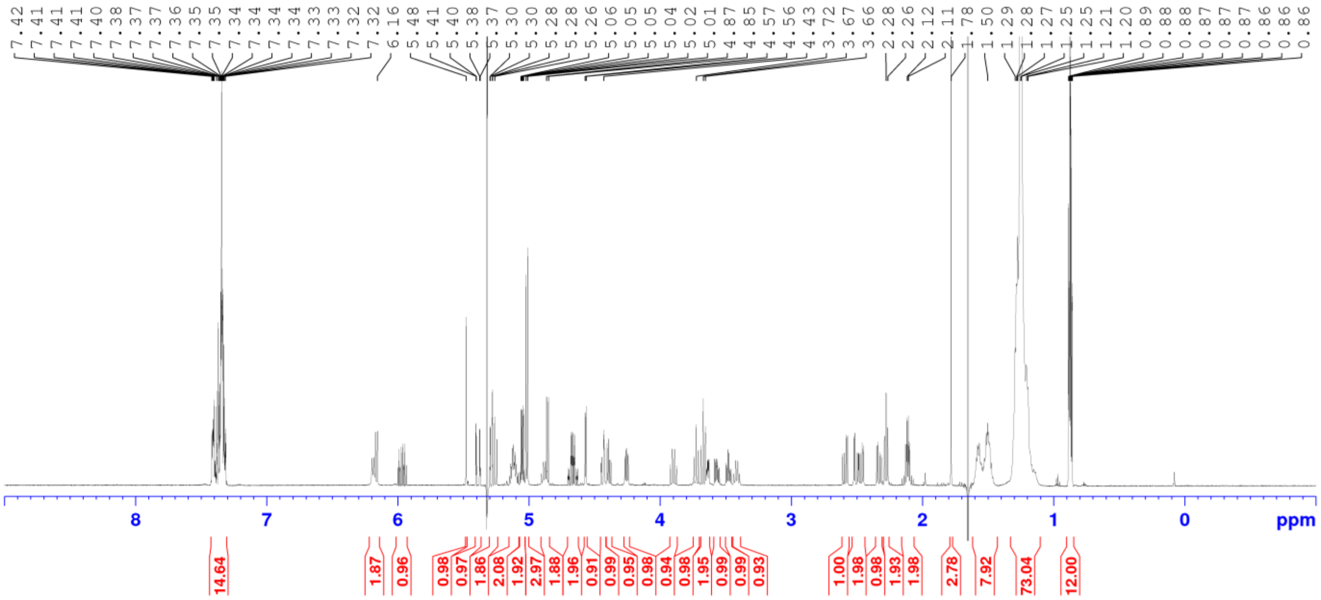

^{13}C NMR (151 MHz, CD_2Cl_2)

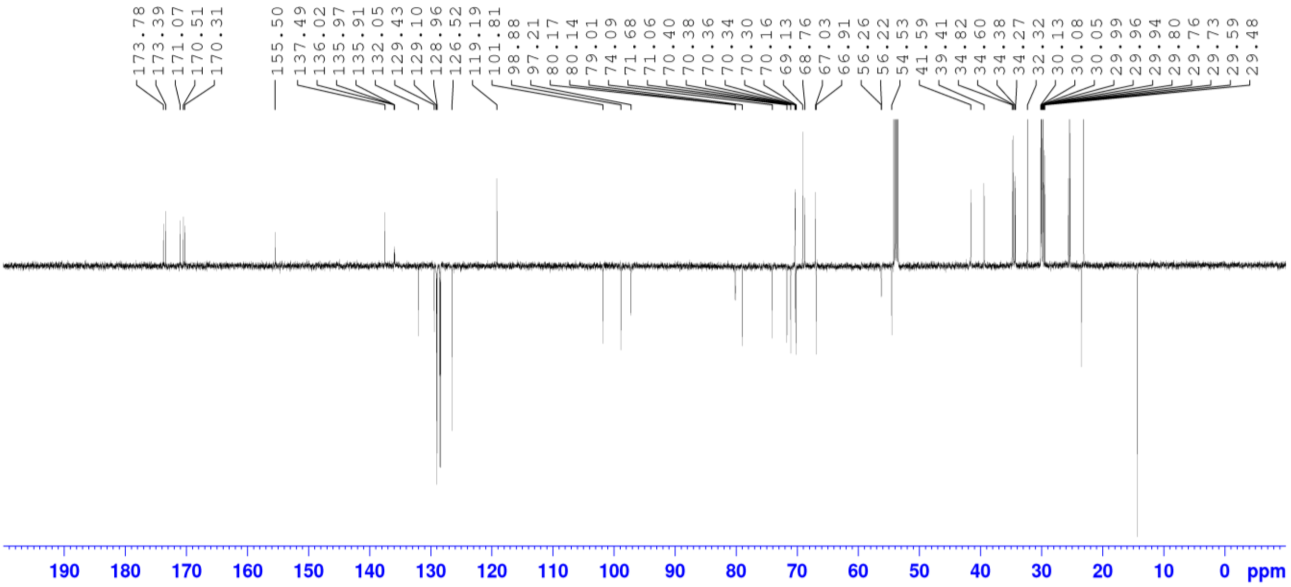

^{1}H NMR (600 MHz, CD_2Cl_2)

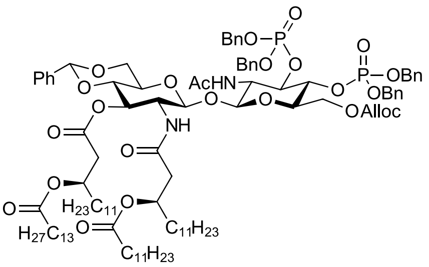

39

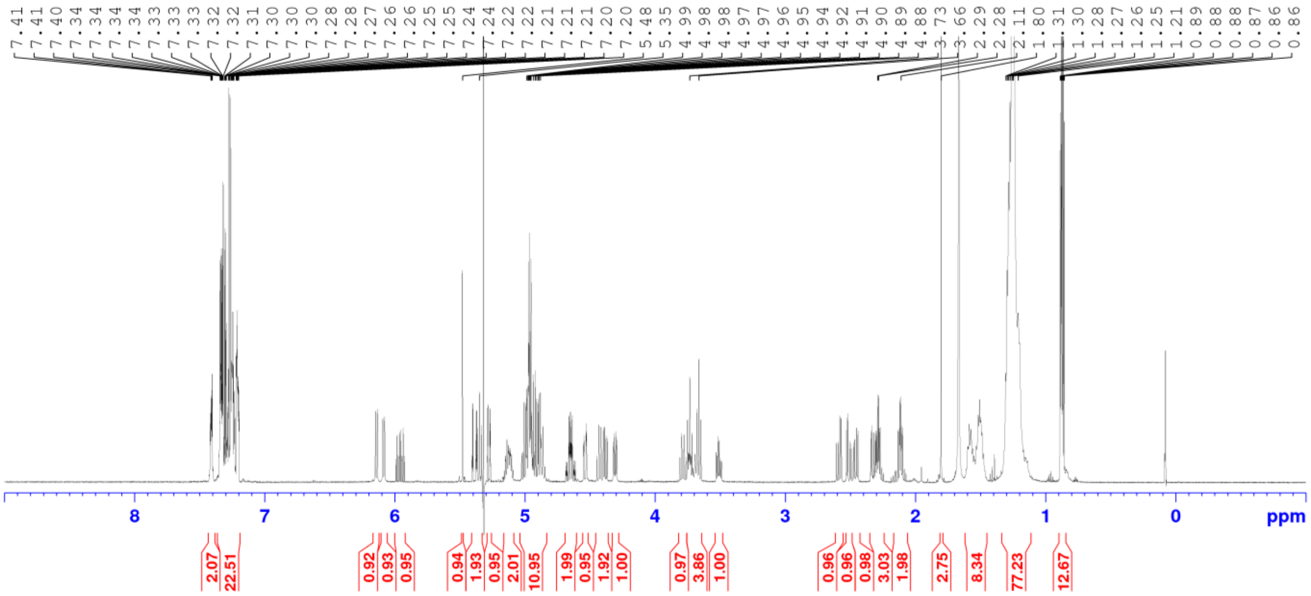

^{13}C NMR (151 MHz, CD_2Cl_2)

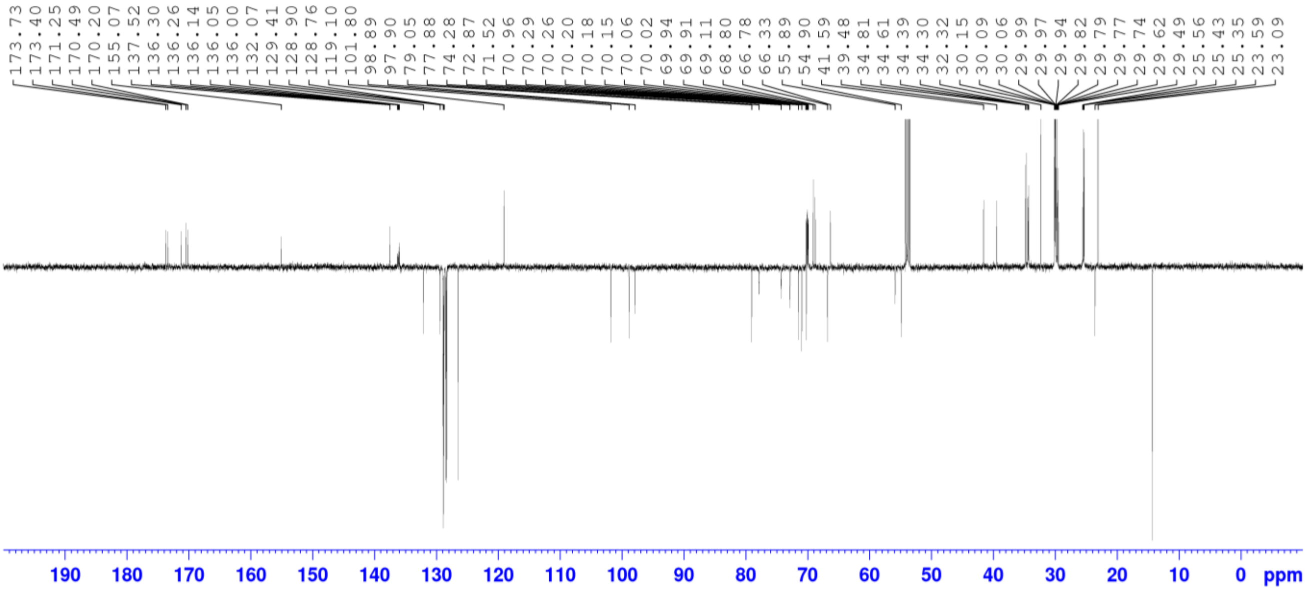

^{1}H NMR (600 MHz, CD_2Cl_2)

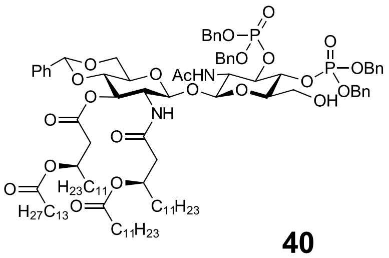

40

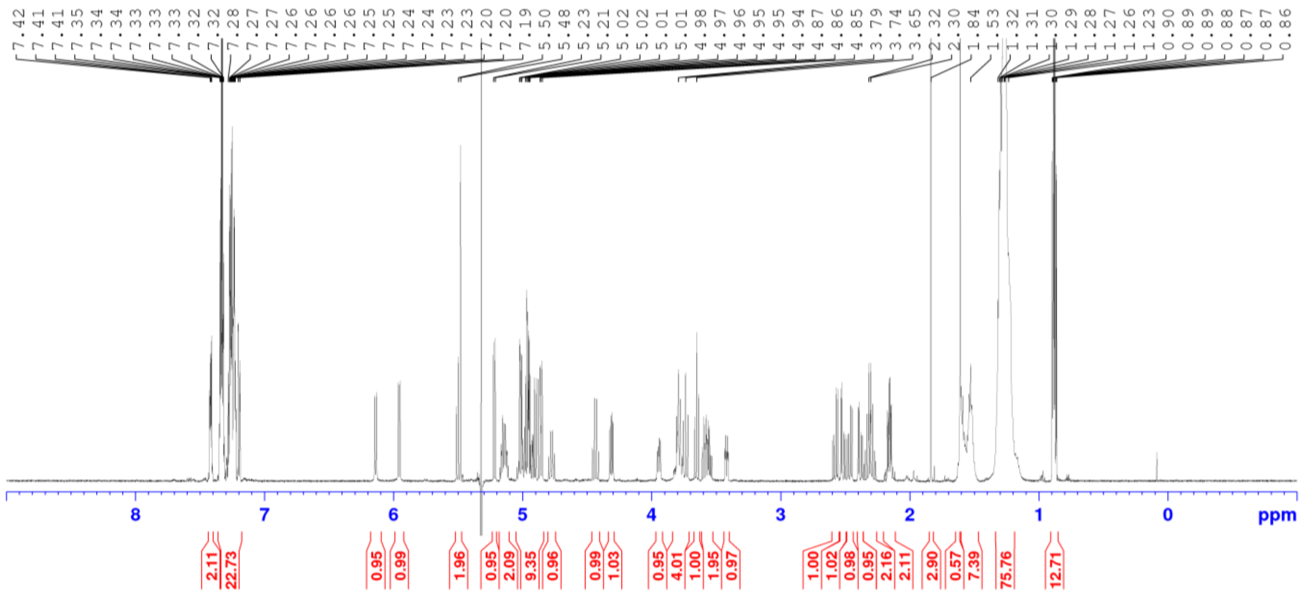

^{13}C NMR (151 MHz, CD_2Cl_2)

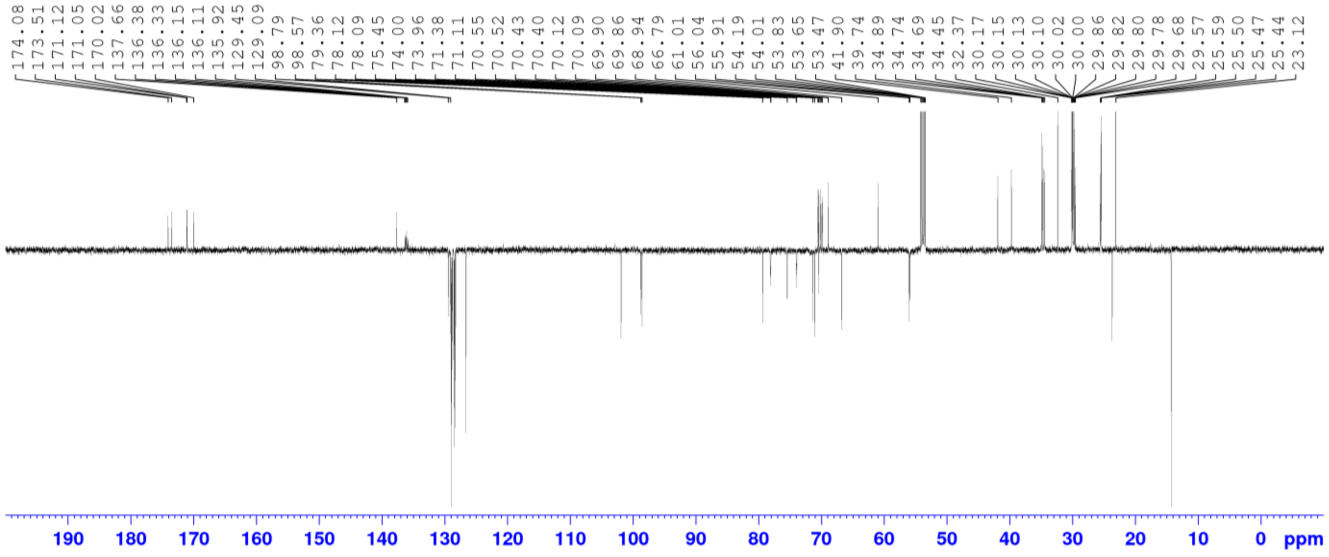

^{1}H NMR (600 MHz, CD_2Cl_2)

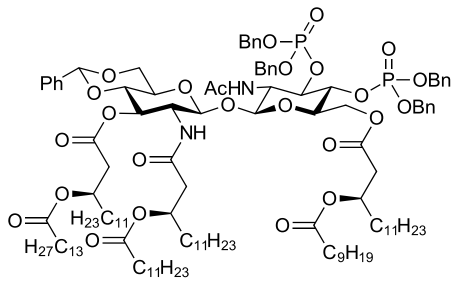

41

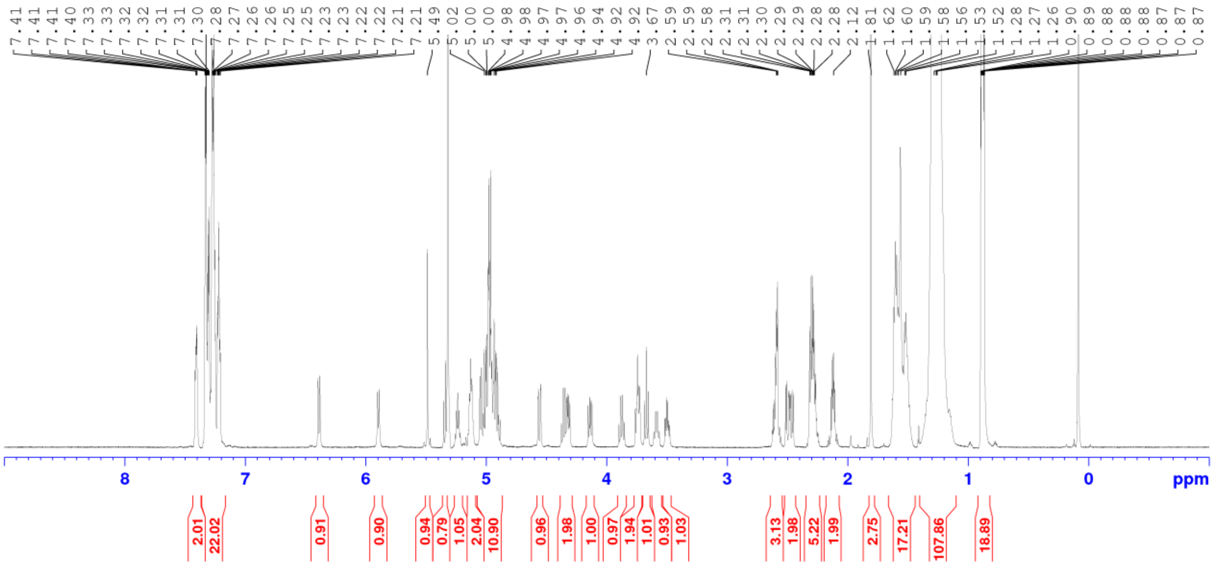

^{13}C NMR (151 MHz, CD_2Cl_2)

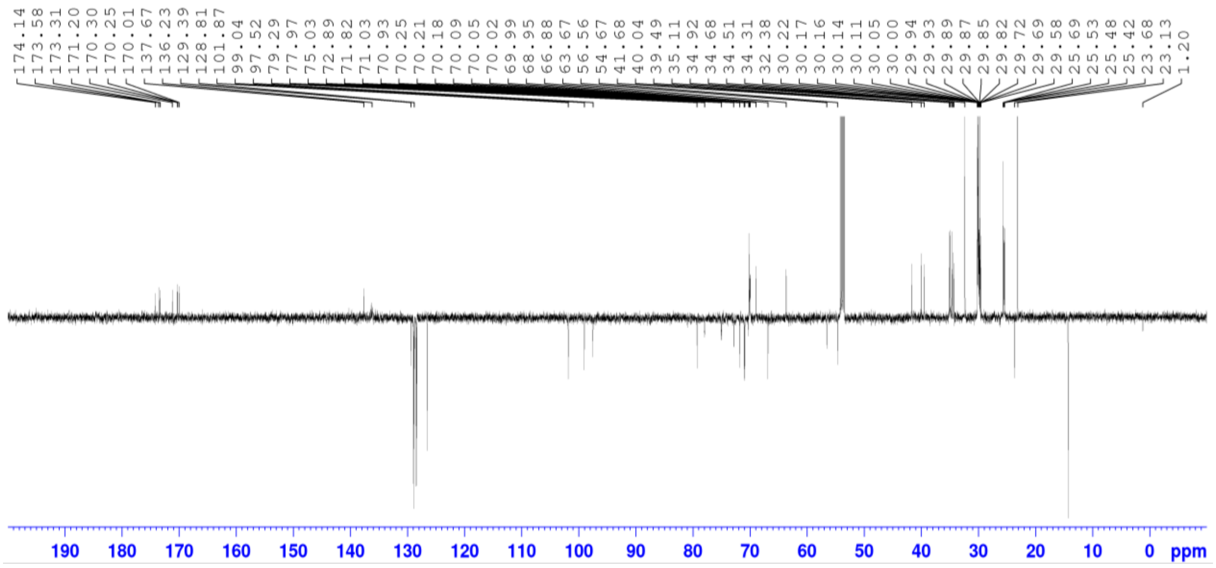

^{1}H NMR (600 MHz, CD_2Cl_2)

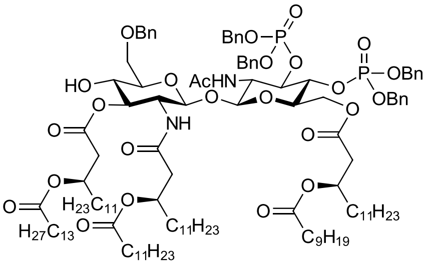

42

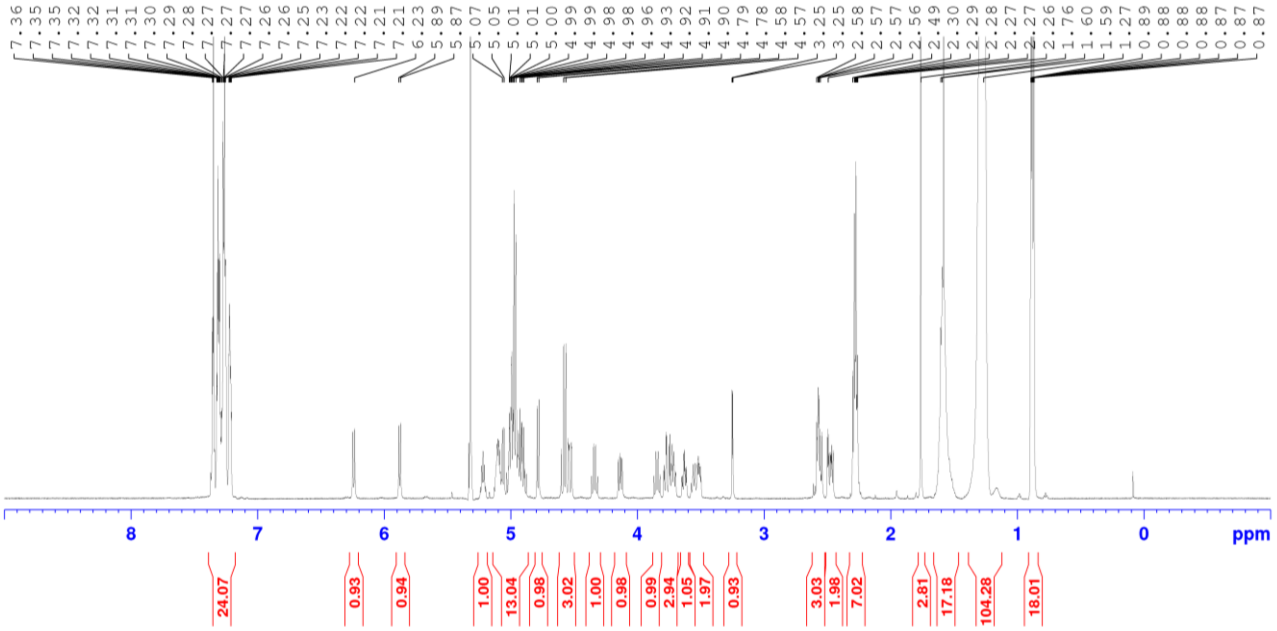

^{13}C NMR (151 MHz, CD_2Cl_2)

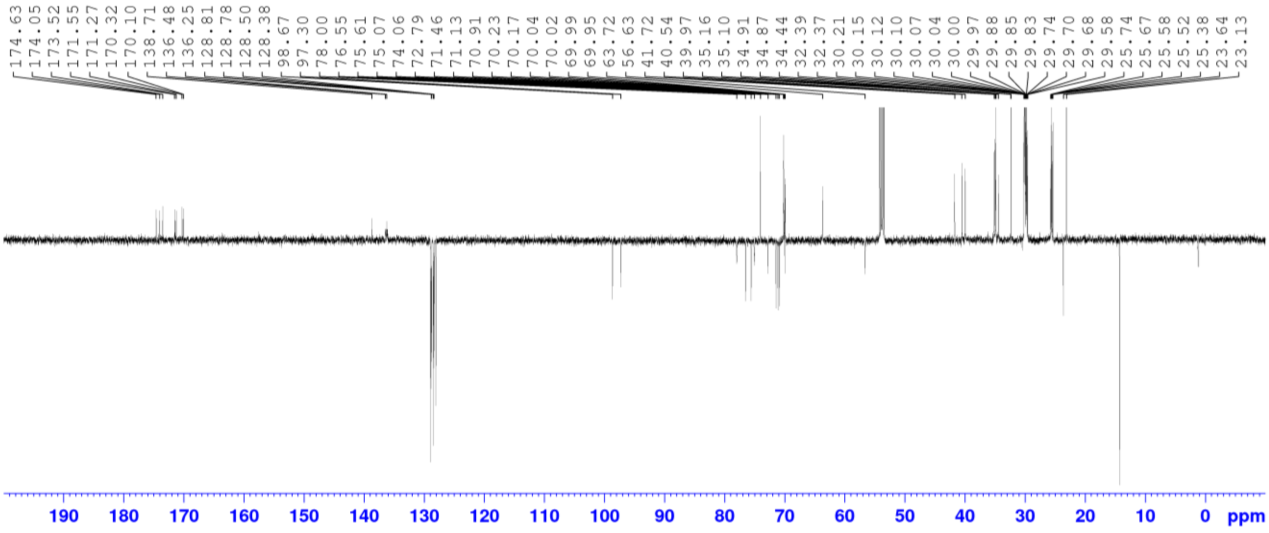

^{1}H NMR (600 MHz, CD_2Cl_2)

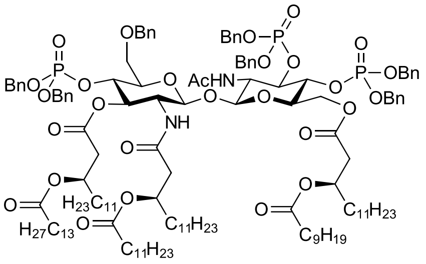

43

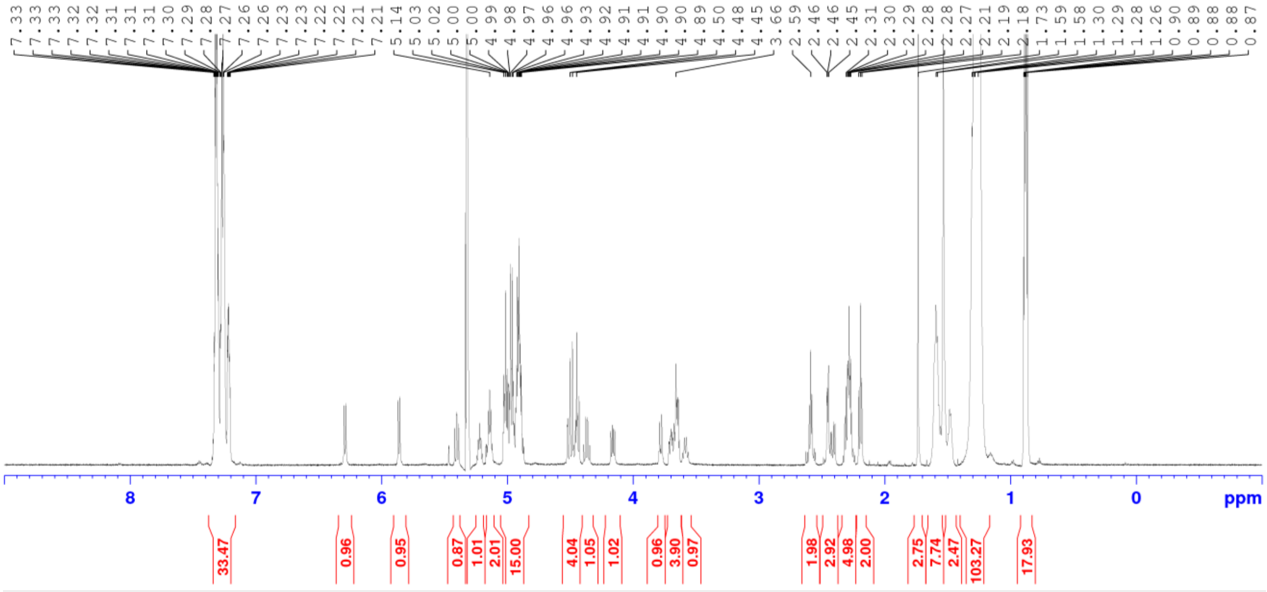

^{13}C NMR (151 MHz, CD_2Cl_2)

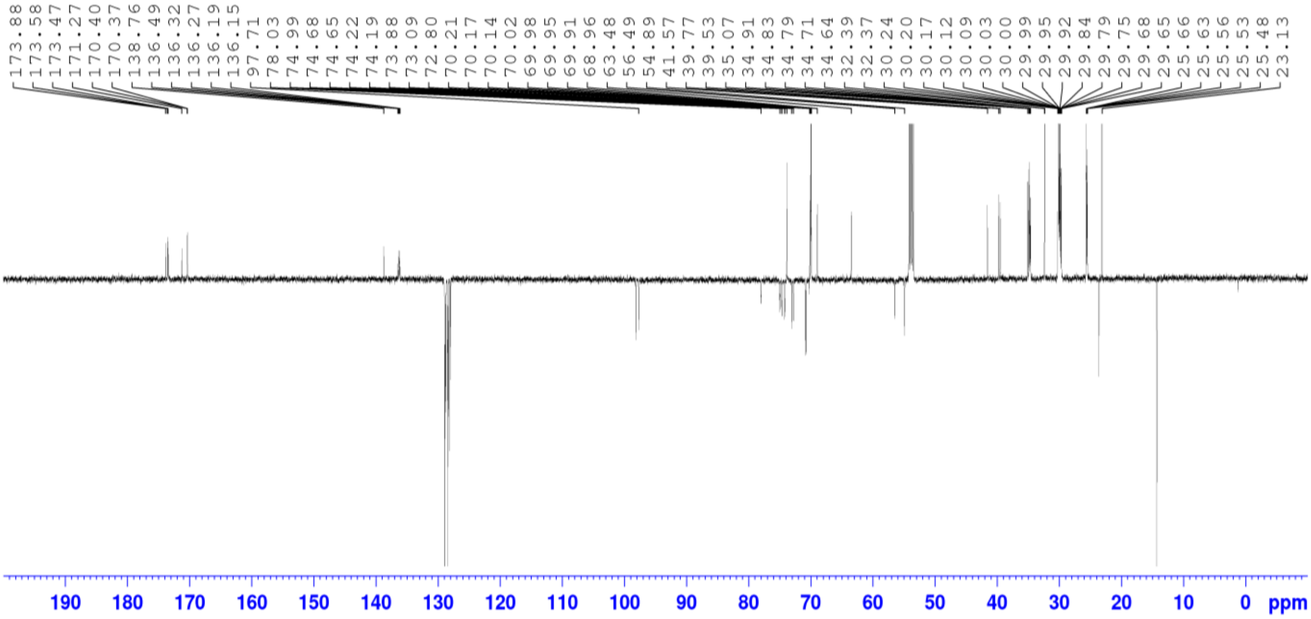

^1H NMR (600 MHz, MeOD / $CDCl_3$ 3:2, water suppression)

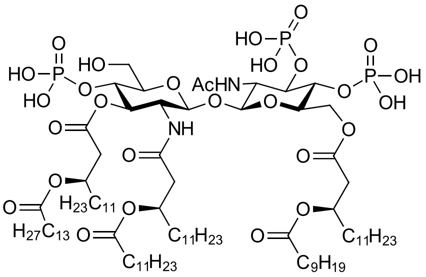

***ββ*-DLAM 3370**

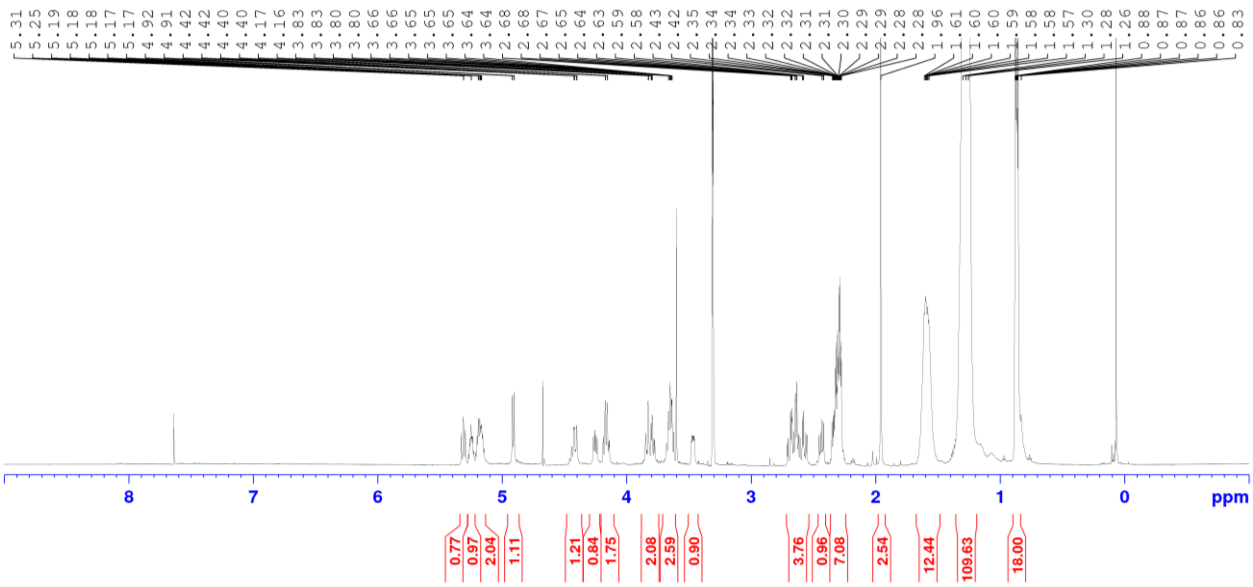

^{13}C NMR (151 MHz, MeOD / $CDCl_3$ 3:2)

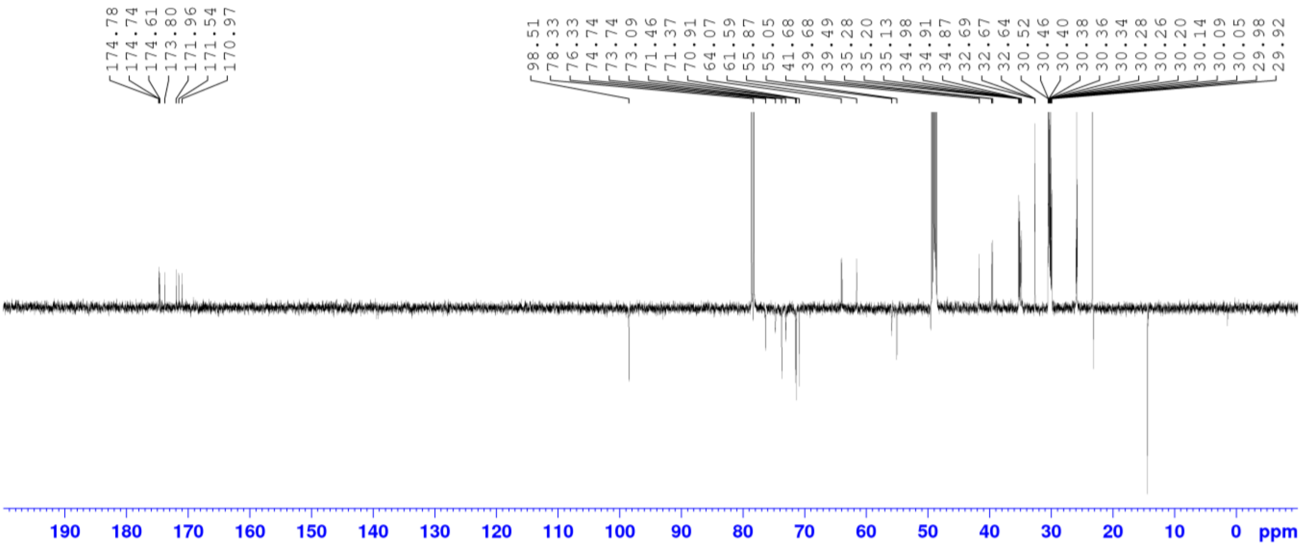

^{1}H-^{31}P HMBC (600/243 MHz, MeOD / $CDCl_3$ 3:2)

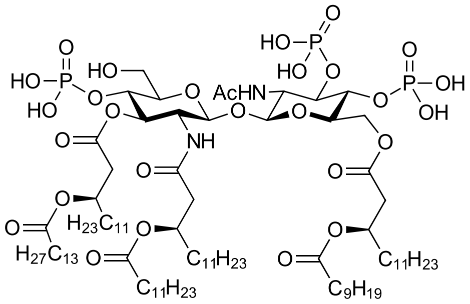

***ββ*-DLAM 3370**

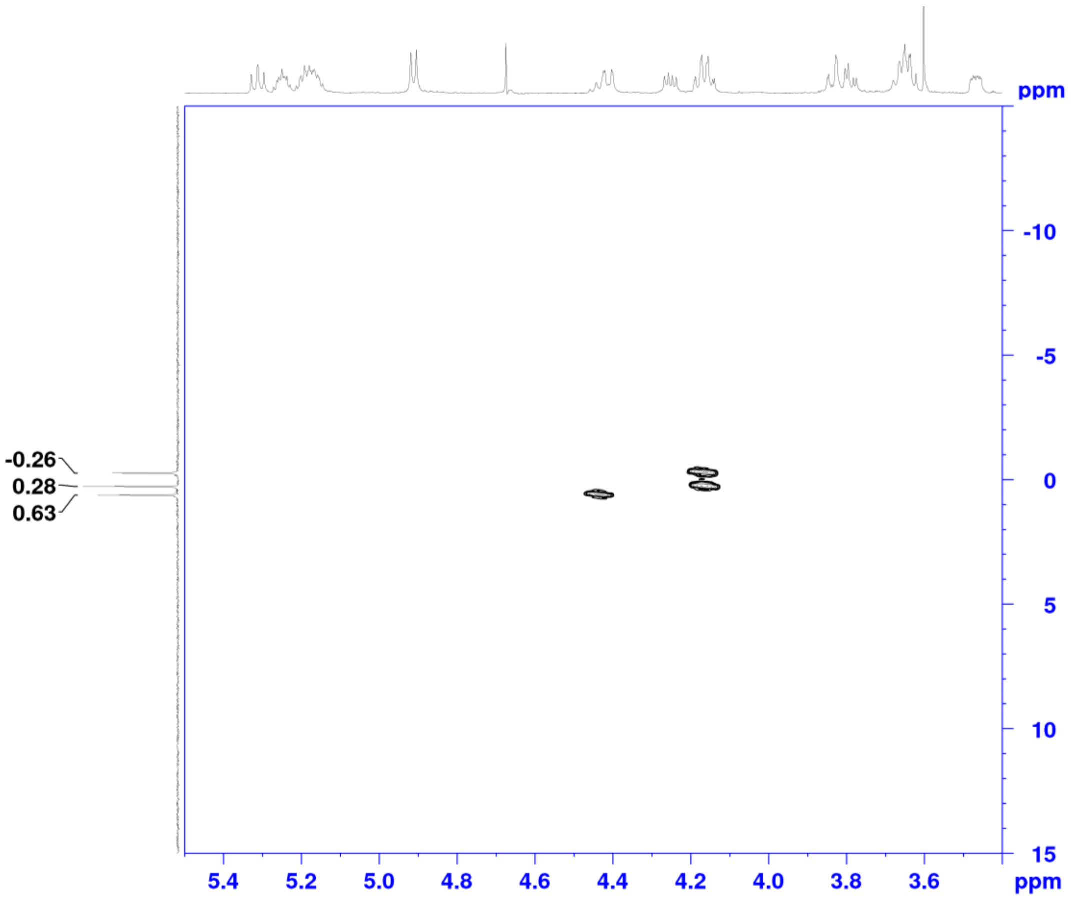

Supplement: Supplementary file 1 — Supporting Information [file CHEM-28-0-s001.pdf]
